# Closing Data Gaps for LCA of Pharmaceutical Production: Estimating Energy Usage by Upscaling Laboratory Data

**DOI:** 10.1021/acssuschemeng.5c04708

**Published:** 2025-11-14

**Authors:** Muhammed Ayaj Ansar, Rosalie van Zelm, Ad M. J. Ragas

**Affiliations:** Department of Environmental Science, Radboud Institute for Biological and Environmental Sciences (RIBES), 6029 Radboud University, Heyendaalseweg 135, 6525 AJ Nijmegen, Netherlands

**Keywords:** life cycle inventory, sustainability, carbon footprint, active pharmaceutical ingredients, prospective LCA, chemicals

## Abstract

Pharmaceutical production substantially contributes to global greenhouse gas emissions. The application of Life Cycle Assessment (LCA) to evaluate these impacts is hindered by the limited Life Cycle Inventory (LCI) data. Existing LCI estimation methods often exclude key operations such as waste treatment and tablet formulation, relying on broad assumptions that lead to incomplete assessments. To address these gaps, this study developed a method to estimate industrial energy usage by upscaling laboratory-scale data. This method includes Active Pharmaceutical Ingredient (API) synthesis, tablet formulation, and auxiliary operations. Process Design Calculations (PDCs) derived in our method improve the energy estimation for unit operations. The application of this method to six pharmaceuticals resulted in total energy estimates exceeding those of the existing methods by over 102% for Lidocaine, Diclofenac, Paracetamol, and Ibuprofen. Higher estimated energy usage led to a 3% to 49% increase in carbon footprint, primarily because operations previously left out contributed over 17% to the total carbon footprint. The new method’s energy-based carbon footprint seems to align better with industrial reference data than other methods. We conclude that our method improves the estimation of industrial energy usage for pharmaceutical production and reduces the risk of impact underestimation. It enables LCA practitioners to conduct more reliable assessments, supporting sustainability decisions.

## Graphical abstract

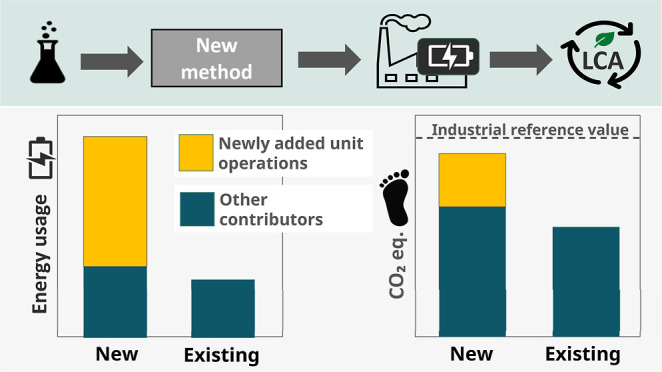

## Introduction

Over the past decade, the surge in global pharmaceutical production has raised concerns about the environmental impacts.^1^ For example, the USA healthcare sector has been held accountable for 9–10% of total national greenhouse gas (GHG) emissions, with prescription medication estimated to contribute around 10%.^2^ Similarly, in Australia, the United Kingdom, Canada, and other OECD countries, pharmaceuticals have been estimated to be responsible for 8–25% of total GHG emissions.^3,4^ These figures underscore the need to decrease the carbon footprint of pharmaceutical production. To do so, a first step is to determine the impacts and hotspots of the production process, which can be mapped by Life Cycle Assessment (LCA).

However, performing comprehensive LCAs is often hindered by the scarcity of Life Cycle Inventory (LCI) data. This applies not only to pharmaceutical products themselves but also to chemical intermediates and upstream chemicals. Consequently, pharmaceutical LCAs have largely been limited to a few products for which pharmaceutical industries have provided production data.^5^ In many cases, these products have not been named, leading to incomplete and potentially misinterpreted impact assessments and limited value for decision-making.

In the absence of industrial production data, estimation methods can be used to generate the LCI data. Chen et al.^6^ reviewed 37 pharmaceutical industry LCAs, mainly covering batch-based API synthesis and product formulation. They found that energy usage and chemical consumption are generally the largest contributors to cradle-to-gate environmental impacts. Similarly, the energy consumed during the production of upstream chemicals significantly influences the carbon footprint of these materials. This contribution further increases when additional upstream processes, such as the transportation of materials, are included. In an analysis of 21 industrial data sets on advanced and fine organic chemicals, Wernet et al.^7^ found that total energy use from both foreground and upstream processes accounted for up to 82% of the carbon footprint. These findings underscore the importance of an accurate estimation of the total energy usage and chemical consumption in assessing the environmental impacts of pharmaceutical production.

Advanced Process Design Calculations (PDCs) are recommended by Parvatker and Eckelman^8^ to appropriately allocate the energy and resource use and the emissions from each unit process. PDCs are mathematical models that rely on detailed equipment design parameters and process specifications. Parvatker and Eckelman^8^ demonstrated that advanced PDCs led to more accurate carbon footprints for ABS and Styrene, with differences of only 1–10% compared to impacts derived from primary plant data. Conversely, shortcut methods such as stoichiometry and proxy data showed impacts that were 35–50% lower than those obtained from primary plant data. However, while advanced PDCs are generally more reliable and accurate than shortcut methods in the absence of industrial data, the error values can be larger for complex pharmaceutical production consisting of more synthesis steps.

While PDCs have proven effective to estimate energy usage in industrial chemical production, they require a large number of input values. For instance, several studies have applied PDCs using equipment design parameters such as efficiency and geometry, along with process specifications like operating temperature and time.^9,10^ To address the data challenge, some LCA studies have applied the upscaling procedures proposed by Piccinno et al.^11^ using accessible laboratory process data and integrating those with PDCs for energy usage estimation.^12,13^ This procedure incorporates standardized equipment design parameters relevant to a range of production scales by adapting and simplifying them for industrial-scale applications within a LCA context.

LCA studies on pharmaceuticals typically do not include product formulation and auxiliary unit operations in pharmaceutical production, such as equipment cleaning, waste treatment, and Heating, Ventilation and Air Conditioning (HVAC). These unit operations could have significant environmental burdens.^14,15^ Instead, these studies typically rely on rough estimations of the energy consumption in those operations. Furthermore, the PDCs developed by Piccinno et al.^11^ lack empirical data on equipment design parameters and process specifications. As a result, most studies fail to capture the complexities and real-world performance variations of industrial-scale processes.

These limitations, including the reliance on rough estimates, the omission of specific equipment parameters, and the exclusion of certain unit operations, can lead to underestimation of the environmental impacts of pharmaceutical production. Given these limitations and the specialized requirements of each chemical subsector, a tailored approach is needed for the pharmaceutical sector to convert lab-scale data into realistic representations of industrial-scale processes. This requirement also falls within the context of prospective LCA (pLCA) defined here as the application of LCA to technologies not yet fully industrialized where technology scaling and scenario analysis are applied to generate foreground inventories.

This paper presents a novel method to estimate industrial energy usage for the batch production of pharmaceuticals, requiring minimal data input and being applicable to both API synthesis and solid oral dosage forms together with auxiliary operations. The method is based on upscaling lab-scale processes to the industrial plant scale. A generalized industrial process flow diagram is provided to support the identification of industrial unit operations involved in the production of the pharmaceuticals. Furthermore, guidelines are provided for estimating the energy usage of each unit operation (UOs) using PDCs. In the pLCA context, our method contributes by offering a structured way to scale laboratory pharmaceutical processes to the industrial level while explicitly accounting for auxiliary operations and enabling scenario-based evaluation of energy use. We applied our method to estimate energy usage and evaluate the carbon footprint of six selected pharmaceutical products. The results are compared with those of the other estimation methods.

## Materials and Methods

### Proposed Stepwise Procedure to Estimate Energy Usage

Figure 1 outlines the step-by-step procedure to estimate the energy usage of industrial pharmaceutical production based on lab-scale study protocols (e.g., scientific articles, patents, or industry reports).

**Figure 1:**
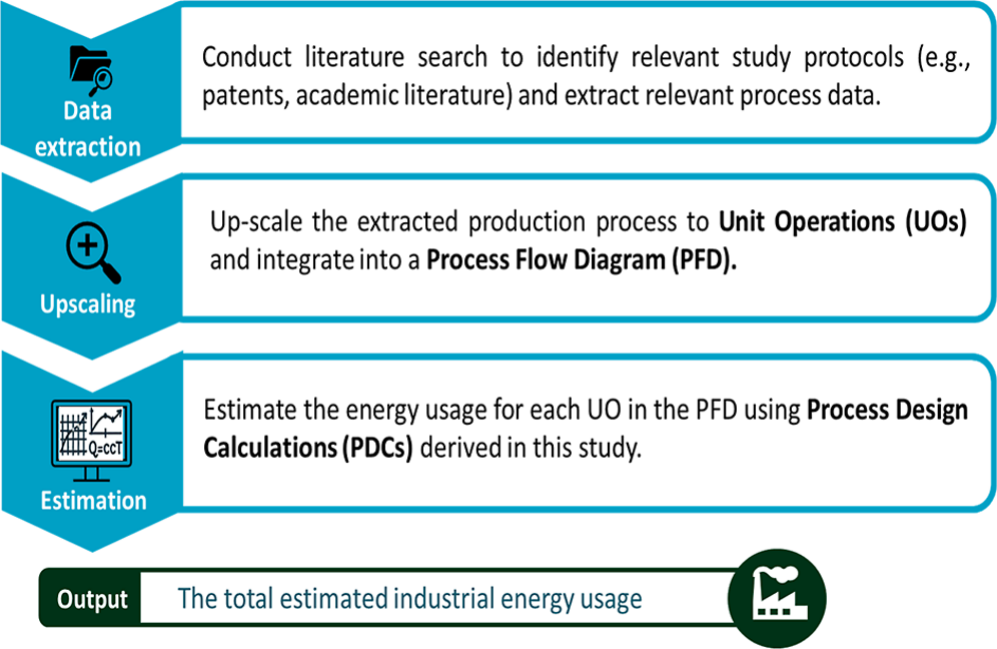
Stepwise procedure to estimate the energy usage of pharmaceutical production.

This procedure was adapted from the framework proposed by Piccinno et al.^11^ to align with the specific requirements and practices of pharmaceutical production. Compared to standard scale-up workflows in the literature, our procedure diverges in three ways: (i) laboratory steps were mapped to industrial UOs specifically tailored to pharmaceutical production; (ii) auxiliary operations such as HVAC, cleaning, and emission controls were explicitly included; and (iii) energy usage was estimated through PDCs, with dedicated guidelines and a spreadsheet tool that allow adaptation to complex operational conditions and equipment-specific parameters. The procedure consists of three steps:

1. Data extraction: start with a literature search using patent databases and peer-reviewed articles to identify the study protocols for API synthesis and tablet formulation. Use patent databases like *Google Patents* and *Espacenet* to ensure broad applicability and usability for LCA practitioners. When accessible, incorporate additional resources such as Scopus and relevant books on pharmaceutical synthesis and formulation.^16,17^ Consider using specialized tools like Reaxys^18^ as a supporting source to gain insights into reaction mechanisms and detailed experimental conditions, which can help verify the identified protocols. Extract data from study protocols, including the steps involved in API synthesis and tablet formulation, as well as process specifications such as material inputs, yields, and reaction conditions. When multiple production routes are found, consider the following criteria for selecting a synthetic route: (i) industrial relevance: routes documented in patents or studies that demonstrate industrial or pilot-scale feasibility rather than purely theoretical or exploratory academic syntheses; (ii) recency and regulatory alignment: more recent routes that reflect current practices and constraints; (iii) data availability: sufficient detail on inputs, yields, and conditions for inventory development; (iv) safety and environmental feasibility: avoid routes involving highly hazardous reagents, obsolete solvents, or rare/expensive chemicals unlikely to be adopted at scale. Expert consultation should be considered when selecting among multiple routes.
2. Upscaling and compilation: first, upscale the extracted API synthesis and tablet formulation steps to the industrial level by assigning each laboratory operation to the most representative UO in terms of equipment and function. To support this procedure, Table 1 lists common laboratory steps alongside their industrial counterparts. A worked example of the mapping procedure is provided in the Supporting Information 1 (Tables S1 and S2). Second, integrate the mapped UOs into a Process Flow Diagram (PFD) to illustrate how these operations interlink. In some cases, multiple UOs can perform the same step, allowing flexibility in choosing the appropriate operation. To support the development of a product-specific PFD, a generalized PFD was developed presenting the UOs typically involved in the industrial production of a pharmaceutical product (Figure 2; explained in the following section). Finally, upscale all material inputs and outputs extracted in step 1 to the industrial plant level. In the absence of case-specific details, it is assumed that reactants, solvents, and yields extracted from lab procedures scale up linearly in stoichiometric quantities (Supporting Information 1: Table S3). A spreadsheet was developed to support scaling to the selected industrial batch scale and to verify mass balances (Supporting Information 2).
3. Energy estimation: estimate the expected energy usage for each UO-using PDCs. Use the upscaled data from step 2 (e.g., material inputs and reaction temperatures) as input for the PDCs, along with the suggested equipment design parameters for each UO. The main equations used in the PDCs are presented in Table 2. This is also accompanied by alternative simplified approaches for cases where data on specific process parameters (e.g., processing times or temperatures) are unavailable. Quantify energy usage as thermal energy (MJ) and electricity (kWh) for the entire production process. Detailed guidelines are provided in Sections 4–6 of the Supporting Information 1 and briefly discussed below. These guidelines offer the necessary depth for handling complex operational conditions, adapting PDCs, and accounting for specific equipment specifications, such as reactor sizes and heating mechanism.

**Figure 2:**
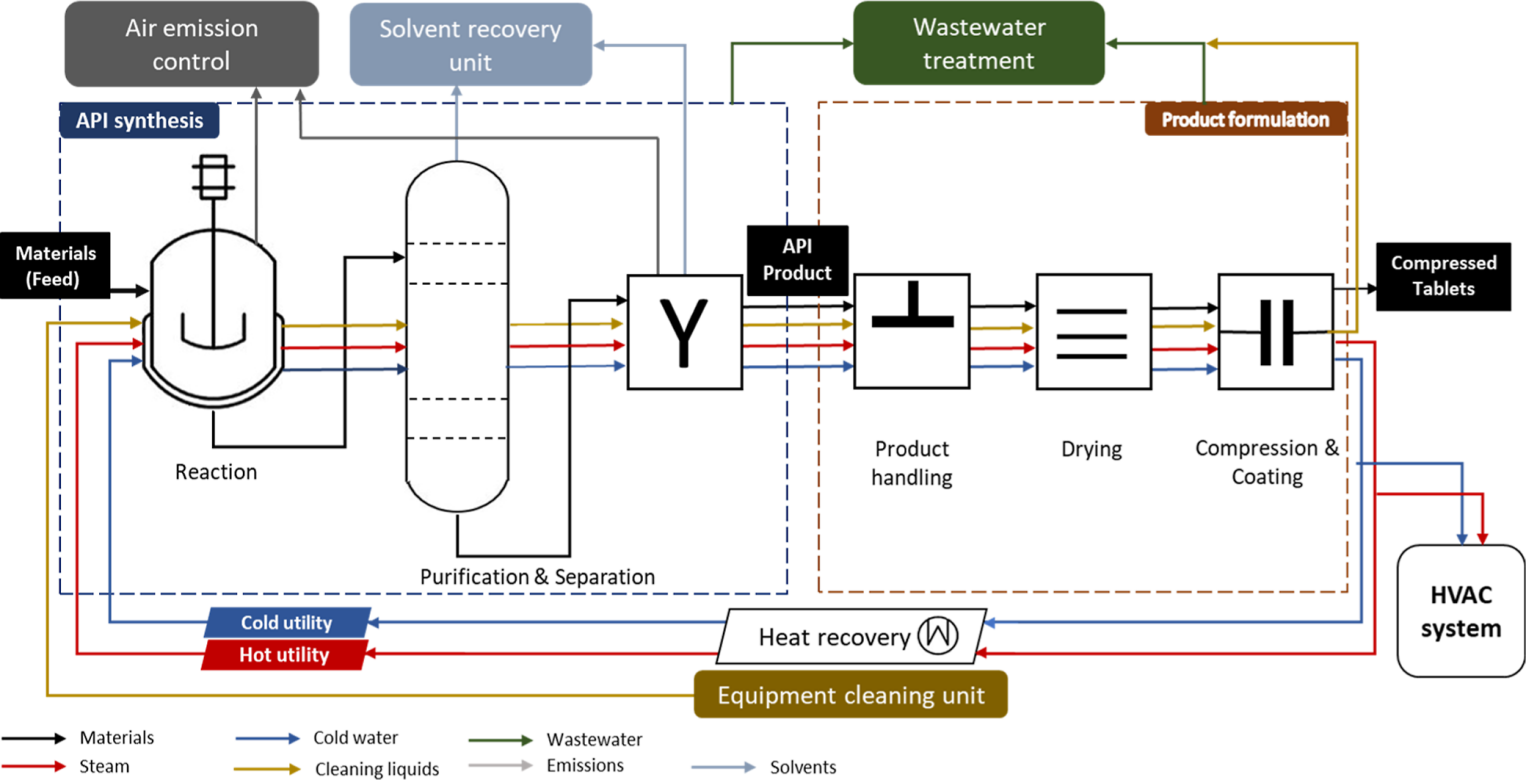
A generalized process flow diagram (PFD) of pharmaceutical production

**Table 1:** Upscaled Laboratory Processes to Industrial-Scale Unit Operations

production stage	laboratory scale step	scaled-up unit operation (UOs)
API synthesis	reaction under heating/cooling	batch reaction in a stirrer batch reactor
	low shear mixing/dissolving (magnetic stirrer, propeller, anchor)^a^	in-tank stirring
	crystallization	batch crystallization in a stirrer batch reactor
	high shear mixing/homogenizing (rotor-stator or high pressure)^b^	rotor-stator type homogenization
	pestling in mortar, grinding/milling, other particle size reduction	grinding
	filtration, sieving, centrifugation/cyclonic separation, other solid–liquid separation	filtration/centrifugation
	liquid–liquid extraction (separatory funnel)	mixer–settlers
	distillation (rotary evaporation)	distillation
	vacuum drying, drying & rotary evaporation	(oven) drying/vaporization
	(manual) transferring of liquids	pumping
synthesis-related units not included in the lab protocols (auxiliary units)	heating, ventilation & A/C (HVAC)-Air Handling Unit (AHU)	
	wastewater treatment	
	heat recovery	
	air emission control	
	equipment cleaning	
	solvent recovery-distillation	
tablet formulation	blending and mixing	tumbling
	milling (Fitz mills, colloid mills, swing hammer mill)	cone milling
	wet granulation	high shear granulation
	dry granulation	roller compaction
	Drying	fluidized bed drying
	tablet compression	rotary tablet pressing

a Refers to operations involving magnetic stirrers, propellers, or anchor-type agitators suitable for low-viscosity liquids or soluble powders.

b Refers to rotor–stator or high-pressure devices used when fine droplet or particle sizes are required, provided the formulation is not shear-sensitive.

**Table 2:** Equations Used to Calculate Energy Usage of Unit Operations

	unit operation	recommended approach	simplified approach	comments
API synthesis	reaction^a^	*Q*_R_ = *Q*_Heat/Cool_ + ∑_*i*=1_^*x*^ *S* + *Q*_Loss_ (1)	*Q*_R_ = *U*·*A*·(*T*_r,avg_ – *T*_o_)·*t*_h_ + *K*·*A*·(*T*_r_ – *T*_o_)·*t*_r_ (1A)	employed multipurpose batch reactor designs for two reactor types and five scales each. Simplified method can be used when mass ratios and specific heat capacities for complex mixtures are unavailable
		*Q*_Heat/Cool_ = (*m*_rx_·*C*_p,rx_ + *m*_a_·*C*_p,a_)·(*T*_r_ – *T*_o_) (2)	$T_{r,avg}=\frac{\left(T_{j}-T_{o}\right)+\left(T_{j}-T_{r}\right)}{2}$ (2A)	
		$Q_{loss}=a\cdot {\left(Q_{Heat/Cool}\right)}^{b}\pm k\cdot A\cdot \left(T_{r}-T_{o}\right)\cdot t_{r}$ (3)	$t_{h}=\frac{\left(T_{r}-T_{o}\right)}{H_{r}\cdot 60}$ (3A)	
	crystallization^a^	$Q_{C,col}=\frac{\left(m_{cx}\cdot C_{p,cx}+m_{a}\cdot C_{p,a}\right)\cdot \left(T_{c}-T_{o}\right)+m_{c}\cdot {\Delta H}_{c}}{\eta_{e}}$ (4)	$Q_{C}=\frac{U\cdot A\cdot \left(T_{c,avg}-T_{o}\right)\cdot t_{h}+Q_{LH}}{\eta_{e}}$ (4A)	Employed same multipurpose batch reactor designs. Consider mainly cooling and evaporative crystallization. Latent heat energy varies based on the type of crystallization
		$Q_{C,evp}=\frac{\left(m_{cx}\cdot C_{p,cx}+m_{a}\cdot C_{p,a}\right)\cdot \left(T_{boil}-T_{o}\right)+m_{s}\cdot {\Delta H}_{vap}}{\eta_{e}}$ (5)	*Q*_LH_ = *m*_c_·Δ*H*_c_ or *Q*_LH_ = *m*_s_·Δ*H*_vap_ (5A)	
	distillation^a^	$Q_{dist}=\frac{Q_{Heat}+m_{s}\cdot {\Delta H}_{vap}\cdot \left(1.2\cdot R_{\min}+1\right)}{\eta_{Loss}}$ (6)	1.53 kg of steam per kg of solvent evaporated	A simplified design of multipurpose batch reactor with the addition of distillation column. Underwood equation to determine reflux ratio *R*_min_. Simplified method: Literature-based empirical values for batch distillation^19^
		*Q*_Heat_=(*m*_dx_·*C*_p,dx_ + *m*_a_·*C*_p,a_)·(*T*_boil_ – *T*_o_) (7)		
		$Q_{cond}=\frac{m_{s}\cdot {\Delta H}_{cond}\cdot \left(1.2\cdot R_{\min}+1\right)}{\eta_{cond}}$ (8)	0.027 kg of cooling water per kg of solvent evaporated	
	drying^a^	*Q*_Dry_ = *Q*_Heat_ + *m*_liq_·Δ*H*_vap_ + *Q*_loss_+γ·*P*_N_·*t*_d_ (9)	$Q_{Dry}=\frac{m_{liq}\cdot C_{p,liq}\cdot \left(T_{boil}-T_{o}\right)+m_{liq}\cdot {\Delta H}_{vap}}{\eta_{loss}}$ (6A)	Two equipment designs to represent specificity in API and basic bulk chemical production. Assumed heat transfer rates in the industrial process remains constant and similar to the lab-scale setup
		*Q*_Heat_ = (*m*_liq_·*C*_p,liq_ + *m*_a_·*C*_p,a_)·(*T*_boil_ – *T*_o_) (10)		
		*Q*_loss_ = *K*·*A*·(*T*_boil_ – *T*_o_)·*t*_d_ (11)	$t_{d}=\frac{m_{liq}}{ER}$ (7A)	Simplified methods in the absence of drying time (*t*_d_)
		$t_{d}={0.6t}_{lab}\cdot \frac{m_{liq}}{m_{lab}}$ (12)		
	stirring	$E_{stir}=\frac{N_{p}\cdot \rho_{mix}\cdot N^{3}\cdot D^{5}\cdot t_{r}}{\eta_{stir}}$ (13)	*E*_stir_ = *P*_N_·*t*_r_ (8A)	equation from Piccinno et al.^11^ Simplified method: use literature based nominal power (*P*_N_) in kW^20^
	homogenization	refer eq 13	NA	Only few parameters were adapted in eq 13
	filtration centrifugation	1 and 10 kWh per ton of dry material	NA	Equations from Piccinno et al.^11^
	grinding	8–16 kW h/ton of grinded material	NA	
	pumping	$E_{pump}=55\frac{J}{kg}\cdot m$ (14)	NA	
tablet formulation	wet granulation^a^	*E*_wg_ = *P*_wg_·0.5*t*_wg_ (15)		Simplified the variation of literature based power consumption (*P*_wg_)with quantity of binder added with time
	dry granulation	$E_{dg}=0.131\frac{kWh}{kg}{\cdot m}_{g}$ (16)	NA	Derived from the literature based power rating per unit mass (kg) as rough estimate
	fluidized bed drying^a^	*Q*_fb_ = *C*_p,air_·(*T*_dry_ – *T*_o_)·α·ρ_air_·*t*_fb_+3600*P*_comp_·*t*_fb_ + *Q*_loss_ (17), *Q*_loss_ = *K*_wa_·*A*_L_·(*T*_dry_ – *T*_a_) (18)	*Q*_fb_ = Δ*H* × (*C*_i_ – *C*_f_) (9A)	Equipment design specifications were based on empirical data from industrial case studies.^21,22^ Simplified method: if drying time is not available, but initial and final moisture contents (*C*_i_,*C*_f_) are given
		$t_{fb}={0.6t}_{lab}\cdot \frac{m_{liq}}{m_{lab}}$ (19)		
	milling	*E*_mil_ = *P*_mil_·0.5*t*_mil_ (20)	NA	Assumed a motor efficiency of 75% and a typical operating load fraction of 75%
	mixing & blending	*E*_mixing_ = *P*_B_·*t*_mixing_ (21)	NA	Simplified the variation of literature-based power consumption (*P*_B_) with mixing time (*t*_mixing_)
	tablet compression	$E_{tc}=0.045\frac{kWh}{kg}{\cdot m}_{f}$ (22)	NA	derived from the empirical power rating per unit mass (kg) as rough estimate from an industrial case study^23^
auxiliary units	solvent recovery^a^	*Q*_codi_ = 0.78*Q*_dist_ (23)	1.21 kg of steam per kg of solvent evaporated	adapted the batch distillation energy equations by considering the variation in literature based empirical or process-simulated data. Equation was modified for novel distillation technologies based on empirical data as a conservative approach. Simplified method: Literature-based empirical values for continuous distillation^19^
		$Q_{aze}=Q_{codi}\times \frac{f_{ac}}{\eta_{ac}}$ (24)		
	HVAC^a^	*Q*_AHU_ as *V*_natural gas_ & *E*_electricity_, *Q*_AHU_ = 602.6 kW·*t*_p_ (25)	*Q*_AHU_ as *V*_natural gas_ & *E*_electricity_, *Q*_AHU_ = 44.1 kWh per kg of product	considered the energy usage separately for natural gas and electricity utilized (*V*_naturalgas_, *E*_electricity_), Recommendation method: literature based empirical energy data as a function of the production duration of a batch (*t*_p_ in hours), Simplified method: Literature based empirical values per kg derived from industrial case studies
	equipment cleaning	*E*_clean_ = [0.2*V*·ρ_s_·*C*_p,s_·(*T*_boil,s_ – *T*_o_) + *P*_pump_·2*t*_s_] + [0.4*V*·ρ_w_·*C*_p,w_·(*T*_w_ – *T*_o_) + *P*_pump_·4*t*_w_] (26)	NA	derived from a base equation by Palabiyik et al.^24^ and adapted using recommended procedures from the literature
		$t_{s}\;\text{or}\;t_{w}=0.2\frac{V}{F.A}$ (27)		
wastewater treatment-onsite			complete LCI estimation method provided in Supporting Information 1	
air emission control			complete LCI estimation method provided in Supporting Information 1	

a Focused high energy intensive unit operations under this study.

Notation: *Q* represents the production dependent steam/coolant consumption of a batch reactor (*R*), heat loss from the system (loss), cooling crystallization (c,col), evaporative crystallization (c,evp), crystallization in general (*C*), distillation (dist), condensation (cond), drying (dry), fluidized bed drying (fb), continuous distillation (codi), and azeotropic distillation (aze) in kJ, respectively; *E* represents the electricity consumption for stirring (stir), mixing (mixing), milling (mil), tablet compression (tc), cleaning (cleaning) in kWh respectively; *m* is the masses of the reaction mass (rx), the equipment/apparatus (*a*), crystallized material (*c*), solvent evaporated (*s*), distillation mass (dx), liquid or solvent (liq), final ingredient mixture (f), and granulation ingredient mixture (g) in kg respectively; *C*_p_ represents the heat capacities of the reaction mass (rx), and the material of the vessel (*a*) in kJ/kg.K respectively; *T* represents the temperature of the reaction (r), the ambient (o), average over heating period (avg), vessel jacket (j), boiling point (boil), crystallization (c), boiling point of cleaning solvent (s), and boiling point of water (w) in K, respectively; *t* represents the time taken to heat the mixture from initial temperature to reaction temperature (h), reaction (r), drying (d), fluidized bed drying (fb), solvent cleaning (s), and water cleaning (w) in seconds, respectively. η is the efficiencies of the equipment (*e*), due to losses (loss), stirring (stir), specific to distillation technology (ac); Δ*H* is the enthalpy of vaporization (vap) and crystallization (c) in kJ/kg; *P* is the motor rating of the compressor (comp), milling (mil), wet granulation (wg), and wet granulation (dg) in kW; *A*_L_ and *A* are the heat transfer area of the equipment in m^2^; *U* and *K*_wa_ are overall heat transfer coefficient in kW/m^2^·K for relevant equipment; density of the reaction mixture (ρ_mix_), density of cleaning solvent (ρ_s_), density of water (ρ_w_), and density of air­(ρ_air_)­in kg/m^3^; *a*, *b*, and *k* are parameters of the thermal losses model; correction factor specific to azeotropic mixtures (*f*_ac_); nominal power of the equipment (*P*_N_); S refers to additional terms that might be applied in special cases, such as high-pressure reactions and highly endothermic or exothermic reactions; loss coefficient (*K*) in kW/m^2^; heating and cooling rates (*H*_r_) in K/min specific to different reactor sizes; minimum reflux ratio (*R*_min_); fraction of nominal power consumed by the equipment (γ); evaporation rate (ER) in in kg/s; *N*_p_ is power number, impeller diameter given (*D*) in m; rotational speed (*N*) in 1/s; inlet air flux (α) in m^3^/h; typical flow rates for equipment cleaning (*F*) in l/min.

### Generalized PFD of Pharmaceutical Production

A generalized PFD of a typical pharmaceutical production plant was developed to assist LCA practitioners in the upscaling laboratory-scale processes to UOs and building a product-specific industrial PFD. The first step is the API synthesis. This is typically carried out as a liquid-phase batch process, often conducted in a jacketed batch reactor under stirring, with utilities such as steam and cooling water. Stepwise separation includes filtration, distillation, drying, and crystallization, which are then employed to purify or separate the final API from the rest of the mixture.

In the second production step, APIs are formulated into compressed solid tablets suitable for ingestion and absorption by the human body. Tablets are the most frequently used product type.^25^ Tablet formulation begins with handling the API and excipients, that is, the inactive substances that aid processing, stability, or delivery of the API. Depending on the process, the API–excipient blend may be processed through techniques such as wet granulation, which involves mixing the blend with liquid binders to form granules, followed by drying to achieve the desired consistency and stability for further processing. The blended granules are then compressed into tablets using a tablet press.

Onsite management of the outputs (or waste) from production steps and other auxiliary unit operations were incorporated into the generalized PFD. This includes dedicated waste management processes such as solvent-based equipment cleaning and distillation units for solvent recovery, following industrial standards and relevant literature.

### Energy Estimation

Step 3 of the procedure involves estimating the energy usage using PDCs (Table 2). It details how PDCs are derived and applied under different operational conditions and data availability. To illustrate, the reaction unit operation is presented as an example in the text box below with practical guidelines, while those for all other UOs are provided in Supporting Information 1 (Sections 4–6). Users can adapt the PDCs to their specific case, for example, by considering variations in operational conditions such as pressure and other parameter assumptions.

Each UO and its corresponding PDCs are detailed in the following format:

- A brief description of the unit operation and the associated equipment.
- A step-by-step procedure to derive relevant PDCs, including the use of empirical energy usage values from the literature where applicable.
- Recommended PDCs, with guidance on parameter settings:
  - Based on user input, taking into account the standard design parameters of different equipment types and scales.
  - Adjusting for specific operational conditions, such as varying pressures and crystallization types.
- Simplified alternative approach for cases where data are incomplete, ensuring that energy estimates can still be achieved.

For parametrization of PDCs, equipment design parameters such as standard operational scales of each unit operation are suggested and relevant data provided (see Supporting Information 1). Key sources for these standard equipment data include chemical engineering books, equipment manufacturing catalogues, and scientific literature.^26–28^

Auxiliary units, such as treatment systems not typically included in laboratory setups, lack specific operational data in lab protocols for scaling up to the industrial level. Therefore, generalized PDCs were developed from industrial case studies on auxiliary operations documented in the open literature. Energy estimates can then be calculated when data such as production mass and time are known for the API synthesis and product formulation stages.

Besides estimating energy usage, the new method also accounts for newly added unit operations, such as solvents used for equipment cleaning and excipients used in product formulation. The spreadsheet in Supporting Information 2 supports the practical implementation of the PDCs.

### Example on How to Derive a PDC: Reaction

Batch reactions are the foundation of chemical and pharmaceutical production, involving the controlled reaction of substances within a contained vessel. To estimate energy usage, a generalized system of a multipurpose batch reactor was taken as a model, incorporating a utility system including heating and cooling (Figure 3).

**Figure 3:**
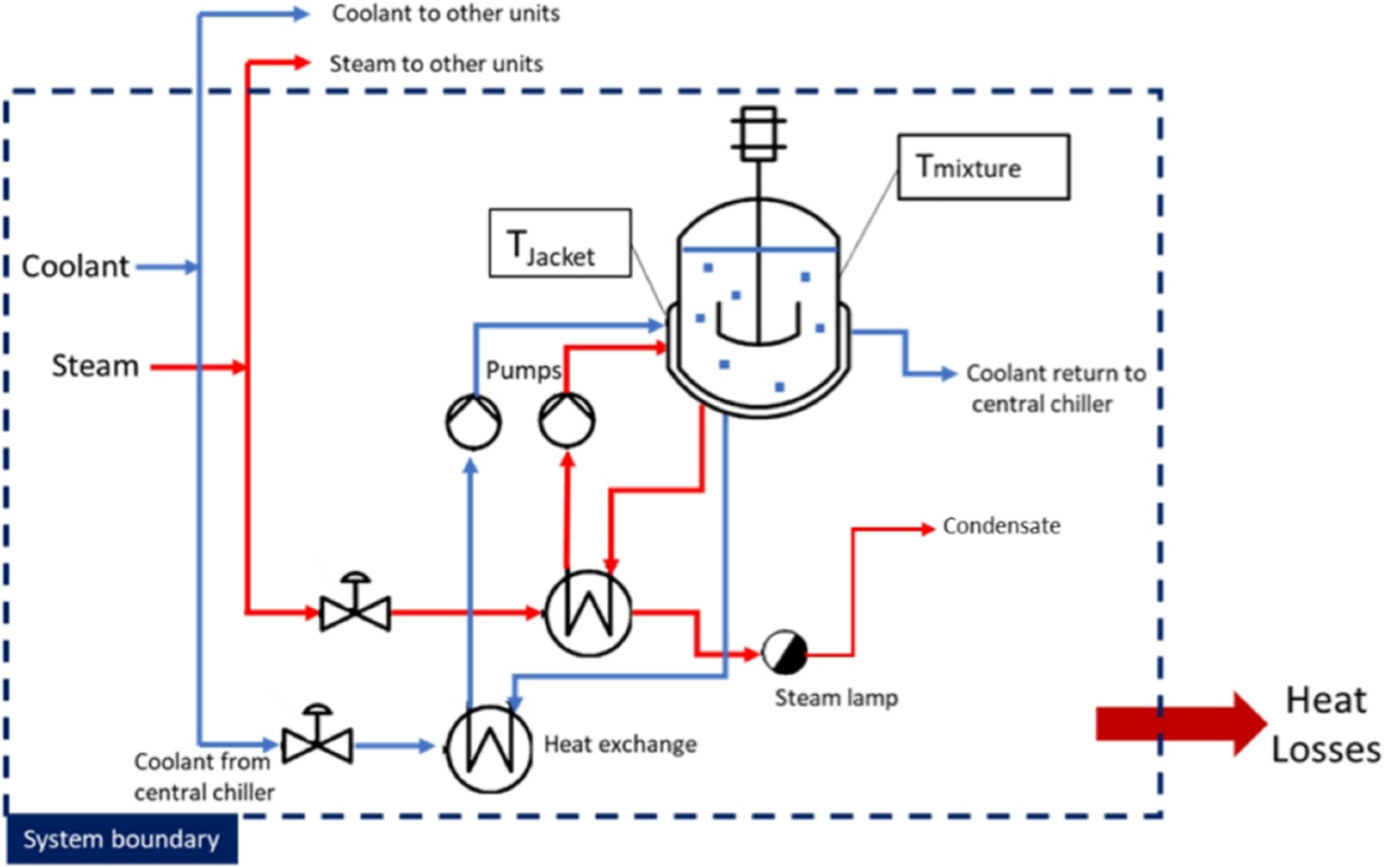
Generic batch reactor system for modeling.

The PDCs for estimating the energy usage of the reactor (*Q*_R_) were derived based on a generic model described by Bieler et al.^29^ (see Supporting Information 1: eq S2 in Section 4). This model considers the heat required to raise or lower the temperature of the mixture and the vessel, along with heat losses from the vessel, assuming pseudo steady state conditions. The model was simplified by removing parameters with negligible effects, such as dissipated energy from the stirrer (*Q*_Diss_) and heat from evaporated solvents (*m*_evap,t_ × Δ*H*_vap_) in the headspace, accounting for less than 1.5% of the total energy usage of the batch reactor.^29,30^ Empirical data from various sources, including reactor manufacturing catalogues, were used to refine the PDCs for reactor design parameters, such as the average weight of the equipment (*m*_a_) and surface heating area (*A*). An additional energy component (S) was introduced to account for the increased energy demand of specific cases, such as high-pressure and highly endothermic/exothermic reaction conditions.

Furthermore, heat loss (*Q*_loss_) from the system was captured by introducing heat loss parameters (*a*, *b* & *k*), which were derived by regression analysis of empirical values from the literature (Supporting Information 1, Table S5). For stainless-steel reactors (steam), the regression for *k* showed a strong linear fit (high *R* ^2^) and was retained as a scaling relationship (Supporting Information 1: eq S5). In contrast, the regressions for all other parameters yielded weak fits (low *R* ^2^); in these cases, averaged parameter values across scales were applied to improve the robustness and ensure consistent application. The resulting parameters are valid within the reactor size ranges reported in Table S5 of Supporting Information 1 and should be regarded as approximate scaling factors.

Ultimately, this resulted in the following equations

1 $$Q_{R}=Q_{Heat/Cool}+\sum_{i=1}^{x}S+Q_{loss}$$

where

2 $$Q_{Heat/Cool}=\left(m_{rx}\cdot C_{p,rx}+m_{a}\cdot C_{p,a}\right)\cdot \left(T_{r}-T_{o}\right)$$

3 $$Q_{loss}=a\cdot {\left(Q_{Heat/Cool}\right)}^{b}\pm k\cdot A\cdot \left(T_{r}-T_{o}\right)\cdot t_{r}$$

where *Q*_R_ is the production dependent steam/coolant consumption of a batch reactor in kJ; *m* is the masses of the reaction mass (rx), the equipment/apparatus (*a*), in kg respectively; *C*_p_ represents the heat capacities of the reaction mass (rx), the material of the vessel (a) in kJ/kg.K, respectively; *T* represents the temperature of the reaction (r), the ambient temperature (o), in K respectively.

To apply these equations, guidelines are provided (see Supporting Information 1 for details) for setting parameters such as the average heat capacity of the reaction mixture (*C*_p,rx_) across different temperature ranges and mass ratios. Apart from these process-specific data, suggested standard reactor specifications for glass lined or stainless-steel reactors at different scales (see Supporting Information 1 and Table S5) should be applied to the PDCs based on user requirements. To reflect practical energy-saving strategies in industrial settings, a moderate level of heat integration was assumed, applying a 20% heat recovery factor to the total calculated thermal energy.^31^

In cases in which specific data for complex mixtures are unavailable (e.g., mass ratios or heat capacities), the following simplified equation can be applied to obtain a conservative estimate of energy usage:

1A $$Q_{R}=U\cdot A\cdot \left(T_{r,avg}-T_{o}\right)\cdot t_{h}+K\cdot A\cdot \left(T_{r}-T_{o}\right)\cdot t_{r}$$

Since most of these parameters are typically unknown, conservative assumptions were made and values were assigned based on appropriate industrial scale specifications from the literature (see Supporting Information 1 for details).

### Case Study

To illustrate the energy estimation method outlined above, a cradle-to-gate LCA was conducted for six pharmaceutical products, focusing on their carbon footprint. Diclofenac, Ibuprofen, Lidocaine, Morphine, Dexmedetomidine, and Paracetamol were selected to illustrate the broad applicability of our method across pharmaceutical processes. Carbon footprint was chosen for its strong correlation with energy consumption as it is driven by GHG emissions from energy generation. Processes such as the extraction, production, and use of raw materials, including upstream chemicals, can also substantially contribute to the carbon footprint of pharmaceutical products.

The LCA was performed using SimaPro v9.4, employing the ReCiPe Midpoint (H) impact assessment method.^32^ For APIs typically applied in the fluid form, that is, Morphine, Dexmedetomidine, and Lidocaine, the functional unit (FU) was defined as the production of 1 kg of API. For APIs typically applied as tablets, that is, Diclofenac, Paracetamol, and Ibuprofen, the FU was defined as the production of a tablet quantity containing 1 kg of API.

For the LCI, energy usage for API synthesis and tablet formulation was estimated using the stepwise procedure outlined above, applying the recommended approach when the required data were available. A literature search was conducted using open-access patent databases and peer-reviewed articles to identify study protocols that can be applied to the current industrial practice. When multiple synthesis routes were identified, the final selection was made using the criteria defined in the methods, informed by the consultation of chemistry experts. A summary of the available routes per API and the selection rationale is provided in Section 8.1 of the Supporting Information 1. When the selection of a unique realistic synthesis route is not feasible based on the defined criteria, the method can be applied to compare alternative synthesis routes for the same API by conducting route-specific UO mapping and upscaling. This will help us to check for sensitivity to the route selection step. Material consumption, including reactants, solvents, and yields, was scaled up linearly from laboratory protocols based on stoichiometric calculations. Inventory data for the production of upstream chemicals and background processes, such as energy utility supply, were obtained from the ecoinvent database v.3.10.^33^

Upstream chemicals unavailable in ecoinvent were modeled step by step using our method, which constructs their synthesis routes from study protocols. If further precursors or chemicals required for these routes were also not covered in the ecoinvent database, they were modeled using our method until all required inputs were available (see Supporting Information 1: Figures S17–S22). For example, in the diclofenac route, dichlorophenol was modeled as a precursor since it is not in ecoinvent. To construct its synthesis, tetrachlorocyclohexanone also had to be modeled. Solvent recovery was considered as an auxiliary operation for these modeled chemicals since extensive quantities of solvents are typically consumed in upstream chemical production.

Our method includes estimations for utilities. For steam, the ecoinvent unit data set of ‘steam production in the chemical industry’ was taken, which includes energy losses and water use in upstream processes. This steam process was primarily used for solvent recovery operations, while other heating needs were met using the ‘steam production, as energy carrier, in chemical industry’ data set, representing European chemical industry standards. For process cooling, specific data sets are not available in the ecoinvent database. Therefore, an LCI data set for cooling water and refrigeration was developed based on the work of Jimenez-Gonzalez.^34^ For electricity, the medium voltage, European residual mix data set was employed, representing the average electricity generation mix in Europe and accounting for the different sources of energy production utilized in the region. This study focuses on foreground scaling; prospective background data (e.g., future grid mixes) that were not included were considered out of scope.

We compared our results with those obtained after applying the existing method of Parvatker et al.^35^ This method was then also used to model the production of upstream chemicals. Moreover, carbon footprints related to the total energy usage of foreground processes were estimated by using both methods and compared to a reference carbon footprint to determine how our method aligns with industry data. To establish this reference, the carbon footprint of the median total energy usage per kilogram of product was derived from data collected across 30 industrial sites (see Supporting Information 1: Section 3) in Italy and the United States.^36,37^

### Sensitivity Analysis

To evaluate the influence of modeling assumptions and data heterogeneity, sensitivity analyses were performed. The first varied the allocation of the AHU energy. The second reflects the uncertainty in the industrial reference value, applying the 10th and 90th percentiles of the data set as alternative reference values. The third varied the solvent recovery rate, with 75% as the base scenario and alternative values of 50% and 90%.

In the base scenario, the median total energy consumption per kilogram of product was derived from the data set of 68 pharmaceutical facilities,^37^ with 10.5% of total energy per line/product attributed to AHU, assuming two production lines per site. In the sensitivity analysis, the AHU share was varied according to different assumptions about the number of production lines. This share was then applied to total energy consumption values taken from the 10th (lower), median, and 90th (upper) percentiles of the same data set, resulting in per-product allocations of 15% (2 lines), 10.5% (2 lines), 4.2% (5 lines), and 2.1% (10 lines). The detailed selection of these values is provided in Section 7 of the Supporting Information 1.

## Results

### Energy Usage

Figure 4 presents the energy usage estimates in terms of thermal energy and electricity consumption for the production of the six selected pharmaceuticals by using the new and existing methods. Overall, the new method shows substantial differences compared to the existing method. These differences even exceed 102% for Lidocaine, Diclofenac, Paracetamol, and Ibuprofen for both thermal energy and electricity consumption. The largest differences are observed in the auxiliary operations (black bars in Figure 4). In contrast, other production stages use relatively little thermal energy and electricity. Among these, API synthesis generally consumes more energy than product formulation.

**Figure 4:**
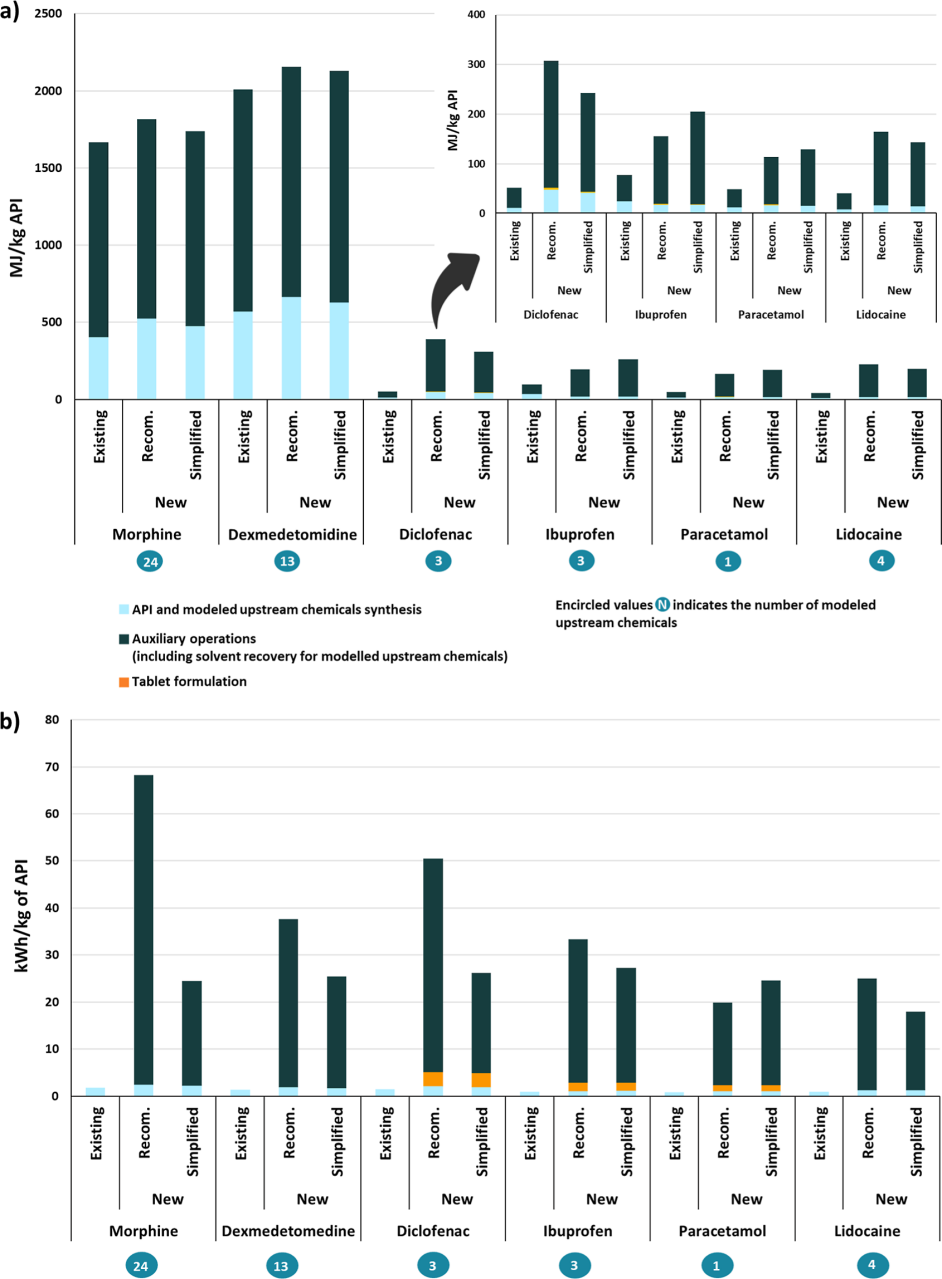
Comparison of energy usage estimates for the production of 1 kg of the six selected pharmaceutical products using the new and existing methods. (a) Thermal energy usage. (b) Electricity consumption.

Morphine and Dexmedetomidine show thermal energy consumption exceeding 1400 MJ/kg of API (Figure 4a), while all other APIs consume less than 310 MJ/kg. Electricity consumption varies less among the APIs, as shown in Figure 4b.

The new method offers two alternatives, that is, using recommended approaches or using simplified approaches in case data and/or time are lacking. Use of the simplified approach resulted in thermal energy estimates that ranged from 20% lower to 31% higher than the recommended approach (Figure 4a). Electricity consumption showed even larger differences, exceeding 50% for most APIs and reaching 66% for Dexmedetomidine (Figure 4b), mainly due to differences in auxiliary operation’s energy estimates.

### Carbon Footprint

Figure 5 compares the carbon footprint of the six selected pharmaceutical products based on estimates from our new method and the existing method. The new estimation method results in higher carbon footprints primarily due to the energy usage associated with newly added UOs. Across APIs, carbon footprints are 3% to 49% higher, with the largest differences observed for Lidocaine, Ibuprofen, Paracetamol, and Diclofenac. Morphine and Dexmedetomidine show smaller differences (less than 5%) due to their high total carbon footprint, which is mainly driven by GHG emissions from raw materials rather than energy use. The total carbon footprint of selected APIs ranges from 25.4 kg CO_2_ eq per kg of Lidocaine to 2965 kg CO_2_ eq per kg of Dexmedetomidine.

**Figure 5:**
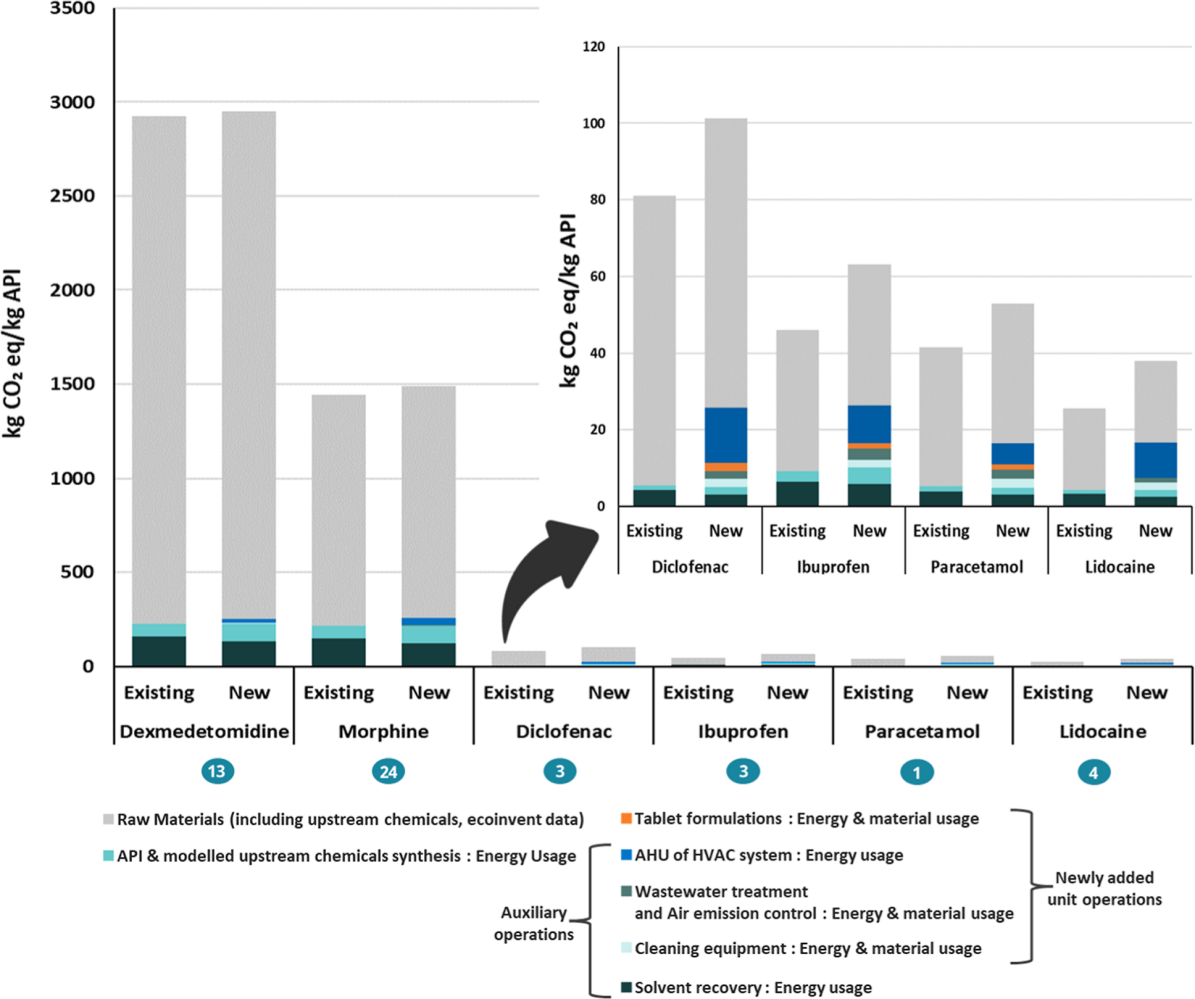
Comparison of the carbon footprint per kilogram of API produced for six pharmaceutical products, based on estimates from the new and the existing method.

The largest increase in the carbon footprint between the two methods is mostly attributed to the energy consumption of the Air Handling Unit (AHU) in the HVAC system. Other newly added unit operations such as wastewater treatment, air emission control, and cleaning equipment contribute 6–10% to the carbon footprint of Diclofenac, Ibuprofen, Paracetamol, and Lidocaine, whereas these operations contribute less than 1% for Morphine and Dexmedetomidine.

Raw materials for API synthesis, including upstream chemicals, contribute most to the total carbon footprint in the new method (gray bars in Figure 5). The AHU is the second-largest contributor, accounting for 1% to 25% of the carbon footprint, with the highest impact observed for APIs with fewer upstream chemicals. The other unit operations taken together, for example, reaction processes, solvent recovery and product formulation, contribute 12–36% to the carbon footprint.

Figure 6 shows the carbon footprint related to the estimated total energy usage in foreground processes for the six selected APIs using both methods, compared against the industrial reference value for an average pharmaceutical. The results reveal that the new method aligns more closely with the industrial reference value of 32.2 kg of CO_2_-eq, derived from energy usage data collected across 30 industrial sites in Italy and the United States. For these APIs, the carbon footprint ranges from −64% to +52% of the reference value, while the existing method shows much larger deviations, that is, −93% to −81%.

**Figure 6:**
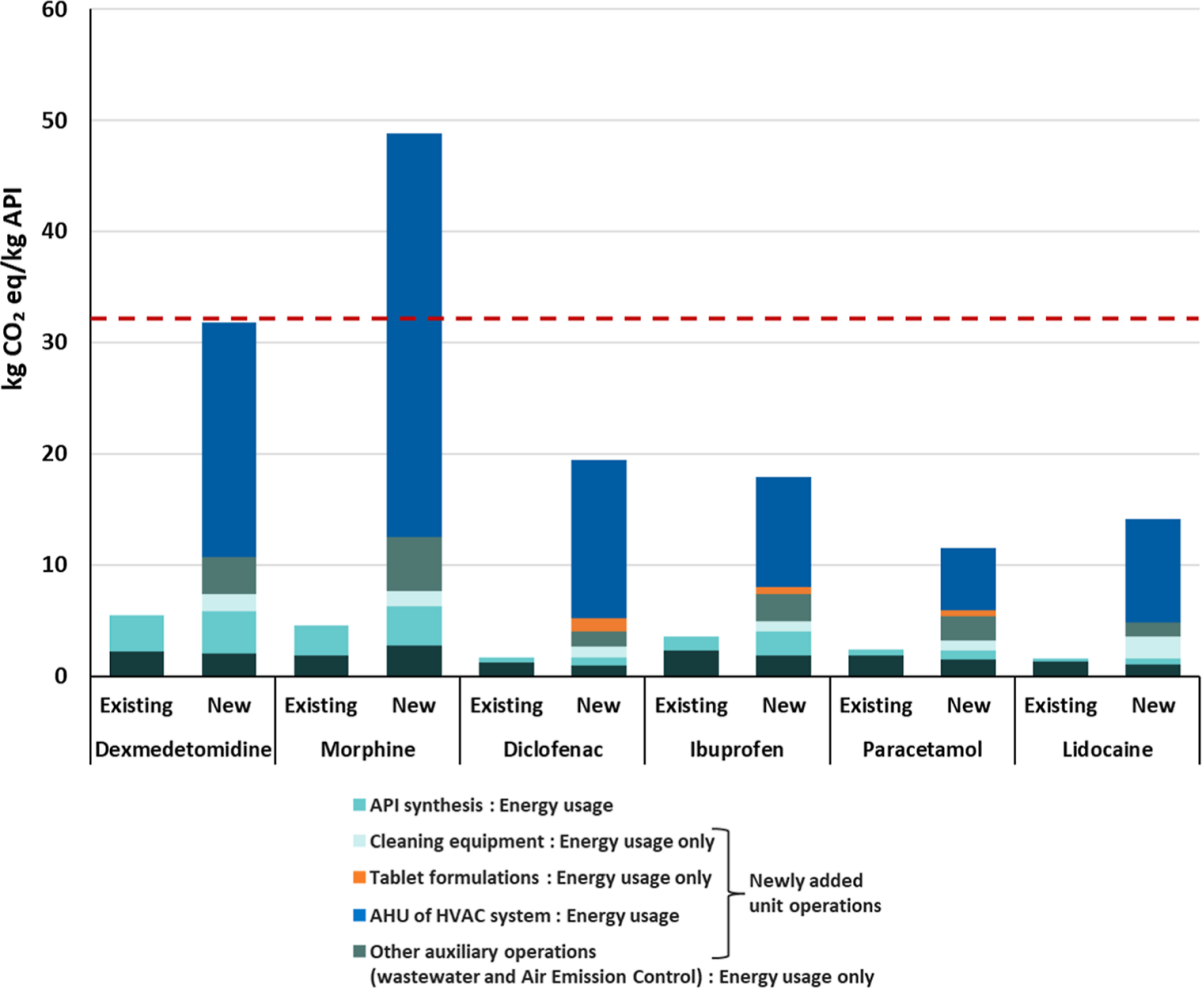
Carbon footprint for production of 1 kg of six selected APIs based on foreground energy estimates from the new and existing methods, compared to a reference carbon footprint derived from empirical industrial energy data (dashed line).

### Sensitivity Analysis

#### Sensitivity to AHU Allocation Assumptions

Variations in solvent recovery rates changed the carbon footprint by less than 7% compared with the base scenario (75% recovery), and the detailed results are presented in Supporting Information 1. Figure 7 illustrates the sensitivity of carbon footprints to AHU allocation assumptions for Ibuprofen and Diclofenac, representing the largest decreases and increases relative to the base case among the six APIs. The largest decrease (15–22%) occurs under the 10th-percentile scenarios with five and ten production lines, while the largest increase (26–42%) occurs under the 90th-percentile scenario with two lines. Despite these variations, AHU remained the second-largest contributor in most cases. Results for the other APIs are provided in the Supporting Information 1 (Figure S8).

**Figure 7:**
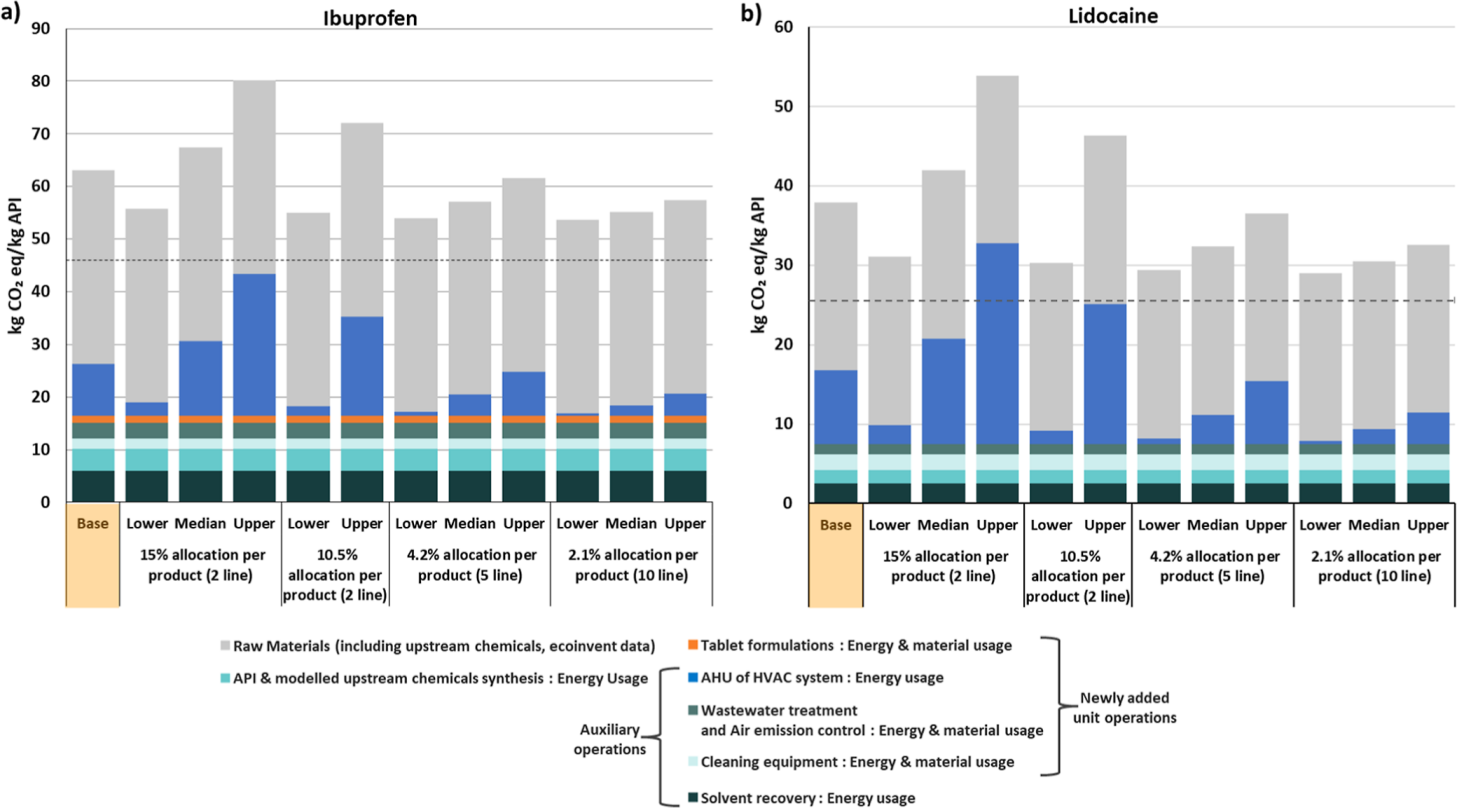
Carbon footprint per kilogram of Ibuprofen and Diclofenac under different HVAC allocation scenarios. Results are based on the 10th, mean, and 90th percentiles of the 68-facility data set, with HVAC energy shares distributed across two, five, or ten production lines. The dotted line shows the carbon footprint derived with the existing method.^12^ (a) Ibuprofen. (b) Lidocaine.

#### Sensitivity to Reference Benchmark Values

The new method with AHU allocation scenarios as well as the existing method were compared against the median, 10th percentile and 90th percentile of the reference value distribution (see methods). Figure 8 shows the two allocation extremes: a two-line case (15% HVAC per product) and a 10-line case (2.1% HVAC per product). For the 10th-percentile reference, the existing method predicts 46 to 82% lower carbon footprint. The new method is closer to this reference, showing a maximum decrease of 44% under the assumption of 10 production lines where the HVAC share is the smallest (Figure 8b). The existing method predicts more than 95% lower carbon footprint compared to the 90th-percentile reference, while the new method remains within +27% to −76% under the assumption of 2 production lines (Figure 8a). Results for the other allocation scenarios are provided in Supporting Information 1 (Figure S9).

**Figure 8:**
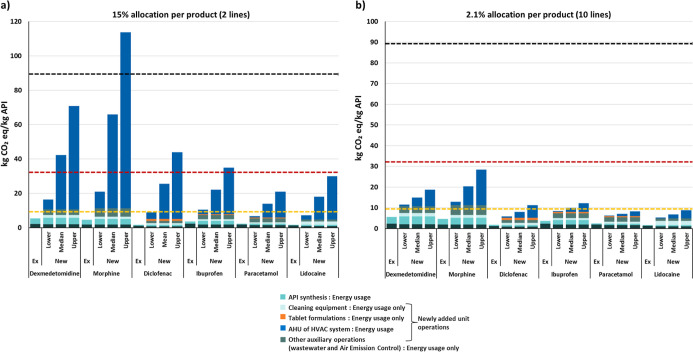
Comparison of carbon footprints per kilogram of API from the existing and new methods against percentile-based industrial reference values. Results are shown for the two allocation extremes: (a) 2-line allocation (15% AHU per product) and (b) 10-line allocation (2.1% AHU per product). The black dotted line indicates the upper percentile (90th), the red dot line indicates the median, and the yellow line indicates the lower percentile (10th).

## Discussion

The higher energy estimates of the new method are mainly due to the inclusion of the AHU of the HVAC system. Prior studies have similarly highlighted HVAC as one of the most energy-intensive operations in pharmaceutical production, with Gamiz et al.^38^ reporting that HVAC dominates electricity consumption in facilities.

For API synthesis, energy profiles are influenced by the complexity of upstream synthesis, number of modeled upstream chemical productions, and energy usage of HVAC. Thermal energy usage tends to increase with the complexity of the synthesis, as more upstream chemical production routes must be modeled, requiring additional reaction, purification, and solvent recovery steps. This pattern is reflected by Morphine and Dexmedetomidine, which show higher thermal consumption due to their complex precursor production routes. In contrast, electricity usage does not follow this trend, as it is largely dominated by HVAC energy demand rather than chemical processing.

The variation in estimates between the simplified and recommended approaches within the new method demonstrates that methodological choices can affect the results. These differences are driven largely by varying assumptions about the HVAC energy usage. The recommended approach calculates HVAC energy based on production time, allowing for more accurate allocation. In contrast, the simplified approach applies generic average values per kilogram of product, which do not capture production-specific variations.

The increase in the carbon footprint resulting from the new method highlights the contribution of previously unaccounted unit operations in pharmaceutical production. HVAC systems remain the dominant contributors to this increase. Other newly included operations contribute up to 8% to the total footprint, not only through energy consumption but also through material use. For example, tertiary wastewater treatment using UV/H_2_O_2_ advanced oxidation has been reported to consume up to 3.3–9.1 kWh m^–3^, compared to 0.8–1.2 kWh m^–3^ for secondary biological treatment.^39,40^ Additionally, solvent use for equipment cleaning and the addition of excipients during formulation contribute to the overall material-related emissions.

The difference in the carbon footprint between the new and existing methods is strongly influenced by the raw material inputs. For complex APIs such as Morphine and Dexmedetomidine, carbon footprints are already high because GHG emissions from upstream chemicals far exceed those from energy usage for API production. As a result, the additional contributions introduced by the new method have a relatively small effect on the total footprint. In contrast, relatively simple APIs such as Lidocaine, Ibuprofen, Paracetamol, and Diclofenac have lower carbon footprints, because these have less upstream chemicals. This makes the additional contributions captured by the new method more evident in the total footprint.

Although the comparison between Diclofenac, Ibuprofen, and Paracetamol was based on the functional unit of producing 1 kg of active ingredient, patient-level consumption can alter these outcomes. The defined daily dose (DDD) is notably lower for Diclofenac (0.1 g/day) than for Ibuprofen (1.2 g/day) or Paracetamol (3.0 g/day). As a result, when expressed from a patient-year perspective, Diclofenac corresponds to 3.9 kg of CO_2_, compared to 32 kg of CO_2_ for Ibuprofen and 73.7 kg of CO_2_ for Paracetamol, making Diclofenac appear environmentally favorable. However, a lower production footprint does not necessarily indicate a lower overall environmental impact. Higher ecotoxicity of Diclofenac increases risks to aquatic ecosystems,^41^ illustrating a clear trade-off between lower emissions at production and greater environmental persistence and toxicity at end-of-life.

While the new method provides a more comprehensive estimation, it still has several limitations regarding applicability and data quality. It is limited to batch processing, excluding other production methods like continuous or semibatch processing. Another limitation is that patent data alone may be insufficient to generate reliable inventories. For future studies, structured extraction modeling approaches, such as the one proposed by Spreafico et al.,^42^ could be applied to systematically retrieve and interpret inventory data from patents.

Third, the estimation of AHU energy usage is hindered by uncertainty in facility-level empirical data and industrial reference value. The sensitivity analyses show that although the absolute carbon footprint values vary substantially under different assumptions, the role of the AHU as the second-largest contributor remains consistent across most APIs. Comparison with alternative industrial reference values further demonstrates that the new method aligns more closely with industrial data than does the existing method. This analysis therefore confirms the robustness of the conclusions and highlights the importance of including AHUs in environmental assessments. For robust estimates, more empirical facility-level data are needed.

Other unit operations with high contributions to the environmental impact could be refined, as well. These include material input and output assumptions, where the linear upscaling of solvent consumption from lab quantities increases uncertainty.

## Conclusion

Our method facilitates industrial-scale LCA of production processes still in the experimental phase. By applying a prospective LCA, it evaluates potential environmental impacts when scaling beyond laboratory conditions, considering technological advancements and process evolution. These early assessments enable comparisons with established industrial alternatives, while addressing uncertainties and scaling effects. Additionally, the method helps to identify environmental hotspots and offers insights into process optimization and sustainability improvements. By addressing these factors early, it supports informed decision-making for a smoother transition to commercial-scale production.

Our study shows that energy usage can contribute over 17% of the total carbon footprint, particularly for relatively simple APIs. This underscores the relevance of our method, offering a more realistic representation of industrial energy usage and reducing the risk of underestimating environmental impacts. Expanding this method to continuous processing and refining its estimates through further studies will enhance its applicability domain, for example, to evaluate industrial-scale impacts during early pharmaceutical development within a prospective LCA context.

## Supplementary Material





## Notes

The following files are available free of charge. The Supporting Information is available free of charge at https://pubs.acs.org/doi/10.1021/acssuschemeng.5c04708.

Detailed guidelines for PDC application; process flow diagrams; selection criteria for synthesis-route selection in the case study; upstream chemical routes for LCI modeling; and sensitivity-analysis results (PDF)
Spreadsheet tool for PDC implementation and upscaling to the selected industrial batch scale (XLSX)
