# Supplementary material for: Closing Data Gaps for LCA of Pharmaceutical Production: Estimating Energy Usage by Upscaling Laboratory Data
Source: ACS Sustain Chem Eng. 2025 Nov 14;13(47):20363–76. doi: 10.1021/acssuschemeng.5c04708 (PMC12673579; doi:10.1021/acssuschemeng.5c04708)
Supplement: Supplementary file 1 [file sc5c04708_si_001.pdf]

Supporting Information 1

Closing Data Gaps for LCA of Pharmaceutical Production: Estimating Energy Usage by Upscaling Laboratory Data

Muhammed Ayaj Ansar†, Rosalie van Zelm†, Ad M.J. Ragas†*

† Department of Environmental Science, Radboud Institute for Biological and Environmental Sciences (RIBES), Radboud University, Heyendaalseweg 135, 6525 AJ Nijmegen, Netherlands

*Corresponding author email: ayaj.ansar@ru.nl

Total Pages of Supporting Information: 86
Number of Figures: 22
Number of Tables: 19

CONTENTS

Guidelines for energy estimation

Section 1: Upscaling and Mapping Unit operations S4

Section 2: Raw-Material scaling up S6

Section 3: Reference industrial values S7

Section 4: API synthesis S8

1. Reaction ... S8
2. Crystallization ... S17
3. Distillation ... S19
4. Drying ... S21
5. Stirring Energy ... S23
6. Other API synthesis operations ... S25

Section 5: Product formulation S26

7. Wet granulation ... S26
8. Dry granulation ... S28
9. Fluidized bed drying ... S28
10. Milling ... S30
11. Mixing and blending ... S30
12. Tablet compression (or tableting) ... S30

Section 6: Auxiliary operations S32

13. Solvent recovery ... S33
14. Heat Recovery ... S37
15. Air Handling Unit (AHU): HVAC ... S37
16. Equipment or Facility cleaning ... S39
17. Air emissions control ... S41

17\. Wastewater treatment ... S45

Section 7: Sensitivity analysis S54

HVAC allocation assumptions S54

Sensitivity to AHU allocation assumptions S55

Sensitivity to reference benchmark values S56

Sensitivity to Solvent recovery assumptions S57

Section 8: Case study **S59**

8.1 Identification of API Synthesis route S59

8.2 Process Flow of api synthesis S70

8.3 Upstream chemical modelling S73

References **S77**

Guidelines for energy estimation

SECTION 1: UPSCALING AND MAPPING UNIT OPERATIONS

To illustrate how the stepwise mapping procedure is applied, we present a worked example based on laboratory-scale descriptions of ibuprofen synthesis and tablet formulation from selected protocols/patents.[1,2] Table S1 lists each laboratory step and assigns it to the most representative industrial unit operation (UO) defined in Table 1 of the main text.

Table S1. Most representative industrial level UO in terms of equipment and function to laboratory API synthesis and tablet formulation of ibuprofen.

	Production stage	Laboratory-scale step	Scaled-up Unit Operation
**API synthesis**	**Stage 1: 4-isobutylacetophenone (IBAP) formulation**	Charge isobutylbenzene & acetic anhydride to Hastelloy C autoclave; cool to 5 °C; evacuate	Batch reaction in a stirrer batch reactor
		Add anhydrous HF; heat to 80 °C for 3 h under stirring	Batch reaction in a stirrer batch reactor
		Vent HF via caustic scrubber with N_2 sparge	Air emission control
		Extract organics with ethyl acetate	Mixer–settlers
		The ethyl acetate solution was dried over anhydrous magnesium sulfate	Mixer–settlers
		Concentrate organics under reduced pressure	Distillation
	**Stage 2: Hydrogenation (IBAP →IBPE**	Charge IBAP and Raney nickel; purge with N_2 then H_2; pressurise with H_2 and hold at 70 °C for 3h	Batch reaction in a stirrer batch reactor
		Filter off Raney nickel (retain wet)	Filtration/centrifugation
	**Stage 3: Carbonylation (IBPE → Ibuprofen)**	Charge IBPE, $PdCl_2(PPh_3)_2$, aqueous HCl, benzene; purge with N_2 and CO; pressured to 800 psig with CO and heated to 125–129 °C for 6 h with stirring.	Batch reaction in a stirrer batch reactor
		Vent CO after reaction	Air emission control
		The organic layer was separated from the aqueous layer, washed with ethyl acetate.	Mixer–settlers

		Organic fractions were dried over anhydrous sodium sulfate	Mixer–settlers
		Concentrated under reduced pressure to give a greenish oily product.	Distillation
	**Throughout all stages**	Routine in-tank dissolving / low-shear agitation	In-tank stirring
		(If needed for dispersions) High-shear mixing	Rotor-stator type homogenization
		Transfer intermediates/solutions between steps<br>*applies for tablet formulation	Pumping
**Tablet Formulation**		Pre-mix ibuprofen with excipients (Providone and Starch)	Tumbling
		Wet granulation: add binder in portions; mix low/high speed to a satisfactory wet mass	High shear granulation
		Dry the granules in a fluid-bed dryer at 55°C<br>for 3 h.	Fluidized bed drying
		Mill through 1.25 mm screen; collect to blender	Cone milling
		Blend lubricants: sieve lubricants/glidants (aerosil, stearate); add to blender; short mix	Tumbling
		Slugging granules on rotary press (1-inch punches)	Dry granulation — Roller compaction
		Mill slugs; sieve fractions; return sized material to blender	Cone milling
		Final blend with any remaining excipients; short mix	Tumbling
		Compress tablets; control RH 45–55% at 25–27 °C; in-process checks	Rotary Tablet Pressing

Next, we integrate the mapped unit operations into a process flow diagram (PFD) (Figure 2 of the main text) to facilitate how the operations interlink to produce the final product. The process sequence includes multiple reaction and purification steps (e.g., distillation and filtration) that yield the API, followed by product handling (mixing and granulation), drying, and final tablet compression.

To show how the auxiliary systems connect to the main line in the PFD via the relevant material and utility streams, Table S2 lists the systems referenced in the worked example. These units manage

utilities, emissions, solvent recycling, cleaning, and wastewater, and are not part of the product stream.

Table S2. Linkage of auxiliary operations to process flow diagram.

Auxiliary unit	Where it connects in this example	Function in PFD
Air emission control	Reactor vents (e.g. HF, H_2, CO) and distillation overheads	Scrubbing/abatement; emissions line in legend
Solvent recovery – Distillation	From all distillation/mother-liquor and solvent streams	Recycle solvents to the solvent header
Wastewater treatment	From neutralisation/extractions and residue water from the cleaning system	Treat aqueous streams; discharge to effluent
Equipment cleaning	To/from reactors, separators, dryers, filters	CIP cycles; spent cleaning to WWT
Heat recovery	Around reboilers/condensers, tempered loops	Utility integration (hot/cold)
Heating, ventilation & A/C (HVAC) – AHU	API synthesis/formulation areas (facility-level)	Environmental control; energy accounted per your method

SECTION 2: RAW-MATERIAL SCALING UP

The mass consumption inventory is linearly upscaled to the selected industrial batch scale using a dedicated Excel tool developed for upscaling and mass balance verification in Supporting Information 2.

Table S3. Overview of methodological approaches to estimate material input and output LCI data.

Type	Inventory data	Reference	Specification/modification
Inputs	Masses of substrates, reactants, etc.	-	Upscale information from patents or other literature<br>Stoichiometric calculations
Outputs`	Production waste	-	Consists of:<br>Yield losses and by- products.<br>Outputs from cooling tower (i.e. sludge from make-up water pre-treatment).

Fugitive emissions	Following Jiménez-González and Overcash 2000.3	For gases: 0.5% of the input material<br>For liquids: 2% (if boiling point (BP) is between 20 and 60°C); 1% (if BP is between 60 and 120°C) of the input material.<br>If BP of a substance is above 120°C, no fugitive loss will be assumed.<br>Not apply for submodule cooling tower or auxiliary processes (e.g. gas scrubbing)<br>Only applied for inputs<br>Fugitive emissions consider to be further treated and emissions from treatment systems can be calculated from the approach given under this study.
Product/yield	Following Parvatker et al.5	70% over the entire stoichiometry - Different values can be preferred.<br>No differentiation between fine and bulk chemicals; if patent provides detailed information on inputs and outputs, the yield is adjusted.

SECTION 3: REFERENCE INDUSTRIAL VALUES

To benchmark our results against real-world data, we used two independent datasets that both provide production capacity information and are comparable in terms of production type (generic drug and tablet formulation):

- A subset of 14 plants from Bruni et al.[6] restricted to sites with annual production capacities between 20,000 and 540,000 kg. This subset was chosen because Table 2 of Bruni et al. reports acceptable coefficients of variation in this capacity range, making the results moderately reliable.
- A subset of 16 pharmaceutical plants from the Industrial Assessment Center (IAC) dataset, [7] selected for the same reason: these plants report production capacities and are correspond to generic drug and tablet formulation facilities, while biopharmaceutical plants were excluded.

These two subsets together represent one of the few recent empirical sources on pharmaceutical energy consumption, and they enable comparison of carbon footprint results on a consistent functional unit basis (kg API/product).

Instead of treating these as a single homogeneous dataset, we harmonised them by aligning on the functional unit (total energy per kilogram of product) and by fitting a lognormal distribution across the pooled values within the overlapping production capacity range (Table S4). The median of this distribution was taken as the baseline reference value, while the 10th and 90th percentiles were applied in sensitivity analyses. The carbon footprint resulting from these energy usages were then considered as the reference values.

Table S4. Data sources used to reference industrial energy per kg of product.

Minimum production in kg	Maximum production in kg	No. of production plants	Reference
20000	540000	14	Bruni et al.[6]
10000	200000	16	IAC 2024.[7]
Median total energy consumption: 210.2 kWh/kg			
Reference industrial value (median): 32.2 kg CO_2-eq			

This approach was chosen because both datasets cover similar types of production facilities but stem from independent sources, which increases robustness compared to relying on one dataset alone.

SECTION 4: API SYNTHESIS

1. REACTION

Batch reactions are the foundation of chemical and pharmaceutical production, involving the controlled reaction of substances within a contained vessel. To model energy usage, a generalized design of a multipurpose batch reactor was utilized, incorporating an integrated utility system of heating and cooling. The reactor comprises a vessel equipped with stirring equipment (refer Section 5).

The PDCs for estimating the energy usage of the reactor were derived based on a generic model previously investigated and applied by Bieler et al.[8] This choice was made because the Bieler model was developed and validated for multipurpose batch plants equipped with DIN standard reactors,

which are representative of the types of vessels commonly used in the fine chemical and pharmaceutical industries. This model primarily considers the heat required to raise or lower the temperature of the reactor and reaction mixture, along with heat losses from the overall system, assuming that pseudo steady state conditions are achieved. However, since this model requires comprehensive input data and does not account for heat losses from utility systems, it was refined and adapted to Equation 1 for the context of this research using methodological approaches detailed in the methods section.

$$Q_R = Q_{Heat/Cool} + \sum_{i=1}^{x} S + Q_{Loss} \tag{1}$$

$$Q_{Heat/Cool} = \left(m_{rx} \cdot C_{p,rx} + m_a \cdot C_{p,\mathrm{a}}\right) \cdot (T_r - T_o) \tag{2}$$

Where Q_R is the production dependent steam/coolant consumption of a batch reactor in kJ; m are the masses of the reaction mass (rx), the equipment/apparatus (a), in kg respectively; C_p represents the heat capacities of the reaction mass (rx), the material of the vessel (a) in kJ/kg.K, respectively; T represents the temperature of the reaction (r), the ambient temperature (o), in K respectively; Heat loss from the system (Q_{Loss}) in kJ. S refers to additional terms that might be applied in special cases, such as high-pressure reactions and highly endothermic or exothermic reactions (refer Section 1.2).

In this context, the initial temperature of the mixture is typically assumed to be the ambient temperature (T_o) at 25 °C = 298.15 K if the feed is not from a previous step at a different temperature. Additionally, under steady-state conditions, the temperature of the reactor vessel is considered to be the reaction temperature (T_r) which can be obtained in lab protocols. Furthermore, the accuracy of the calculation depends on the precision of the input data, particularly the physicochemical properties (e.g., heat capacities) of the chemical substances being processed. It is recommended that the heat capacity be taken as an average value of the heat capacities at T_r and T_o if those values are accessible. When the solvent is a mixture, the specific heat capacity of this solvent blend can be estimated using the mole fraction average of its pure components, following the method outlined by Piccinno et al.[9] Under this framework, the heat capacity is simplified to that of the solvent and liquid reactants, disregarding the influence of solid reactants.

1.1 GENERIC MODEL

The heating/cooling system consists of a jacketed arrangement (either a double jacket for glass-lined reactor or a configuration with half-pipes for stainless steel reactor), through which heating and cooling fluids circulate.

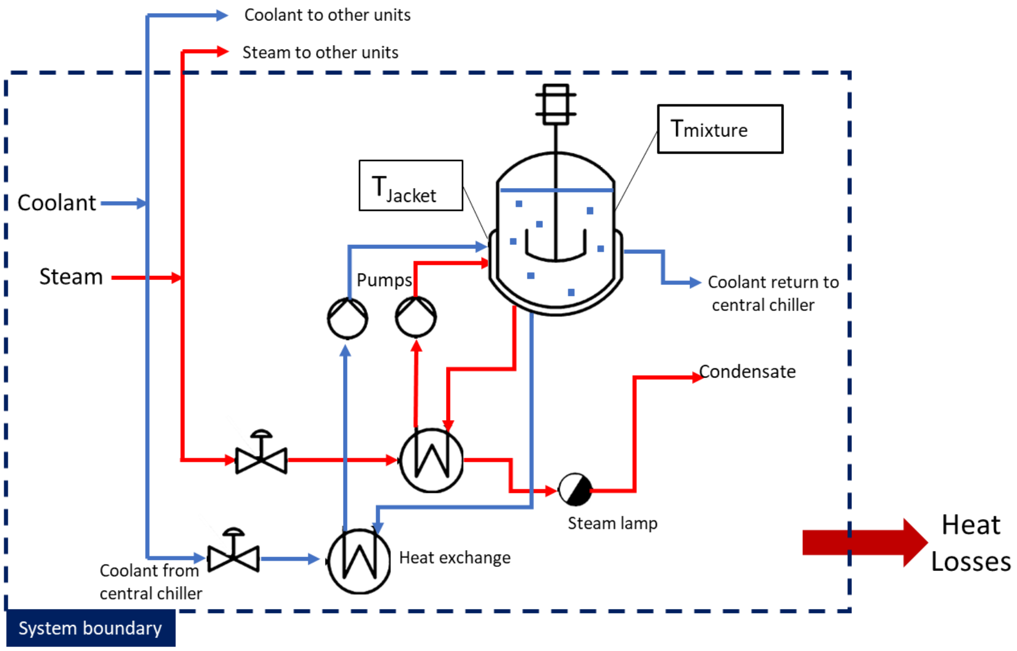

Figure S1. Generic batch reactor system for heat loss modelling.

<u>Existing bottom-up model equation considered in this study:</u>

$$Q_R = (m_{rx} \times C_{p,rx} + m_a \times c_{p,a} + m_{H/Csys} \times c_{p,H/Csys}) \times (T_r - T_0) + m_{evap,t} \times \Delta H_{vap} + r_t \times \Delta_r H \times t - Q_{Diss} + Q_{Loss}$$

(S2)

- Heat Loss Modelling: Initially considered all heat loss components and prioritized based on significance and accessibility of data to calculate.
- Removal of Less Significant Components:
    - The dissipated energy (Q_{Diss}) from the stirrer is almost negligible from the overall energy consumption point of view and represents only 1.5% of the overall energy usage in general.[8]

- Estimated evaporated solvent as a fugitive emission to the headspace and heat associated with evaporation $(m_{evap,t} \times \Delta H_{vap})$ for selected chemicals contributed negligible to the overall energy usage.
- Generally estimated reaction enthalpy $r_t \times \Delta_r H \times t$ for selected chemicals contributed negligible to the overall energy usage.

- Empirical Data Integration:
  - The average weight of the equipment (m_a) , specific heat capacity $(c_{p,a})$ and surface area of the vessel (A) for selected design material and scale were taken from industrial equipment manufacturers such as De Dietrict.[10] and Buchiglasuster.[11] that specifically supply the fine chemical and pharmaceutical industries, to ensure representativeness.
- Simplify application for LCA.
  - Temperature of the equipment (T_a) were assumed to be equal to the reaction mixture temperature which is accessible from lab data under pseudo-steady state.

Thermal loss modelling

Heat loss from the system (Q_{Loss}) must be compensated by additional energy to maintain a constant temperature throughout the reaction. This model accounts for losses due to temperature differences between process conditions and the ambient environment, incorporating an additional loss term to capture inefficiencies in energy transfer from the heating/cooling system, as represented by the Equation 3:

$$Q_{Loss} = a \cdot \left(Q_{Heat/Cool}\right)^b \pm k \cdot A \cdot (T_r - T_o) \cdot t_r \tag{3}$$

Here, a, b, and k are parameters of the thermal losses model assumed to be apparatus-dependent and constant over time for a given type of utility and specific equipment due to phenomenon such as radiation and free convection from the apparatus surface. These were developed through regression analysis for this study based on values calculated in the studies of Rérat et al.[12] and Szïjjarto et al.[13] The heat transfer area (A) in m^3 is obtained from equipment manufacturing catalogues rather than assumed or calculated. The reaction time (t) in sec is obtained from laboratory protocols. The heat loss terms can be replaced with the efficiency values given in the literature,

simplifying the calculation to Equation S5 (see Section 1.3). This allows efficiency terms specific to the apparatus to be applied when accessible or derived based on empirical data.

Apart from reaction-specific data obtained from laboratory protocols, reactor design parameters such as vessel weight and heat loss coefficients obtained from industrial equipment manufacturers such as De Dietrict.[10] and Buchiglasuster.[11] are provided in Table S5. These values serve as suitable substitutes for estimating energy usage when selecting the appropriate reactor type and scale, with stainless steel reactors serving as one example.

Table S5. Suggested scale-dependent data for the estimation of reactor's energy usage.

Stainless Steel				**Size (in litres)**				
**Physical entity**		**Symbol**	**Unit**	**500**	**1000**	**3000**	**5000**	**10000**
Reaction mixture volume		V_{mix}	m³	0.5	1.0	1.0	5.0	10.0
Surface area		A	m²	2.44	2.9	5.56	10.3	20.21
Reactor volume		$V_{(reac)}$	m³	0.7	1.3	3.6	6.2	11.0
Weight of Vessel		m_a	kg	930	1610	2590	4000	7720
Heat Capacity of Vessel		$C_{p,a}$	kJ/kg·K	0.50	0.50	0.50	0.50	0.50
Steam	Heat Loss Parameter 'a'	a	7.66	7.66	7.66	7.66	7.66	7.66
	Heat Loss Parameter 'b'	b	0.57	0.57	0.57	0.57	0.57	0.57
	Heat Transfer Coefficient	k	67.37	67.37	69.84	69.84	88.8	112.4
Brine	Heat Loss Parameter 'a'	a	7.39	7.39	7.39	7.39	7.39	7.39
	Heat Loss Parameter 'b'	b	1.49	1.49	1.49	1.49	1.49	1.49
	Heat Transfer Coefficient	k	59	59	59	59	59	59
**Glass-Lined Reactors**				**Size (in litres)**				
**Physical entity**		**Symbol**	**Unit**	**500**	**1000**	**2000**	**3000**	**5000**
Reaction mixture volume		$V_{mix}V_{mix}$	m³	0.5	1.0	2.0	3.0	5.0
Surface area		A	m²	2.64	4.5	7.23	9.33	13.74
Reactor volume		$V_{(reac)}$	m³	0.74	1.61	2.64	4.17	7.58
Weight of Vessel			kg	985	1700	2415	3470	5490
Heat Capacity of Vessel		$C_{p,a}$	kJ/kg·K	0.52	0.52	0.52	0.52	0.52
Steam	Heat Loss Parameter 'a'	a		6.85	6.85	6.85	6.85	6.85

	Heat Loss Parameter 'b'	*b*		0.74	0.74	0.74	0.74	0.74
	Heat Transfer Coefficient	*k*	W/m²·K	68	68	68	68	68
Brine	Heat Loss Parameter 'a'	*a*		10.85	10.85	10.85	10.85	10.85
	Heat Loss Parameter 'b'	*b*		0.64	0.64	0.64	0.64	0.64
	Heat Transfer Coefficient	*k*	W/m²·K	37	37	37	37	37

Simplified calculations

In scenarios where data such as mass ratios or specific heat capacities for complex mixtures are unavailable, a simplified approach can be used by applying Equation 1A (see Section 1.4). This method allows energy usage to be estimated under limited data availability by relying on standard reactor parameters and industrial-scale assumptions.

1.2 ADDITIONAL TERMS (S) FOR Q_R

The standard enthalpy of reaction is considered as an additional term only if it is explicitly mentioned in the data source and deemed necessary for the overall energy balance. If the relevant data is available, it can be calculated using Equation S3 by substituting values for the rate expression (r_t) in mol/sec, the standard molar enthalpy change of the reaction ($\Delta_r H$) in kJ/mol, and the reaction time (t) in seconds.

$$Q_{ent} = r_t \cdot \Delta_r H \cdot t \tag{S3}$$

Reactions involving gaseous reactants under elevated pressure such as hydrogenation, carbonylation, or ammonolysis are typically performed in liquid-phase systems, where the gas is introduced into the reactor under pressure and dissolves into the reaction mixture.

If gas-specific data is unavailable, a standard heat capacity ratio (γ), typically 1.4 for air or nitrogen, may be used as a practical default assumption for compression calculations. The energy demand for pressure changes is estimated as an additional term based on the electricity usage of the compressor, calculated using Equation S4 under the assumption of an isentropic (adiabatic and reversible) compression process for simplification:

$$E_{com} = \frac{P_r \cdot V_g}{\gamma - 1}\left[1 - \frac{P_o}{P_r}^{\frac{\gamma-1}{\gamma}}\right]/\eta$$

(S4)

This energy term is applied only when a gaseous reactant is compressed and introduced into a liquid-phase reaction system. Although the quantity of gas is typically small relative to the solvent or reactants, the compression energy can be non-negligible, particularly at elevated pressures or industrial scales.

Here, it is assumed that the compressor takes in gas at P_0=1atm (101,325N/m^2) and compresses it to the known reaction pressure (P_r) at an averaged efficiency η of 90%. The volume (V_g) of the gas phase in m^3 is calculated using the ideal gas equation with P_r values provided in lab protocols.

1.3 HEAT LOSS PARAMETERS AND EFFICIENCY

Heat loss parameters (a, b, and k) were derived from empirical data reported in literature and applied as scale factors for the heating and cooling medium (steam and brine) of batch reactors.[12,13] Regression models were fitted across reported values for different reactor sizes in order to extrapolate scaling relationships.

For heating in stainless steel reactors (steam), the regression for k (kW/m^{2}K) showed a strong linear fit (high R^2, see Figure S2) and was therefore retained as a scaling relationship (Eq. S5). For all other heat loss parameters (a, b) and for brine (cooling media), the regressions showed weak fits (low R^2). In these cases, we applied averaged values across reactor scales to improve robustness and avoid overfitting to poor correlations. This approach ensures consistency while still capturing realistic heat-loss performance.

The resulting parameters are valid only within the reactor size ranges reported in Table S5. Even within this domain, they should be interpreted as approximate scaling factors.

Equation retained for stainless steel reactors (steam heating):

$$k = 0.0651 + 4.74 \times 10^{-6} \times Scale \qquad (S5)$$

where Scale = reactor volume in litres

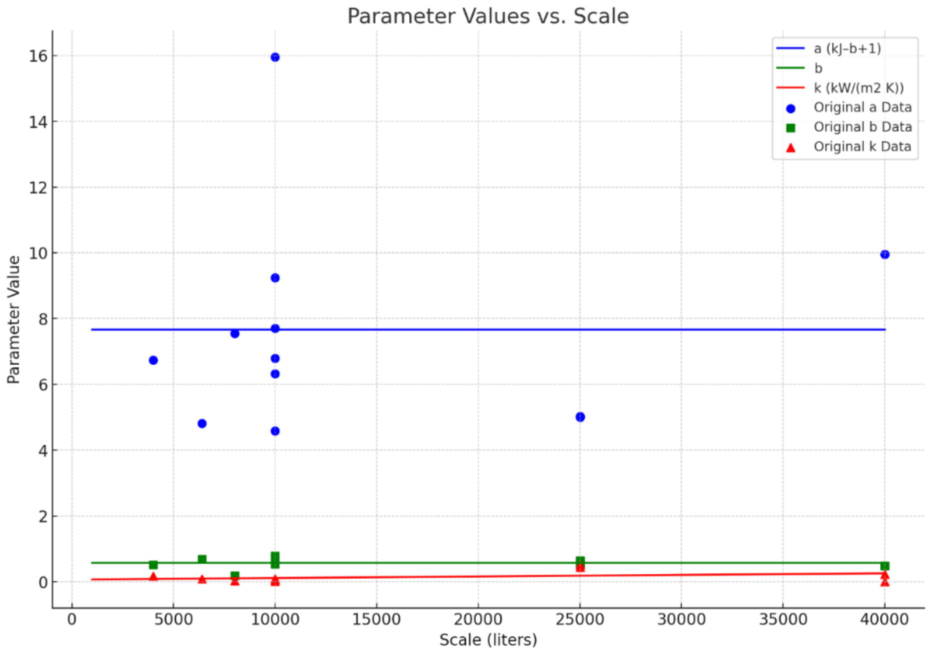

Figure S2. Regression analysis for parameters a, b, and k in heating (steam) system, illustrating the contrast between the strong fit obtained for k (steam, stainless steel) and the weaker fits obtained for other parameters, which justified the use of averaged values.

Simplified equation using efficiency

As a simplified alternative, heat duty or steam/coolant consumption of a batch reactor in kJ (Q_R) can also be estimated directly using an overall efficiency value η_e reported in the same source, without requiring parameters a, b, or k. This avoids the regression-based approach and provides a practical shortcut when only basic mass and heat capacity data are available.

$$Q_R = \frac{\left(m_{rm} \cdot C_{p,rm} + m_a \cdot C_{p,a}\right) \cdot (T_r - T_o)}{\eta_e}$$

Where η_e = 0.67 *(S6)*

1.4 SIMPLIFIED ESTIMATION: COMPLEX MIXTURES WITH LIMITED THERMAL AND MASS DATA

By applying this method, a conservative estimate of the reactor's energy requirements can be obtained based on reaction time and temperature. This ensures that the reactor's energy needs can be reasonably estimated, even without detailed data, maintaining the reliability and practicality of the energy estimation process.

$$Q_R = U \cdot A \cdot \left(T_{r,avg} - T_o\right) \cdot t_h + K \cdot A \cdot (T_r - T_o) \cdot t$$

(1A)

where U is overall heat transfer coefficient in kW/m²·K; t_h is the time (hours) taken to heat mixture from initial temperature to reaction temperature; K is loss coefficient in kW/m^2. Average temperature difference over the heating period is t_h. Since most of these parameters were unknown, assumptions were made, and values were derived based on appropriate industrial scale conditions. It is recommended to use the average U values from the given ranges in Table S6 (literature-based; values from Towler & Sinnott,[14] relevant to mixture characteristics and the heating medium, for equation 6. These ranges are standard for preliminary design and representative of industrial multipurpose batch reactors in fine chemical and pharmaceutical production.

Table S6: Generic Overall heat transfer coefficient for jacketed vessels.

Jacket medium	Mixture characteristics	U (W/m² K)
Steam	Dilute aqueous solutions	500-700
Steam	Light organics	250-500
Brine	Dilute aqueous solutions	200-500
Brine	Light organics	200-300

- The average temperature (T_{avg}) can be calculated as the difference over the heating period to provide a simplified approach, using Equation 2A. Assuming a linear temperature change from the initial to the final or reaction temperature offers a straightforward way to approximate the average conditions, especially when data is limited.
  In the study Bieler et al. (2004), batch reactors are modelled to be supplied with steam at 5 bars (151.8°C) & 15 bar (198.28℃) or heating purposes and cooling water of −1 °C, and brine composed of calcium chloride at a temperature around −28 °C to the cooling purposes based of the necessity.

$$T_{avg} = \frac{\left(T_j - T_o\right) + \left(T_j - T_r\right)}{2}$$

(2A)

- To calculate the heating time needed to raise the temperature from ambient (initial) to reaction (final) temperature, we use heating and cooling rates (H_r in K/min) specific to different reactor sizes. These rates are derived based on empirical data and are commonly cited in chemical engineering literature and textbooks. The heating time (t_h in hours) is calculated as:

$$t_h = \frac{(T_r - T_o)}{H_r \cdot 60}$$

(3A)

Based on values measured and reported in research studies and reactor equipment manufacturer data for the fine chemical and pharmaceutical industries,[15,16] heating and cooling rates have been observed in the following ranges: for small reactors (1,000 - 5,000 L), 1 K/min; for medium reactors (5,000 - 20,000 L), 0.8 K/min; and for large reactors (20,000 L and above), 0.5 K/min. When heating or cooling rates are not reported explicitly, they are calculated from published or vendor-reported energy performance data. These rates are suggested to ensure efficient thermal management during chemical processes and are considered representative of industrial practice for jacketed batch reactors.

2. CRYSTALLIZATION

Crystallization processes are modelled and upscaled by employing the same equipment of multipurpose reactors used for reaction steps. Multiple process design calculations are derived for different crystallization modes and are addressed through distinct process conditions outlined in laboratory protocols.

Cooling crystallization

This mode Involves cooling the solvent from a high to a low temperature at constant solvent composition. The energy required for cooling can often be neglected if the process occurs within the range of approximately 20-25°C (239 K-298 K). In this mode, solvents evaporation typically does not occur, and the cooling energy balance equation is similar to that used for steam consumption (Eq. 1), adjusted for cooling:

$$\boldsymbol{Q_{C,col}} = \frac{\left(m_{cx} \cdot C_{p,cx} + m_a \cdot C_{p,a}\right) \cdot (T_c - T_o) + m_c \cdot \Delta H_c}{\eta_e}$$

(4)

where m_c is the mass of crystallized material specified in the lab protocols, efficiency of the equipment (η_e), crystallization enthalpy (ΔH_c) in kJ/kg, if known. In general, the crystallization enthalpy is unknown due to the unique nature of the produced molecules, making it difficult to provide an averaged value.

Evaporative crystallization

In this process, supersaturation is generated as solvent mass is removed at a constant solubility rate. The heat of crystallization ($m_{cx} \cdot C_{p,cx}$) is often negligible compared to the heat transferred through vessel walls during batch crystallizations.[17] Equation 4 was altered by substituting the mass of the solvent evaporated (m_s) and enthalpy of vaporization (ΔH_{vap}) in kJ/kg.

$$\boldsymbol{Q_{C,evp}} = \frac{\left(m_{cx} \cdot C_{p,cx} + m_a \cdot C_{p,a}\right) \cdot (T_{boil} - T_o) + m_s \cdot \Delta H_{vap}}{\eta_e}$$

(5)

Antisolvent crystallization

Crystallization is induced by adding an antisolvent to a solvent to reduce solubility. Energy calculations for this process consider the thermal effect caused by the added antisolvent only if explicitly mentioned in the data source and deemed necessary for the overall energy balance.

Equation 4 is extended by including the product of the antisolvent mass (m_{as}) and its specific heat capacity (C_{as}) .

$$Q_C = \frac{\left(m_{cx} \cdot C_{p,cx} + m_a \cdot C_{p,a}\right) \cdot (T_c - T_o) + m_c \cdot \Delta H_c + m_{as} \cdot C_{as}}{\eta_e}$$

(4A)

If the required data are not available, providing universal values for the amount of antisolvent needed in crystallization processes is challenging due to significant variations based on the specific solute, solvent, process conditions, and other factors.

3. DISTILLATION

A simplified batch distillation column is considered basically similar to a multipurpose batch reactor with an addition of the distillation column on top (i.e., only the enriching part of a rectification column). It is equipped with a condenser unit possibility to split the evaporated and condensed solvent flow in a reflux and a distillate flow. Unlike a continuous distillation, the solvent to be distilled is filled into the reactor vessel as a reboiler at the beginning of the process, heated-up, and distilled until certain content either of the distillate or the bottom product is reached.

<u>*Heating energy*</u>

The heating energy for the batch distillation column derived as a modified version of batch distillation as shown in equation 6.

$$\boldsymbol{Q_{dist}} = \frac{Q_{Heat} + m_s \cdot \Delta H_{vap} \cdot (1.2 \cdot R_{min} + 1)}{\eta_{Loss}} \tag{6}$$

$$Q_{Heat} = \left(m_{dx} \cdot C_{p,dx} + m_a \cdot C_{p,a}\right) \cdot (T_{boil} - T_o) \tag{7}$$

In this context, a reflux ratio R_{min}, which represents the minimum ratio required for effective distillation and can be calculated using equation S7. m_s is mass of the liquid that needs to be vaporized, and ΔH_{vap} represents the distillate and enthalpy of vaporization. To simplify the scale-up framework, even multicomponent distillations are treated as sets of binary systems at ambient pressure, each requiring its own specific reflux ratio. Lower heat loss quantified for the batch distillation compared to reactors and Nutsche dryers mainly due to more direct heating of the vessel with steam condensing in the jacket (see Section 4.2).

Reflux ratio

$$R_{min} = \frac{X_{LF}}{\alpha - \theta} + \frac{(1 - X_{LF})}{1 - \theta} \tag{S7}$$

Where α is relative volatility of the solvents (>1) ; θ is a dimensionless parameter and correct must lie between the relative volatilities of the light key components and one, then: $1 < \theta < \alpha$. q represents the fraction of the feed that is liquid and ranges from 0 (saturated vapor) to 1 (saturated liquid); X_{LF}

is mole fraction of light key in feed. The parameter θ is determined by solving the nonlinear Underwood equation S8:

$$1 = \frac{X_{LF}}{\alpha - \theta} + \frac{(1 - X_{LF})}{1 - \theta}$$

(S8)

This equation can be solved numerically using Microsoft Excel's *what if analysis*; a step-by-step instruction for this calculation is provided in the Supporting Information 2. As relative volatility is not given in the lab protocols, equation S9 is typically used for preliminary estimates when detailed data are not available.[18]

$$log\alpha = \frac{T_2 - T_1}{T_{mix}}(3.99 + 0.001939T_{mix})$$

(S9)

where (T_2 – T_1) – boiling point difference of separating mixtures, °C; T_{mix} – boiling point of mixture; If the T_{mix} not available, it can be estimated using the mole fraction average of its pure components.

η_{Loss} calculations for simplified method

This approach was taken to provide a more accurate and specific basis for calculations compared to efficiency values based on expert estimates. The empirical heat loss values for the 9 batches (n=9) from Bieler et al.[8] can be considered under this generic model, assuming they are representative of typical batch reactors. As discussed in reaction, the distillations columns performance data in Bieler is representative of the equipment used in the fine chemical and pharmaceutical industries. From this dataset, an average η_{Loss} derived as a percentage of heat loss to total energy usages in distillation from this dataset.

$$\eta_{Loss} = \sum_{i=1}^{n} \frac{H_{Loss}}{Total\ energy - H_{Loss}} \cdot \frac{100}{n} = 0.78$$

(S10)

Cooling duty for condensation

When condensation requires cooling water, condensation energy can be calculated using below method. This value can be directly assigned as the cooling duty for the cooling water system. Assume that η_{cond} is equal to 0.8.

$$\boldsymbol{Q_{cond}} = \frac{m_s \cdot \Delta H_{cond} \cdot (1.2 \cdot R_{min} + 1)}{\eta_{cond}}$$

(8)

4. DRYING

Drying is a fundamental part of the isolation process, requiring the vaporization of the remaining wet fraction that was not removed in previous steps. In pharmaceutical processing, the drying operation is mostly preceded by filtration in the same equipment, as seen in Nutsche filter dryers specifically designed for the pharmaceutical sector, typically at limited scales. On the other hand, larger quantities of material can only be dried using rotary dryers that employ hot air for heating. Accordingly, the aforementioned dryer designs were modelled separately in this study, as energy usage is highly dependent on the type of dryer.

The PDCs for estimating energy usage were developed based on the simplified equation outlined by Bieler et. al.[8] As drying is an energy-intensive process where heat losses and heating of equipment are significant for the total production energy usage, these values were only roughly estimated in the other LCI estimation methods. Additionally, it is necessary to include the energy contribution from vacuum pumps such as Anti-Pollution Vacuum (APOVAC) for temperature-sensitive cases. To address this, the energy usage of the Nutsche dryer ($Q_{N.dry}$) equipped with vacuum pump can be calculated as follows:

$$\boldsymbol{Q_{Dry}} = Q_{Heat} + m_{liq} \cdot \Delta H_{vap} + Q_{loss} + \gamma \cdot P_N \cdot t_d$$

(9)

$$Q_{Heat} = \left(m_{liq} \cdot C_{p,liq} + m_a \cdot C_{p,a}\right) \cdot (T_{boil} - T_o)$$

(10)

$$Q_{loss} = K \cdot A \cdot \ (T_{boil} - T_o) \cdot t_d$$

(11)

where γ is the fraction of nominal power consumed by the equipment, P_N is the nominal power of the vacuum pump in kW, Q_{Loss} is the heat loss from the system in kJ. The mass of the liquid or solvent (m_{liq}) to be evaporated is obtained by comparing the weight before and after the drying step, as

stated in the lab protocol. Drying time (t_d in seconds) can be scaled from the laboratory drying time if given or estimated using conservative approaches in the absence of this data (see following section). Similar to reaction and distillation section, all other parameters must be adapted accordingly, whereas standardized values for three common scales were listed in Table S7 and are based on manufacturer catalogues industries such as Heinkel Process Technology.[19] and Separatech.[20]

Table S7. Suggested scale-dependent data for the estimation of energy usage of Nutsche filter dryers.

Parameter	Symbol	Unit	600 L	1000L	2000 L	4000L
Nominal Power of APOVAC Pump	P_N	kW	5.5	7.5	11	15
Fraction of Nominal Power Consumed	γ	-	0.8	0.8	0.8	0.8
Heat Transfer Coefficient	K	W/m²·K	47	47	47	47
Surface Area	A	m²	1.24	1.85	2.92	4.35
Equipment Weight	m_a	kg	2500	3500	6500	10000
Specific Heat Capacity of Air	$C_{p,\mathrm{a}}$	kJ/kg·K	1.01	1.01	1.01	1.01

Drying time (t_d)

a) This scaling assumes that the heat transfer rates in the larger-scale process remain constant, similar to those in the lab-scale setup. The relationship can be expressed as;

$$t_d = t_{lab} \cdot \frac{m_{liq}}{m_{lab}} \tag{12}$$

b) If the drying time is not reported in the lab protocol, we estimate it using evaporation rates (ER) derived from established chemical engineering references on industrial drying.[21,22] Direct empirical data for pharmaceutical Nutsche filter-dryers are scarce; therefore, we rely on values reported for vacuum-agitated drying systems, where drying proceeds predominantly under

constant-rate evaporation. Reported ranges of evaporation rate per unit area (kg/h·m²) from textbooks and literature are scaled to our equipment based on estimated heated surface areas of 1.24–4.35 m², representative of medium-scale pharmaceutical dryers operated under gentle agitation and moderate vacuum. The drying time is then calculated as:

$$t = \frac{m_{liq}}{ER} \tag{7A}$$

Evaporation Rate: 0.014–0.042 kg/sec

Considering that quantifying heat losses in a rotary dryer is challenging due to its low efficiency and the scarcity of data on the mean ratio of heat losses to heat input in the literature, an simplified estimation approach is discussed

Simplified method

We recommend using the energy efficiency specific for both types of dryers ($\eta_{\mathrm{N.dry}}$ and $\eta_{\mathrm{R.dry}}$) in the absence of quality data. This approach reflects the practical constraints of energy usage in terms of simplified Equation 6A, with efficiency values extracted from empirical measurements.

$$Q_{dry} = \frac{m_{liq} \cdot C_{p,liq} \cdot (T_{boil} - T_o) + m_{liq} \cdot \Delta H_{vap}}{\eta_{\mathrm{e}}} \tag{6A}$$

This equation was taken to provide a more accurate and specific basis for calculations in the absence of drying time compared to efficiency values based on expert estimates. The empirical heat loss values for the 6 batches (n=6) from Bieler et al.[8] can be considered under this generic model, assuming they are representative of typical wet cake drying. An average η_{Loss} derived as a percentage of heat loss to all other energy usages from this dataset.

$$\eta_{Loss} = \sum_{i=1}^{n} \frac{H_{Loss}}{Total\ energy - H_{Loss}} \cdot \frac{100}{n} = \mathbf{0.67} \tag{S10}$$

5. STIRRING ENERGY

The consumption of stirring energy (E_{stir}) during chemical reactions can be calculated using Equation 13, as proposed by Piccinno et al.[9], which includes the type of impeller (characterized by the

power number N_p), impeller diameter (D) in m, rotational speed (N), density of the reaction mixture (ρ_{mix}), and reaction time (t). This relationship is mathematically expressed as:

$$E_{stir} = \frac{N_p \cdot \rho_{mix} \cdot N^3 \cdot D^5 \cdot t}{\eta_{\text{stir}}} \quad (13)$$

where η_{stir} represents the stirring efficiency, accounting for dissipated energy due to friction and other inefficiencies. In contrast to aforementioned approach, the power number (N_p) is altered and not held constant but varies based on different impeller types and reactor scales, providing a more accurate representation of mixing intensity under varying operational conditions.

Table S8. Suggested scale-dependent data for the estimation of energy usage of stirring energy.[9]

Physical entity	Symbol	Unit	100 l	500 l	1'000 l	5'000 l	10'000 l
Impeller diameter	*d*	m	0.173	0.296	0.373	0.638	0.803
Power number of impeller	*Np*	–	0.79 Axial flow	0.79 Axial flow	0.79 Axial flow	0.79 Axial flow	0.79 Axial flow
	Np	–	3.44 Radial flow	3.44 Radial flow	3.44 Radial flow	3.44 Radial flow	3.44 Radial flow
Rotational speed of agitator	*N*	1/s	3.052	1.785	1.417	0.828	0.658
Efficiency of agitator	*ηstir*	%	90	90	90	90	90

In situation where detailed input data is limited due to lot of combinations in stirring setups, a simplified method involves calculating energy values using nominal power of agitator (P_N) in kW sourced from Towler & Sinnott.[14] for different applications (Table S8). These motor power values are then multiplied by the operation time to estimate energy usage, reflecting a worst-case scenario assumption to ensure practical feasibility.

$$E_{stir} = P_N \cdot t \quad (8A)$$

Table S9. Generic nominal power of agitator for jacketed vessel.

Agitation	Application	Power, kW/m³
Mild	Blending, mixing	0.04 – 0.10

Medium	Homogeneous reactions	0.01 – 0.03
	Heat transfer	0.03 – 1.0
	Liquid-liquid mixing	1.0 – 1.5

6. OTHER API SYNTHESIS OPERATIONS

Homogenizer

Various types of homogenizers can be distinguished, including high-pressure, ultrasonic, and rotor-stator designs. Within this framework, only the rotor-stator type is considered. This device operates similarly to stirring but at much higher shear rates. Consequently, the energy requirement is calculated using the same equation as for stirring, with adapted parameters and standardized values from Table S10. The power number is comparable to that of standard agitators, and an average value was determined based on data from Piccinno et al.[9] However, homogenizers generally feature smaller impeller diameters than conventional agitators. To address this, manufacturer data were consulted to establish typical diameters and shear rates. Where impeller details were unavailable, a rotor-stator ratio of 0.9 reflecting the maximum observed value was assumed, along with maximum shear conditions. Due to the elevated shear rates, homogenization consumes significantly more energy than stirring. As a result, these steps can substantially affect the overall LCA outcome. Furthermore, because the energy calculation depends on the impeller diameter raised to the fifth power, small variations can greatly influence the results. A sensitivity analysis is therefore recommended, supported by manufacturer data where possible.

Table S10. Suggested scale-dependent data for the calculation of the rotor-stator homogenizer energy draw. [9]

**Physical entity**	**Symbol**	**Unit**	**100 l**	**500 l**	**1'000 l**	**5'000 l**	**10'000 l**
Impeller diameter	d	m	0.072	0.111	0.139	0.260	0.288
Power number of rotor	N_p	–	2.39	2.39	2.39	2.39	2.39
Rotational speed of rotor	N	1/s	48.333	48.333	48.333	20	20
Efficiency of agitator	η_{stir}	%	90	90	90	90	90

SECTION 5: PRODUCT FORMULATION

7. WET GRANULATION

The high shear granulator, a common type of wet granulation in pharmaceutical manufacturing and it was selected for agglomeration of smaller particles into larger granules to improve the flowability and compressibility for further processing. It uses an impeller to mix the solid powder blend and the liquid together.[23,24] The torque profile applied by the impeller motor was used to calculate energy usage.[25]

$$E_{wg} = \int_{0}^{T_{end}} P_{wg} . dt_{wg}$$

$$E_{wg} = P_{wg} \times \left(\frac{\omega_{operating}}{\omega_{max}}\right) \times t_{wg}$$

(S11)

- Simplify application for LCA:
  - Energy consumes (E_{wg}) various with the mixing time (t_{wg}) as power consumes (P_{wg}) changes with the quantity of binder added along the time. As a result of this, the operating impeller speed changes with the quantity of binder added. Consequently, the operating impeller speed ($\omega_{operating}$) adjusts over a wide range. For modelling purposes, $\omega_{operating}$ is assumed to be half the value of the maximum impeller speed (ω_{max}), as a rough estimate, based on previous observations of impeller speed variation.[24]
  - The only available data from accessible manufacturing sources is granulation time which may increase with scale, while the binding addition flow rate remains constant. However, for the context of LCA, t in lab protocol is assumed to remain constant after scaling up.
- Empirical Data Integration:
  - The average motor rating (P_{wg} $in\ kW$) and maximum impeller speed (ω_{max}) were taken from manufacturer catalogues (Table S11). For medium- to large-scale high-shear wet granulators, such as the GEA PMA series (e.g., PMA-1800 with a 75 kW

impeller drive operating up to 120 rpm), these specifications are representative of equipment commonly employed in industrial pharmaceutical production systems such as GEA pharma systems.[26]

If data is available, recommended to use equation S11. For the context of LCA, the equation presented to energy usage (E_{wg}) in the same study has been refined into a simplified version adjusted for the accessible lab protocol data as follows.

$$E_{wg} = P_{wg} \cdot 0.5\, t_{wg} \quad (15)$$

where mixing time (t_{wg} in hours) is taken from lab protocols as a rough estimate and average motor or power rating (P_{wg}) in kW for the selected design and scale were obtained from literature.

Table S11. Suggested scale-dependent data for the estimation of fluidized bed drying energy usage and comparison with empirical data.

Unit Operation	Parameter	Typical Value	Units	Remarks
Wet Granulation	P_{wg}	70-85	kW	Motor power of chopper also considered
	ω_{max}	120–150	rpm	
Fluidized Bed Drying	α	14,400	m³/h	
	P_{comp}	20 - 100	kW	Pumps normally consume 11 kW for 3200 m³/h air
	$C_{p,air}$	1.005	kJ/(kg·K)	
	T_o	298.15	K	
	P_{air}	1.2	kg/m³	
	Q_{fb}	10-50	MJ/kg	Thermal energy of 10–15 MJ/kg water evaporated typical (possible range up to 50 MJ/kg with losses)
	K_{wa}	50-100	W/m² K	This range is acceptable for modeling industrial-scale fluidized bed dryers
	A_L	10-20	m²	

Milling	P_{mil}	7.5-15	kW	Manufacturer data of conical mill for large scale for 800kg/h – 7000kg/h
Blending	P_B	5.5-75	kW	Main motor 11kW for 500000 tablets/h (assuming 200-500mg tablet)

8. DRY GRANULATION

Dry granulation is another type of granulation process which is less common compared to wet granulation. In this process, the active drug is mixed with ingredients that are inherently granular and cohesive properties to form granules without the use of liquids. Roller compaction is considered in this study as a commonly employed and energy-efficient process for this unit operation.[27,28] Before roller compaction process, blending and mixing are essential steps to ensure the uniform distribution of the active pharmaceutical ingredient (API) and excipients for achieving a homogeneous mixture.

As laboratory protocols rarely provide process conditions for dry granulation, we derived a simplified energy expression based on the mass of granulated mixture processed. For this purpose, manufacturer specifications for commercial roller compactors were used, which typically report motor ratings of 4.8–24 kW and throughputs of 20–300 kg/h.[29-32] These values correspond to specific energy consumptions of approximately 0.05–0.3 kWh/kg, from which an average representative coefficient of 0.131 kWh/kg was adopted to derive Equation.

$$\boldsymbol{E_{dg}} = 0.131\frac{kWh}{kg}\cdot m_g \tag{16}$$

where E_{dg} is the energy usage in kWh and m_g is the mass of the ingredient mixture subjected to dry granulation (kg).

9. FLUIDIZED BED DRYING

In the pharmaceutical industry, fluidized bed drying is a vital process and widely used drying technique to remove moisture, ensuring stability of products.[33] The fluidized bed dryer operates by passing hot air through a bed of particulate material, causing the particles to become suspended

and facilitating efficient heat and mass transfer. The energy usage for drying (Q_{dryer}) can be determined using the following equation:

$$\boldsymbol{Q_{fb}} = C_{p,air} \cdot (T_{dry} - T_o) \cdot \alpha \cdot \rho_{air} \cdot t_{fb} + 3600\, P_{comp} \cdot t_{fb} + Q_{loss} \quad (17)$$

Where α represents the inlet air flux in m^3/h, P_{comp} is the motor rating of the compressor in kW, $C_{p,air}$ is the specific heat capacity of air kJ/kg, T_{dry} and T_o are the drying (or inlet) and ambient temperatures respectively, ρ_{air} is the density of air in kg/m^3 and drying time (t in hours). The inlet air flow value of 14 400 m^3/h used for substitution in Equation 17 was calculated from the air velocity data reported by Sampat et al.[25] This magnitude is consistent with catalogue specifications for medium- to large-scale pharmaceutical fluidized bed dryers, supporting its representativeness.

The calculation of the Q_{loss} term, which accounts for heat losses, is stated Equation 18. In scenarios where the drying time (t) is not specified, but the initial and final moisture contents (C_i and C_f) are provided, the energy required for drying can be estimated using the enthalpy change Equation 9A.

Use average power values within the model-specific ranges reported in Table S11, sourced from manufacturer catalogues (e.g Diosna,[33] SanityCo.[34] and NU Pharm.[35]) and compared with values reported in industrial case studies.[36,37] For Table S11, values from both sources were used to define representative ranges for medium- to large-scale pharmaceutical equipment.

Heat loss from the fluidized bed dryer ca

$$Q_{loss} = K_{wa} \cdot A_L \cdot (T_{dry} - T_a) \quad (18)$$

where K_{wa} is the overall heat transfer coefficient ($W/m^2 \cdot K$) and A_L is the heat transfer area of the equipment (m^2). The equipment specification was obtained from peer review articles and manufacturing catalogues.[38-41] K_{wa} ranges from 50 to 100 $W/m^2 \cdot K$ and A_L ranges from 10 to 20 m^2. It is recommended to use averaged values for the calculation.

In scenarios where the drying time (t) is not specified, but the initial and final moisture contents (C_i and C_f) are provided, the energy required for drying can be estimated using the enthalpy change equation.

$$Q_{fb} = \Delta H \times (C_i - C_f) \quad (9A)$$

This method takes into account the heat of vaporization (ΔH) of water necessary to remove the specified amount of moisture from the material. For simplicity and practical purposes, it is common to approximate the heat of vaporization as the heat of vaporization for water, which is approximately 2260 kJ/kg.

10. MILLING

Milling is utilized as a post-granulation step to de-lump cohesive materials, ensuring that oversized granules are broken down into smaller granules that conform to product size specifications.[42] Energy usage for milling ($\boldsymbol{E_{mil}}$) is estimated based on the motor power (P_{mil}) of the conical mill, using the following empirical relationship. [25]

$$\boldsymbol{E_{mil}} = P_{mil} \cdot 0.75 \cdot Load\ fraction \cdot t_{mil} \tag{19}$$

This equation assumes a motor efficiency of 75% and a typical operating load fraction of 75% as a conservative estimate. The appropriate motor power values for commercial-scale milling obtained from industrial manufacturers such as Hanningfield.[43] are provided in Table S11.

$$\boldsymbol{E_{mil}} = P_{mil} \cdot 0.5\, t_{mil} \tag{20}$$

11. MIXING AND BLENDING

As this process is utilized in multiple steps of product formulation, it is important to consider mixing and blending. However, if the duration of the operation is known from lab protocols, it is still possible to estimate the energy usage roughly by multiplying the accessible power values by the operation time. For this estimation, it is assumed that both mixing and blending are performed using a V-blender, which is one of the most commonly used equipment in pharmaceutical manufacturing. Power values (P_B) normally ranges between 5-50kW and it is recommended to use the average value for rough estimation.[44]

$$E_{mixing} = P_B \cdot t_{mixing} \tag{21}$$

12. TABLET COMPRESSION (OR TABLETING)

Tablet compression is a critical step in pharmaceutical manufacturing where the blended powder mixture is compressed into tablets by ensures that the tablets have the required mechanical strength and uniformity. Rotary tablet presses are considered in this study due to their widespread use and efficiency in large-scale production.

As laboratory protocols rarely provide process conditions for tablet compression, a simplified energy expression was derived based on the mass of granulated mixture processed. Empirical energy consumption data reported by Sharma et al.[37] for paracetamol tablet manufacturing, where equipment-specific energy use was normalized by the processed batch mass, served as the basis. These values are consistent with real manufacturing data and commercial equipment specifications; for example, the KORSCH XL 800 rotary tablet press can produce over 1 million tablets per hour, confirming that this value lies within the representative industrial range.

$$\boldsymbol{E_{tc}} = 0.045 \frac{kWh}{kg} \cdot m_f \tag{22}$$

where E_{tc} is the energy usage in kWh for the final ingredient mixture (m_f) processed during tablet compression in kg:

SECTION 6: AUXILIARY OPERATIONS

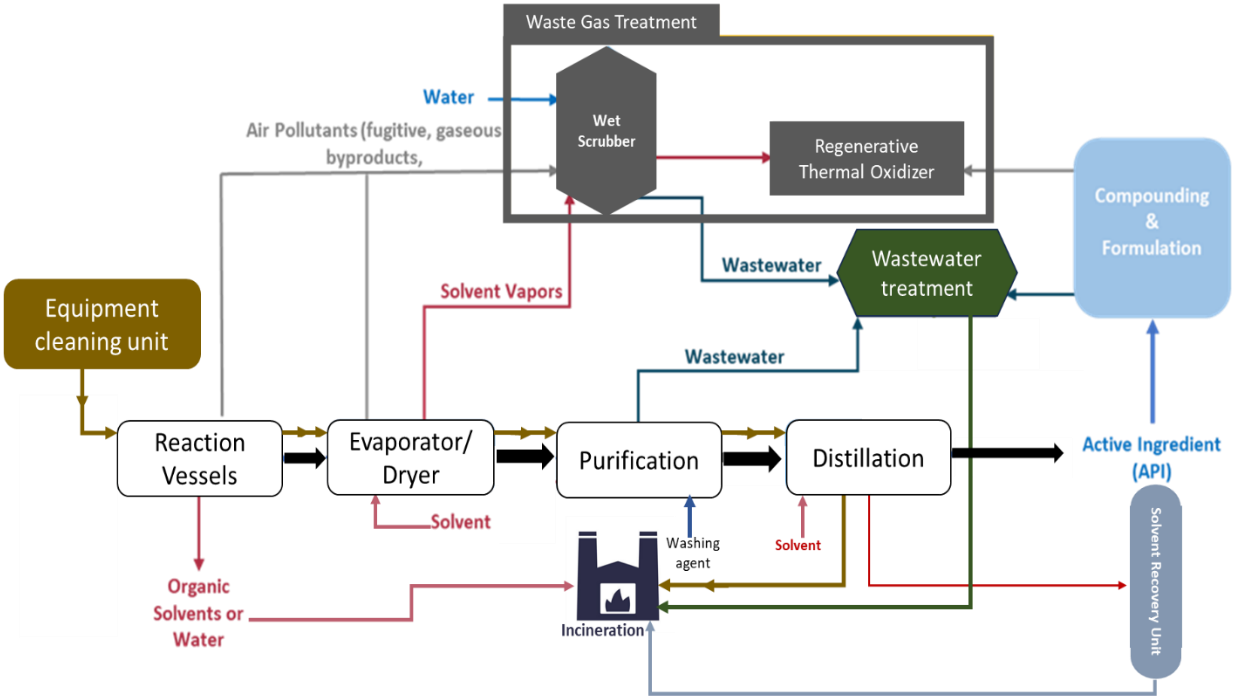

Figure S3. Process Flow Diagram Illustrating the Main Waste Streams in a Generalized Pharmaceutical Production.

13. SOLVENT RECOVERY

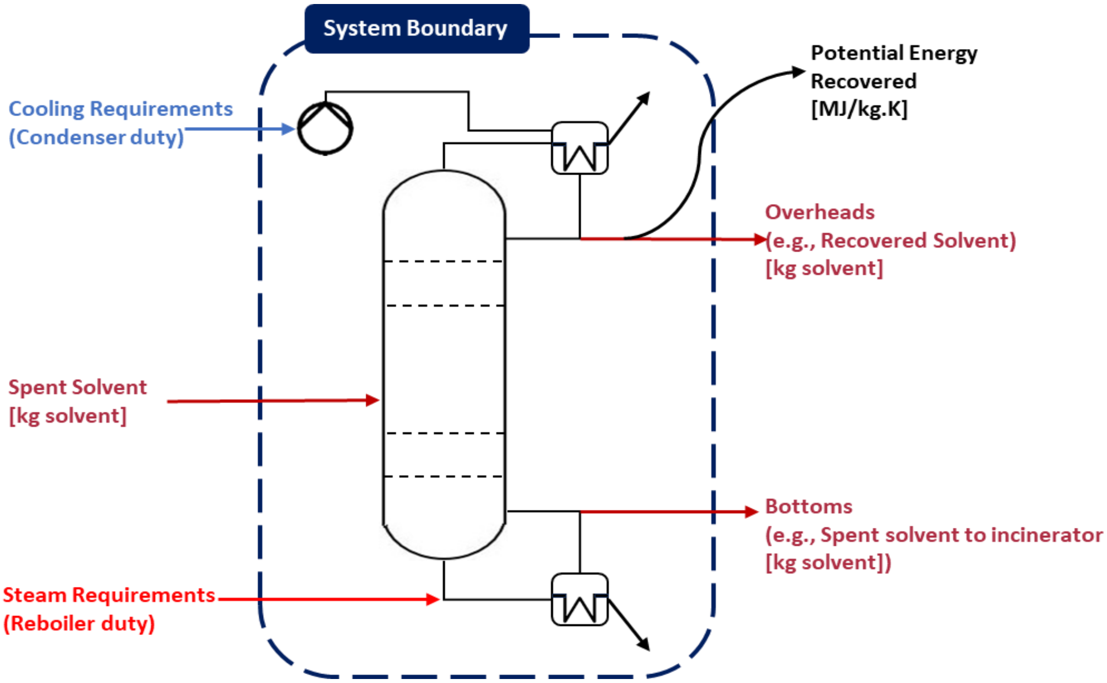

Figure S4. Schematic of the generic solvent recovery model.

Typical waste streams from pharmaceutical manufacture contain 75–80% solvent.[45] Therefore, large quantities of solvent waste are generated which must be disposed of or recycled. Often this waste is disposed of through incineration either on-site or externally.[46,47] As distillation was found to be an environmentally feasible method, [48-50] many chemical industries especially pharmaceutical industry, have implemented it as a sustainability production measure.

Distillation is commonly used for 95% of all chemical industrial solvent separation processes because a high recovery and purity can typically be achieved.[51,52] In the pharmaceutical industry, it is common for a single synthesis process to generate multiple solvent streams. These mixed solvent wastes are often collected in a single tank and subsequently undergo distillation, a widely used and efficient method for solvent recovery.[53] This practice allows for the separation and purification of valuable solvents, significantly reducing environmental waste. In general, solvent recovery rates in the pharmaceutical sector are reported to vary, but for this framework a recovery rate of 75% is assumed.

Some facilities employ continuous separation and purification systems as part of an integrated process, while others opt for batch processing of waste streams periodically. The specific approach can vary depending on the nature of the solvents and the production processes involved. However,

despite the efficiency of solvent recovery, the pharmaceutical industry often does not reuse the recovered solvents for their own synthesis processes due to the stringent purity requirements. Instead, these recovered solvents are typically sold to other industries where such high purity levels are not as critical. This practice not only helps in managing waste but also provides an additional revenue stream.[54,55]

To estimate the energy usage, the calculations method described in the distillation section 3.1 can be used as the starting point with adaptations. For modelling the solvent recovery module, a generalized model of a continuous distillation column was used as shown in Figure S4, as this has become established in the chemical industry with the increasing implementation of energy-saving actions. However, in continuous distillation processes, the dynamics differ significantly, requiring the adaptation of the batch distillation energy equations by considering the variation in empirical or process-simulated data. This is expressed in the following equation:

$$Q_{Codi} = 0.78\ Q_{dist} \tag{23}$$

Here, 0.78 is an empirically derived factor that accounts for the differences between batch and continuous distillation. This factor is calculated based on empirical values from literature for setups where the same solvent mixture is distilled under both batch and continuous conditions.[56]

Azeotropic Mixtures and Efficiency Adjustments

In the pharmaceutical industry, it is common to encounter waste streams comprised of azeotropic mixtures, which complicates the distillation process. It is recommended to consider the mass fractions of waste streams in the production process, especially when multiple solvents are involved, and to check for the possibility of azeotropic mixtures, as illustrated in Table S12.

Table S12: Common azeotropic systems in the pharmaceutical and chemical industry.

Azeotrope System	Azeotropic Composition (wt%)	Azeotrope bp (°C)
Acetonitrile–Water	83.7% ACN / 16.3% H_2O	76.5
Ethyl acetate–Ethanol	69.2% EA / 30.8% EtOH	71.8

Methanol–Methyl methacrylate	84.5% MeOH / 15.5% MMA	64.2
Diethoxymethane–Toluene–Ethanol	Varies	74–76
Ethanol–Water	95.5% EtOH / 4.5% H_2O	78.1
Isopropyl alcohol–Water	87.7% IPA / 12.3% H_2O	80.4
Formic acid–Water	77.5% FA / 22.5% H_2O	107.3

Conventional continuous setups for azeotropic mixtures are highly energy-intensive due to the formation of non-ideal mixtures. To address this, Equation 23 was modified to account for azeotropic behaviour and process efficiency. However, novel distillation technologies, such as heat pump-assisted extractive distillation and pervaporation-distillation hybrid processes, have been introduced as energy-saving alternatives. These technologies offer improved energy efficiencies and can be integrated into the above equation to provide more accurate energy estimates as a conservative approach:

$$Q_{aze} = Q_{Codi} \times \frac{f_{ac}}{\eta_{ac}} \tag{24}$$

The correction factor $\boldsymbol{f_{ac}}$ is specific to azeotropic mixtures and reflects the energy demand variation. This factor is derived from empirical data where azeotropic mixtures have been distilled in both convectional continuous and novel distillation setups. Here, η_{ac} represents the energy efficiency factor specific to the distillation technology employed, such as heat pump-assisted or pervaporation-distillation hybrid processes.

Values for f_{ac} and η_{ac} were derived from peer-reviewed simulation studies of extractive distillation, pressure-swing distillation, and hybrid systems such as pervaporation-assisted distillation. Reported energy savings from heat pump integration and hybrid configurations ranged from 20% to over 50%, with thermodynamic efficiencies improving by up to 85% in some cases. These suggested range of values used in this study are provided in Table S9. While these factors are system-specific and based

primarily on modeled data, they offer a literature-grounded means of estimating energy use in the absence of detailed simulation.

Table S9. Literature-derived correction and efficiency factors for energy estimation in azeotropic distillation.

Distillation Technology	Azeotropic Correction Factor f_{ac}	Efficiency Factor η_{ac}	Energy saving remarks	Source
Pressure-swing distillation (PSD)	1.2 – 1.8	1.0 – 1.4	Can reduce entrainer use; often energy-intensive unless integrated	65
PSD with full heat integration (e.g., EFHI-PSD)	1.2 – 1.8	1.7 – 2.0	Up to 53.4% reduction in energy consumption reported	65
Extractive distillation with heat pump (VRHP-ED)	1.4 – 2.0	1.5 – 2.5	26.18-49.18% reduction in energy consumption reported	66,67
Extractive distillation with pervaporation hybrid (EDPV)	1.3 – 2.2	2.0 – 4.0	>40% energy reduction vs. extractive alone; strongly composition dependent	68,69
Membrane-assisted (e.g., pervaporation-only)	1.5 – 3.0 (if used alone post azeotrope)	3.0 – 6.0	Pervaporation after distillation to azeotrope can reduce energy by 50–85%	69
Heat pump-assisted basic distillation (no azeotrope)	— (no azeotrope correction)	1.3 – 1.8	Applies to close-boiling mixtures or binary separations	66
Dividing-wall column (DWC) for azeotropes	1.2 – 2.0	1.2 – 1.6	Limited use for azeotropes; better for multi-component splits	67

Simplified approach

An empirical study of waste solvent distillations in Switzerland reported that continuous distillation requires on average 1.21 kg of steam per kg of waste solvent.[56] Reported values range from 0.42 to 2.57 kg of steam, highlighting the case-specific nature of the consumption. When heating energy cannot be derived from the presented equations due to missing information, the empirical value of 1.21 kg steam/kg waste solvent can be applied for scale-up. For consistency of this framework and to obtain case-specific estimates, distillation energy should be calculated with the equations whenever possible, with comparison to empirical data supporting scenario analyses. To account for

condensation, 0.027 kg of cooling water per kg of waste solvent can be included in the LCA as an empirically derived input.[56]

14. HEAT RECOVERY

To reflect practical energy-saving strategies in industrial pharmaceutical settings, a moderate level of heat integration was assumed. Although literature on batch operations reports theoretical waste-heat recovery potentials of up to 85% under ideal conditions, full-scale implementations capture only a fraction of this potential.[70] Even in Stamp and Majozi.[63] optimized case study with an ideal heat-storage design, the external hot-utility demand was reduced by about 33%, far below the theoretical maximum. Empirical studies and case examples further show that industrial batch plants, such as those producing resins or fine chemicals, achieve only about 8–20% reductions in heating energy when heat-integration measures are applied.[71] Importantly, one pharmaceutical facility that implemented pinch analysis combined with flue-gas recovery and warm-water storage achieved approximately 20% reduction in overall thermal energy use after upgrades.[72] On this basis, this study applies a conservative and representative heat-recovery factor of 20% for API synthesis operations, consistent with reported industrial experience. No heat recovery is assumed for auxiliary operations such as cleaning, HVAC, and formulation, where integration is not commonly practiced or technically feasible.

A sensitivity analysis could be performed by varying the recovery factor across a range of values to represent minimal and advanced integration scenarios. This would allow assessment of the influence of this assumption on total energy demand and LCA outcomes.

15. AIR HANDLING UNIT (AHU): HVAC

Air Handling Unit (AHU) of HVAC systems are crucial in the pharmaceutical industry to maintain strict environmental control, ensure product quality, comply with regulatory standards, and provide indoor thermal comfort for workers. Many studies have highlighted the AHU contribute 20–40% of the final energy usage in pharmaceutical production facilities and present opportunities to reduce energy usage.[72-74] However, despite the higher contribution of this unit system for impacts such as global warming, previous pharmaceutical industrial LCAs and LCI estimation frameworks often excluded HVAC system components due to allocation complications and lack of site-specific data.[75]

Given the importance of HVAC systems for sustainability, the energy usage of these systems (Q_{AHU} in kW) can be calculated as a function of the production duration of a batch (t_p in hours). By incorporating production hours into the calculation, we can allocate the AHU energy usage to the specific production process and capture the variation among different productions.

To improve representativeness and transparency, we distinguish between two approaches:

1. Recommended approach (applied in the case study)
2. Simplified approach (illustrative, when production hours are not available)

1. Recommended approach

The recommended approach uses data from the Industrial Assessment Center (IAC) database of 68 pharmaceutical production facilities.[44] This dataset reports total annual energy consumption (Q) in terms of natural gas and electricity, floor area (A), and annual production hours (H). To ensure robustness, the IAC dataset was filtered to include only generic drug and tablet formulation facilities (excluding biopharmaceuticals).[76]

To ensure uniformity in the energy values across the diverse dataset, AHU energy usage per production hour (E) is calculated using the following equation:

$$E = \frac{Q}{A \cdot H} \times AHU_{PC} \qquad (S12)$$

Here, AHU_{PC} refers to the contribution of the AHU system to the total energy consumption of the pharmaceutical industries per batch production. This value is not directly available in the literature.[72-74] The most commonly cited value in the literature is approximately 21% for a production plant, regardless of factors such as industrial scale, number of production lines, and weather conditions.

In our baseline calculation, we assumed two production lines per facility, corresponding to a 10.5% contribution per line or per product. Sensitivity analyses were also conducted with 2, 5, and 10 production line scenarios, corresponding to allocations of 15%, 10.5%, 4.2%, and 2.1% of total energy to each line/product, respectively.

For each of the 68 plants in the IAC dataset, the AHU energy usage per production hour (Q_{AHU}) was first calculated individually by combining the estimated AHU energy use from natural gas and electricity with production hours and the assumed HVAC contribution. Instead of taking a simple arithmetic mean, the distribution of Q_{AHU} values was analysed. A lognormal distribution was fitted to

the 68 plant-level values. The median of this fitted distribution was used as the baseline reference value to derive Equation 25, while the 10th and 90th percentiles served as lower and upper bounds in the sensitivity analyses.

$$\boldsymbol{Q_{AHU}}\ per\ plant = E_{natural\ gas} + E_{electricity}$$

The following equation resulted for total energy consumed by AHU in kWh for a hour of production for a pharmaceutical plant,

$$Q_{AHU} = 602.6\ kW \cdot t_p \tag{25}$$

2. Simplified approach

In cases where production duration is not available or cannot be estimated, a simplified method can be applied using median total energy consumption per kilogram of product used for the industrial reference value (see section 'Reference industrial values') . Importantly, the 21% HVAC contribution was applied to this median total energy consumption. The resulting empirical value is 44.1 kWh per kilogram of pharmaceutical product.

16. EQUIPMENT OR FACILITY CLEANING

Cleaning of pharmaceutical processing equipment is a critical operation when switching products in multipurpose facilities to prevent cross-contamination of drug substances and drug products with undesirable residues from previous batches. Therefore, for batch reactors and other vessel equipment, both in-between batch cleaning and cleaning before product switches are performed until recommended cleaning evaluation limits are met.[77,78]

Most pharmaceutical industries use a solvent-based cleaning procedure that comprises four stages: pre-rinsing, main solvent cleaning, post-rinsing, and air drying, typically requiring extensive cleaning with organic solvents and water.[80] However, the challenge lies in limiting the use of solvents while ensuring adequate cleaning as a sustainability measure.[81] It is assumed that the same cleaning procedure is followed for various vessel equipment such as batch reactors, dryers, crystallizers, and others with known volumes with head space.

For each cleaning stage, PDCs for energy usage were derived from a base equation indicated by Palabiyik et al.[81] as shown below and simplified equation based on the specifications of the equipment.

$$E_{cleaning} = V \cdot C_p \cdot \left(T_{liq} - T_o\right) + \frac{V \cdot \rho \cdot h \cdot g}{\eta_{pump}} + E_{dry}$$

(S13)

- Removal of Less Significant Components: Energy usage for the air drying (E_{dry}) is assumed to be negligible compared to total cleaning energy.
- Empirical Data Integration:
  - The temperature of the cleaning solvent is considered to be at its boiling point for effective cleaning (T_{liq}).
  - Due to lack of data to calculate the pump energy from $V \cdot \rho \cdot h \cdot g/\eta_{pump}$, a simplified method estimated using the nominal power of the pump (P_{pump}).
- Simplify application for LCA.
  - The volume of the water or solvent required (V) for sufficient cleaning of the vessel is assumed as 20% of the volume of the vessel equipment.
  - The initial temperature of the water and solvent before heating for cooling is assumed to be the ambient temperature.

Accordingly, energy usage for all stages (except air drying) for each equipment can be calculated by adding the energy required to drive the pump and the thermal energy needed to heat the cleaning water or solvent:

$$\boldsymbol{E_{clean}} = [0.2V \cdot \rho_s \cdot C_{p,s} \cdot \left(T_{boil,s} - T_o\right) + P_{pump} \cdot 2t_s] + [0.4V \cdot \rho_w \cdot C_{p,w} \cdot (T_w - T_o) + P_{pump} \cdot 4t_w]$$

(26)

Where $E_{cleaning}$ was energy consumed in megajoule (kJ), $C_{p,s}$ was heating capacity of water or methanol used. V (m^3) was volume of the equipment (refer Tables S5, S7 & S10), T represents the operational temperature of water (w), boiling temperature of solvent (s), the ambient temperature (o), in K respectively, nominal power of the pump (P_{pump} in kW) and the duration of the cleaning (t).

The recommended procedure and operational temperatures for solvent-based cleaning were taken from studies conducted by Aramouni et al.[82] & Piepiórka-Stepuk.[83] Time for a cleaning stage (t_s or t_w) derived as

$$t_s \; or \; t_w = \; 0.2\frac{V}{F.A}$$

(27)

The typical flow rates (F) of 0.03 – 0.06 L/m²·s, considered for solvent-based cleaning in the pharmaceutical industry, are based on industry best practices and guidelines provided by equipment manufacturers and literature. Therefore, different vessels have different cleaning times according to the derived equation.

17. AIR EMISSIONS CONTROL

Pharmaceutical and chemical industries emit various pollutants [84], including volatile organic compounds (VOCs), particulate matter, nitrogen oxides, and greenhouse gases. These pollutants can significantly impact the environment if not properly treated. The majority of emissions from batch chemical manufacturing operations come from volatile solvents that evaporate during manufacturing steps (EIIP, 2007). To identify possible emission sources, each step in the process should be thoroughly evaluated. These air emissions can arise from by-products of chemical reactions, volatile solvents, fugitive leaks, and unreacted materials. While by-products are easily detectable through reaction equations, other types of emissions are not as easily recognizable or quantifiable. Estimating fugitive emissions from equipment or facilities involves complex equations or models, requiring detailed inputs.

To address these emissions, treatment options such as wet scrubbers, regenerative thermal oxidizers (RTOs), and adsorption systems can effectively reduce pollutants by converting them into less harmful substances. Given the lack of onsite air treatment modules in the LCI database for the chemical industry, simplified emission treatment system was design to assess the impacts of estimated emissions an integrated design of a wet scrubber combined with an RTO or catalytic thermal oxidizer (CTO) as the baseline for this study (see Figure S5). This approach represents typical treatment practices, considering energy efficiency and the range of pollutants emitted in this sector.[85] Assumptions regarding inventory data for the oxidizers and scrubber are presented in Tables S12. Average equipment specifications and performance data, representing a medium-scale

treatment facility for the pharmaceutical or chemical industry, were used for modelling. The main sources of impact for the considered system are the natural gas needed for the operation of the combustion chamber, electricity, and gaseous emissions, including CO_2, water, and residual volatile organic compounds. However, the quantification of CO_2, residual volatile organic compounds and other pollutants released required to be quantified based on assumed removal rates of the equipment.

16.1 Estimation of air emissions to treat.

Air emissions in the pharmaceutical and chemical industries can be categorized into three main sources: gaseous by-products, fugitive emissions, and vaporized solvents from manufacturing processes. As a rule of thumb stated by [86], if the amount of vaporized solvents emitted from non-distillation processes, such as dryers, is relatively low, it can be practical and effective to treat these vapours in a RTO as shown in Figure S5, This approach is particularly applicable when these solvents differ from those emitted during distillation. Gaseous by-products are more straightforward to detect and quantify through reaction equations using stoichiometric ratios. However, due to limited data, fugitive emissions require estimation based on available methods, as outlined in the Table S17.

There are several other techniques available for quantifying fugitive emissions, which can be chosen based on the level of accessible data:

- Based on the level of detail of the plant design: Three methods are presented for fugitive emissions estimation during the process design. They are tailored to data available in simple process flow diagram (PFD) to detailed piping & instrumentation diagram. This offers wider applications since the estimation is possible to be conducted even with a basic process data. Precalculated modules represented processes such as distillation, reactor refer to a set of fugitive emission rates data that has been precalculated for standard module types in a chemical process [87].
- Methodology: Methodology utilizing computer-aided process simulation to generate more accurate chemical process LCIs. This methodology accounts for uncontrolled process vent and storage emissions, as well as fugitive emissions.[88]
- U.S. Environmental Protection Agency (U.S. EPA) Techniques: The U.S. EPA has developed emission estimation techniques for point sources in an organized manner. These techniques

provide concise example calculations for identified process operations, storage tanks, equipment leaks, wastewater collection and treatment, cleaning, solvent recovery, and spills as potential air emission sources (EIIP, 2007).

16.2 Designed air pollutant treatment system

As in Figure S5, the systems are integrated such that the output of the wet scrubber feeds into the inlet of the RTO. This ensures that the gas stream entering the RTO is already significantly treated (except for VOCs/volatile solvents), reducing the thermal load on the RTO and optimizing its performance. It was also assumed that the wet scrubbers effectively treated the particulate matter, acid gases and ammonia, where RTO is only effectively treated VOCs/volatile solvents. Continuous operations under same gas flow rate were assumed for all oxidizers and scrubber is investigated in this study. This is because discontinuous operations require additional auxiliary fuel to heat up the combustion chamber at the beginning of each working shift.

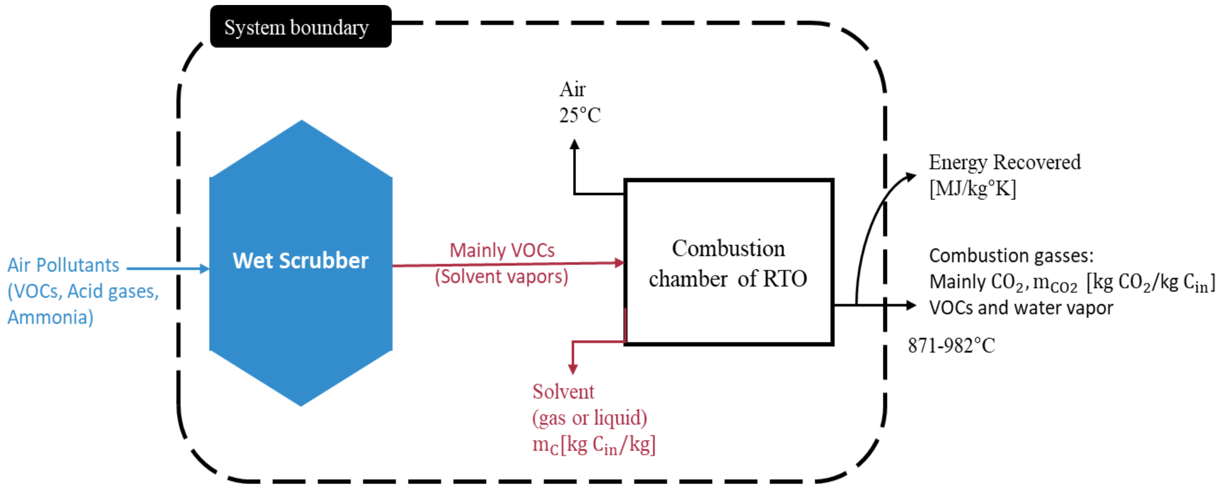

Figure S5. Schematic of the waste gas treatment model (air pollutants treatment).

Average values that could represent pharmaceutical and chemical industrial sources of emissions, were selected for both gas flow and VOC concentrations. Therefore, an inlet gas flow rate of 20000 m^3/h and a VOC concentration of 1000 mg/m^3 were selected.[89] The model waste gas composition was 35.6 vol% benzene, 0.2 vol% toluene, 8.4 vol% ethylbenzene and 55.8 vol% xylene.[90,91] Operational parameters required to model the wet scrubber were taken from the study conducted by Gao et al.[92] Based on these derived values, it is possible to evaluate the quantity of natural gas consumed according to equations given in in for oxidizers and Eve for wet scrubbers

(Table S12). Comparative study on different catalyst conducted by Tomatis et al.[65] clearly demonstrated that Mn-Co catalysts might represent a more sustainable alternative to Cr based catalysts, and therefore, Mn-Co catalyst was selected for this study.

Table S12. Assumptions regarding the energy options for the three equipment.

Equipment	Energy Recovery (%)	Total Energy Input (MW)	Fuel Energy Input (MW)	Pressure Drop (kPa)	Fan and Motor Energy Efficiency (%)
RTO	95	15.0	0.75	4.5	65
CTO	35	12.1	1.0	3.0	65
WS	-	10.2	7.0	1.0	60

16.3 Analysis of the Life Cycle Inventory

Table S13 shows the life cycle inventory of the selected treatment system, which includes the resources necessary for the daily hourly operation based on earlier calculations.

Table S13. LCI data required to model the treatment system.

Category	Item	WS	RTO	CTO	Units
Products					
	Total gas flow<br>VOCs concentration	2,085,484	2,042,148	2,040,253	m^3/day
Inputs from Technosphere (materials & fuels)					
	Natural Gas	46,671	3,335	1,440	m^3
Electricity/heat					
	Electricity	2.3	11.1	5.9	MWh
Emissions to air					
	Pollutant A<br>to pollutant 'n'	NC	NC	NC	kg

[NC] Need to be calculated accordingly

Since the carbon dioxide emitted from RTO is dependent of the formula of the compound combusted, total mass of volatile solvents is therefore not an appropriate basis in order to obtain a generalized model. Assuming complete combustion, the amount of carbon dioxide produced can be calculated directly using stoichiometric relations, the efficiency of the oxidizer (95%), and the mass of carbon entering the oxidizer are shown in.

$$CO_2\ emission = m_c \cdot (\frac{44kg\ of\ CO_2}{12kg\ of\ C}) \cdot \eta$$

Where m_c is the mass of carbon contained in the waste gas stream, and η is the efficiency of the incinerator. If significant sulfur and nitrogen are present, then a direct conversion to SO_x and NO_x can be calculated.

On the other hand, the consumption of auxiliary fuel also influenced CO_2 and water emissions although the same amount of pollutants was processed in different systems. Accordingly, the quantity of CO_2 emitted per hour were 121.0, 51.0 and 53.3 t/day for RTO and CTO, respectively. Due to the incomplete VOCs and pollutant removal, about 5% of the initial amount of pollutants from both web scrubber and oxidizers is still released into the atmosphere after treatment. This released gases to atmosphere required to be quantified accordingly using input masses and included in the Table S13. The VOCs ratio of the different compounds in the effluent stream containing the products of combustion was assumed to be the same as in the input gas.

17. WASTEWATER TREATMENT

Many chemical and pharmaceutical production plants have on-site systems to collect and pre-treat wastewater before it is released to a centralized wastewater treatment plant (WWTP) or directly to waterways.[93] Various treatment methods are used depending on the type and quantity of contamination. Modelling treatment systems for pharmaceutical industries is important due to the different treatment options and their significant impact on the environmental footprint of wastewater treatment in industrial setups.

As a simplification, conventional WWTP designs were considered under the generic design based on functional similarity supplemented with advanced combined treatment options to address specific APIs as required. This approach is necessary because conventional systems have lower removal rates for APIs, which often remain at high levels as residuals in industrial wastewater.[94] Suitable treatment options should be selected based on previous studies conducted with corresponding energy usages and additional material flows referenced from the literature (see Table S14). If industrial data for similar processes are available, they can be used to simulate the on-site wastewater treatment process. LCI inputs for modelling the wastewater treatment system can be based on the study conducted by Li et al.[84], which presents a generalised model for pharmaceutical industrial wastewater under three different treatment scenarios (see Table S15). Where direct energy consumption data (e.g., in kWh/m^3) were not reported, values were estimated based on available experimental parameters such as voltage, current, treatment time, or reagent dose. These estimates were derived using standard energy calculation formulas or benchmarked against values reported for similar systems in the literature. As a guideline, Figure S7 can be used to quantify the wastewater in m^3 and sludge generated per batch during the production process.

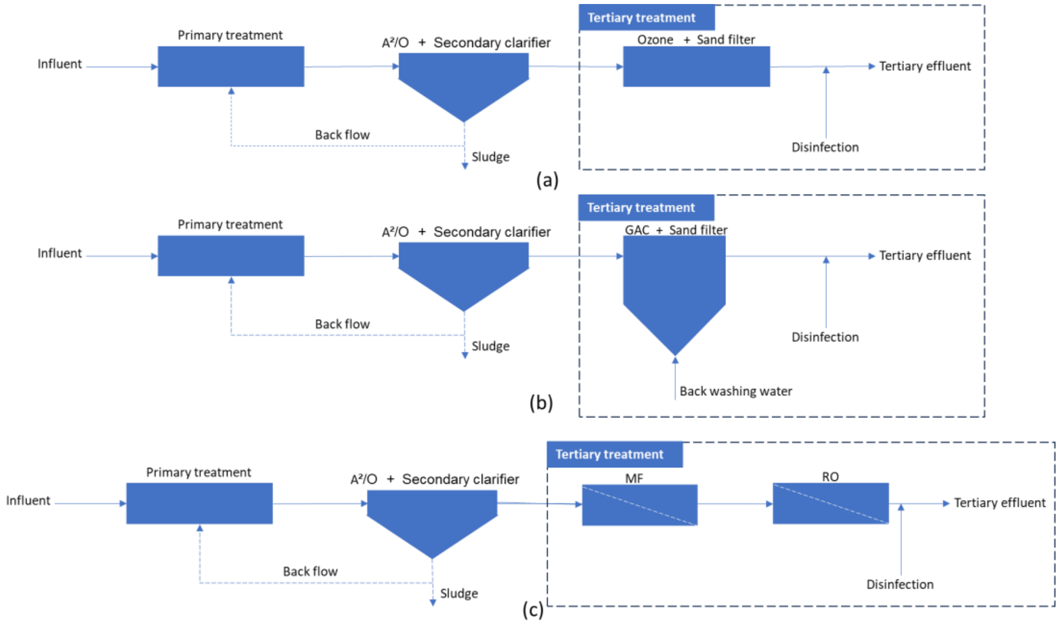

Figure S6. Schematization of the three advanced wastewater treatment processes. (a), (b), and (c) refer to scenario A, B, and C. [84]

The schematic diagrams of three wastewater treatment scenarios with different secondary wastewater treatment processes are showed in Figure S6. **Scenario A** uses a conventional treatment

sequence with primary sedimentation and an A^2/O activated sludge process, followed by ozonation, sand filtration, and disinfection. Ozone is generated from dried air and applied via a reactor system with recirculation and gas-phase monitoring. **Scenario B** includes the same primary and secondary treatments but replaces ozonation with granular activated carbon (GAC) filtration. GAC removes contaminants mainly through adsorption, with operational inputs including fresh media, regeneration cycles, and wash water. **Scenario C** builds on conventional treatment by adding microfiltration (MF) and reverse osmosis (RO) after coagulation and sedimentation. Membrane fouling is controlled by backflushing and chemical cleaning, using acids and bases specific to each membrane type, followed by disinfection.

Table S14. Energy and material usage of various tertiary treatment for pharmaceutical contaminant.

Treatment option	Electricity $kWhm^{-3}$	Pharmaceutical class(es)	API examples	Removal Efficiency (%)	Additional Material Inputs	Quantified Material Inputs per m³	Source
Ozonation (O_3) – usually with downstream BAC/sand	0.16-0.226 (typical full-scale)	Broad PPCPs (analgesics, beta-blockers, X-ray contrast media)	Paracetamol, Ibuprofen, Diclofenac, Naproxen; Carbamazepine; Propranolol	Typically ≥80% for a broad set of micropollutants at ~0.5–0.9 g O_3/g DOC	Ozone (O_3) gas; oxygen supply; quenching/filtration downstream	Specific dose 0.4–0.7 g O_3/g DOC (2–5 g O_3/m³	[95,96,97]
Activated carbon (PAC/GAC) polishing	PAC 0.2; GAC 0.1–0.5 (on-site)	Broad PPCPs	Diclofenac, Carbamazepine, Propranolol, Ibuprofen	Often 70–95% for many PhACs	GAC media (fixed-bed) + backwash water (periodic)	Not specified – no numeric data provided in source	[95]
UV/H_2O_2 (AOP)	3.3 (Mousel 2021); 1.8–8.7 (Hansen 2012, bench-scale); 0.9–1.5 (urban), 7.3–9.1 (hospital/industrial)	Analgesics / anti-inflammatories; broad PPCPs	Paracetamol, Ibuprofen, Diclofenac, Naproxen (plus mixed suites)	Often >80–95% for DCF/SMX/CBZ at adequate UV dose; matrix dependent	Hydrogen peroxide (H_2O_2), UV lamps	H_2O_2 :10–50 mg/L; UV dose 3,000–10,000 J/m²	[95,97,98,99]
Electro-oxidation (BDD)	1.8–4.1 (lab/pilot); up to approximate 12 under extreme settings	NSAIDs & other PPCPs	Diclofenac (model), acetylsalicylic acid; mixtures	High removal/mineralization of PhACs; performance depends on charge density	Supporting electrolyte (e.g., Na_2SO_4 50–100 mM)	Electrolyte 7–14 g/L	[100,101]
Electrocoagulation	0.7–5 (typical); 0.2 to >10 reported	β-lactams, antiepileptics, NSAIDs (in mixtures)	Amoxicillin, Carbamazepine, Diclofenac	Often 70–95% for COD/color; 60–90% for several PhACs (matrix-dependent)	Sacrificial Al anode (dissolved in situ)	Aluminum electrodes (sacrificial): 0.6–1.0 kg/m³ (based on current density and duration)	[102,103]

Ionizing radiation	0.278 -1.39 (estimated value from kGy)	Antibiotics; ARGs reduction	Erythromycin; Sulfamethoxazole; Tetracycline	ERY 80–90%+; SMX ~80%; ARGs 1–1.3 log reduction; higher with PMS/ H_2O_2	Optional PMS or H_2O_2; high-dose radiation	Dose 25–100 kGy; PMS 1–50 mM (study-specific)	[104]

Table S15. Life cycle inventory per 1 m^3 of secondary-treated wastewater for the three types of treatment scenarios (only operational).

Input/Output	O_3	GAC	RO
**Input**			
Water (m^3)	1.00E+00	1.00E+00	1.00E+00
COD_{Cr} (kg)	3.64E-02	3.64E-02	3.50E-02
BOD_5 (kg)	2.24E-02	2.24E-02	2.46E-03
Total suspended solids (kg)	1.48E-02	1.52E-02	1.52E-02
Total nitrogen (kg)	2.25E-02	2.25E-02	2.50E-02
Total phosphorus (kg)	1.30E-03	1.30E-03	1.45E-03
NH_3-N (kg)	1.54E-02	1.54E-02	1.60E-02
Phenol (kg)	1.00E-03	1.00E-03	1.00E-03
Fe^{2+} (kg)	1.60E-04	1.39E-04	1.39E-04
Al^{3+} (kg)	1.40E-05	2.20E-05	2.20E-05
Cu^{2+} (kg)	1.06E-05	1.60E-05	1.60E-05
Mn^{2+} (kg)	9.60E-05	2.50E-05	2.50E-05
Mg^{2+} (kg)	1.01E-02	9.17E-03	9.17E-03
Zn^{2+} (kg)	7.50E-05	1.24E-05	7.40E-05
Cr^{6+} (kg)	5.00E-04	5.00E-04	5.00E-04
Cd^{2+} (kg)	1.00E-04	1.00E-04	1.00E-04
Pb^{2+} (kg)	1.00E-03	1.00E-03	1.00E-03
As^{3+} (kg)	5.00E-04	5.00E-04	5.00E-04
Hg^{2+} (kg)	5.00E-05	5.00E-05	5.00E-05
Electricity (kwh)	5.79E-01	2.31E-01	1.19E+00
Membrane (kg)	n/a [a]	n/a	5.78E-05
Quartz sand (kg)	7.77E-07	6.27E-02	3.31E-03
Liquid chlorine (kg)	3.00E-03	3.00E-03	3.00E-03
Poly aluminium chloride (kg)	n/a	6.00E-02	n/a
Antisludging agent (kg)	n/a	n/a	3.00E-03
$NaHSO_3$(kg)	n/a	n/a	3.00E-03
Isothiazolinone (kg)	n/a	n/a	2.00E-03
NaOH (kg)	n/a	n/a	9.26E-05
HCl (kg)	n/a	n/a	1.85E-04
$FeCl_3$ (kg)	n/a	n/a	2.20E-02
Polyacrylamide (kg)	n/a	5.00E-04	5.50E-02
Polymeric sulphate (kg)	n/a	n/a	4.00E-03
$NaClO_2$ (kg)	n/a	n/a	4.00E-03
GAC (kg)	n/a	1.50E-02	n/a

Wash water (kg)	5.16E-04	5.45E-04	1.44E-04
Ozone (kg)	5.70E-03	n/a	n/a
**Output**			
Water (m3)	1.00E+00	1.00E+00	1.00E+00
CODCr (kg)	2.03E-02	2.67E-02	2.50E-02
BOD5 (kg)	1.94E-03	1.85E-03	1.90E-03
Total suspended solids (kg)	6.70E-03	7.00E-03	7.31E-03
Total nitrogen (kg)	1.17E-02	1.30E-02	1.35E-02
Total phosphorus (kg)	2.80E-04	2.60E-04	2.70E-04
NH3-N (kg)	4.20E-04	8.00E-03	1.19E-03
Cr6+ (kg)	5.00E-05	5.00E-05	5.00E-05
Cd2+ (kg)	1.00E-05	1.00E-05	1.00E-05
Pb2+ (kg)	1.00E-04	1.00E-04	1.00E-04
As3+ (kg)	1.00E-04	1.00E-04	1.00E-04
Hg2+ (kg)	1.00E-06	1.00E-06	1.00E-06
Phenol (kg)	5.00E-04	5.00E-04	5.00E-04
Quartz sand (kg)	6.69E-07	5.65E-02	2.98E-03
Wastewater (kg)	5.16E-04	5.45E-04	1.44E-04
Sludge (kg)	1.78E-01	2.60E-01	1.25E-01
GAC (kg)	n/a	3.00E-03	n/a
Carbon dioxide (kg)	7.24E-03	5.05E-03	1.01E-02
Methane (kg)	3.13E-05	2.40E-04	2.20E-05
Nitrous oxide (kg)	1.73E-06	1.44E-06	2.94E-06

To model the treatment of paracetamol-containing pharmaceutical wastewater is considered an example and conventional wastewater treatment processes were adapted from the Table S15. The baseline system includes secondary treatment. To represent the advanced treatment required for paracetamol removal, a UV/H_2O_2 was overlaid onto the base model. Energy use for this step (approx. 3.3–9.1 kWh/m^3) was added to the inventory, along with specific chemical inputs including hydrogen peroxide (50 mg/L), iron salts (5–10 mg/L), and acid/base reagents for pH adjustment. Sludge generation (0.6–0.8 kg dry solids/m^3) was routed to incineration using ecoinvent's sludge disposal modules. This hybrid approach preserves the functional structure of generic wastewater models while enabling inclusion of API-specific treatment features. All the input details are given in the Table S16.

Table S16. Inventory input details for the example case-Paracetamol.

Category	Input Details
Functional Unit	1 m^3 of paracetamol-containing pharmaceutical wastewater
Influent Characteristics	Paracetamol: 20 mg/L; COD: 3200 mg/L; pH 6.5
Target Removal Efficiency	Paracetamol: 99.8%; COD: 90% removal
Treatment Technology	UV/H_2O_2, pH control
Electricity Consumption	3.3–9.1 kWh/m^3 (UV/H_2O_2); add 0.25–0.45 (secondary) + 0.04–0.10 (primary) + 0.03–0.10 (disinfection) from Table S10 if modeled in series
Chemical Inputs	H_2O_2: 50 mg/L; Fe^{2+}: 5–10 mg/L; Acid/base for pH adjustment (HCl/NaOH)
Sludge Production	0.6–0.8 kg dry solids/m^3 estimated from COD reduction
Output Emissions/Residuals	Negligible residual paracetamol; sludge directed to incineration
Conventional WWTP Stage Integration	Incorporate general WWTP stages from ecoinvent v3.x: secondary treatment (CAS), tertiary polishing, disinfection, sludge treatment
Background System References	ecoinvent v3.9: EU electricity mix, H_2O_2 production, iron salt, sludge incineration, chemical pH neutralization

Estimation of the wastewater and sludge generated per batch

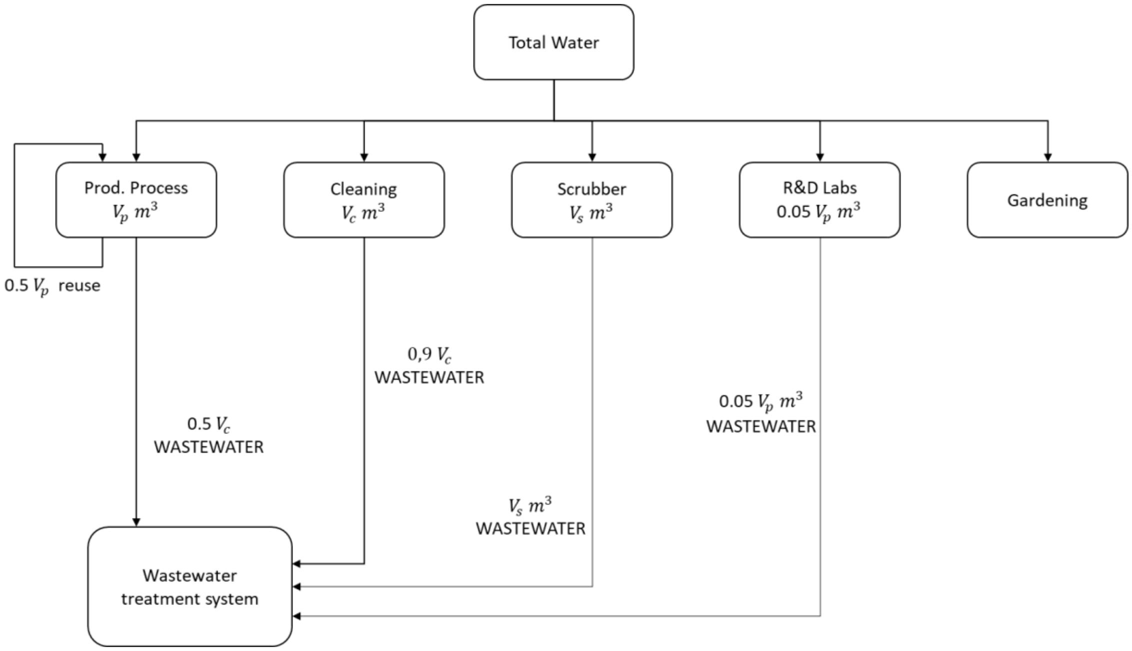

Figure S7. Water balance for generic pharmaceutical plant with chemical synthesis process.

To estimate the wastewater and sludge generated per batch during the pharmaceutical production process, framework presented in the figure S7 can be used. This approach is developed based on the case study given in the.[105]

Production Process Water (V_p): The volume of water used in the production process, denoted as V_p, can be calculated from the specific steps involved in producing the pharmaceutical products. Each step's water consumption should be documented and summed to determine $V_{p.}$ The wastewater generated from the production process is assumed to be 50% of the water used.

Cleaning Water (V_c): The volume of water used for cleaning equipment is denoted as V_c. This can be determined from the calculations for cleaning equipment under the Section 3.3.6 in the manuscript. The wastewater generated from cleaning is assumed to be 90% of the cleaning water used.

Scrubber Water (Vs): The volume of water used in scrubbers is denoted as V_s, if this process is included within the plant design. This value is not provided directly and would need to be measured or estimated based on the scrubber's operational parameters. The wastewater generated from scrubbers is assumed to be a percentage of the scrubber water.

R&D Labs Water (0.05Vp): The water used in R&D labs is calculated as a percentage of the production process water. In this case, it is 0.05 V_p. The wastewater generated from R&D labs is assumed to be 100% of the lab water used. Finally, the total wastewater generated can be summed up from all the individual wastewater source.

SECTION 7: SENSITIVITY ANALYSIS

HVAC ALLOCATION ASSUMPTIONS

The allocation of HVAC energy to individual production lines was tested in three scenarios (2, 5, and 10 lines) to reflect variability across pharmaceutical manufacturing facilities.

- Two-line scenario (baseline): We retained 2 lines as the baseline assumption, consistent with several industrial case studies and reports that report dual-line operations in pharmaceutical plants.[106,107] Therefore, in the recommended approach the commonly cited 21% HVAC share is divided equally across two lines, resulting in a 10.5% allocation per line.
- Upper-extreme scenario (2 lines, 30% HVAC share): Several sources report AHU contributions in the range of 20–40% of total site energy.[108] To reflect this variability, we applied a 30% HVAC contribution in a two-line scenario as an upper-extreme case, corresponding to a 15% allocation per line.
- Five-line scenario: Literature sources indicate that 1–5 production lines are common in pharmaceutical facilities.[109] To reflect the upper bound of this reported range, we included a 5-line scenario, which allocates 4.2% of total energy to HVAC per line.
- Ten-line scenario: Certain industry reports note that large facilities can operate more than 10 lines.[110,111] To test this extreme case, we modelled a 10-line scenario, corresponding to a 2.1% HVAC contribution per line.

This tiered allocation approach captures realistic variability across facilities of different scales, while ensuring that our results are not dependent on a single assumption about production line numbers.

SENSITIVITY TO AHU ALLOCATION ASSUMPTIONS

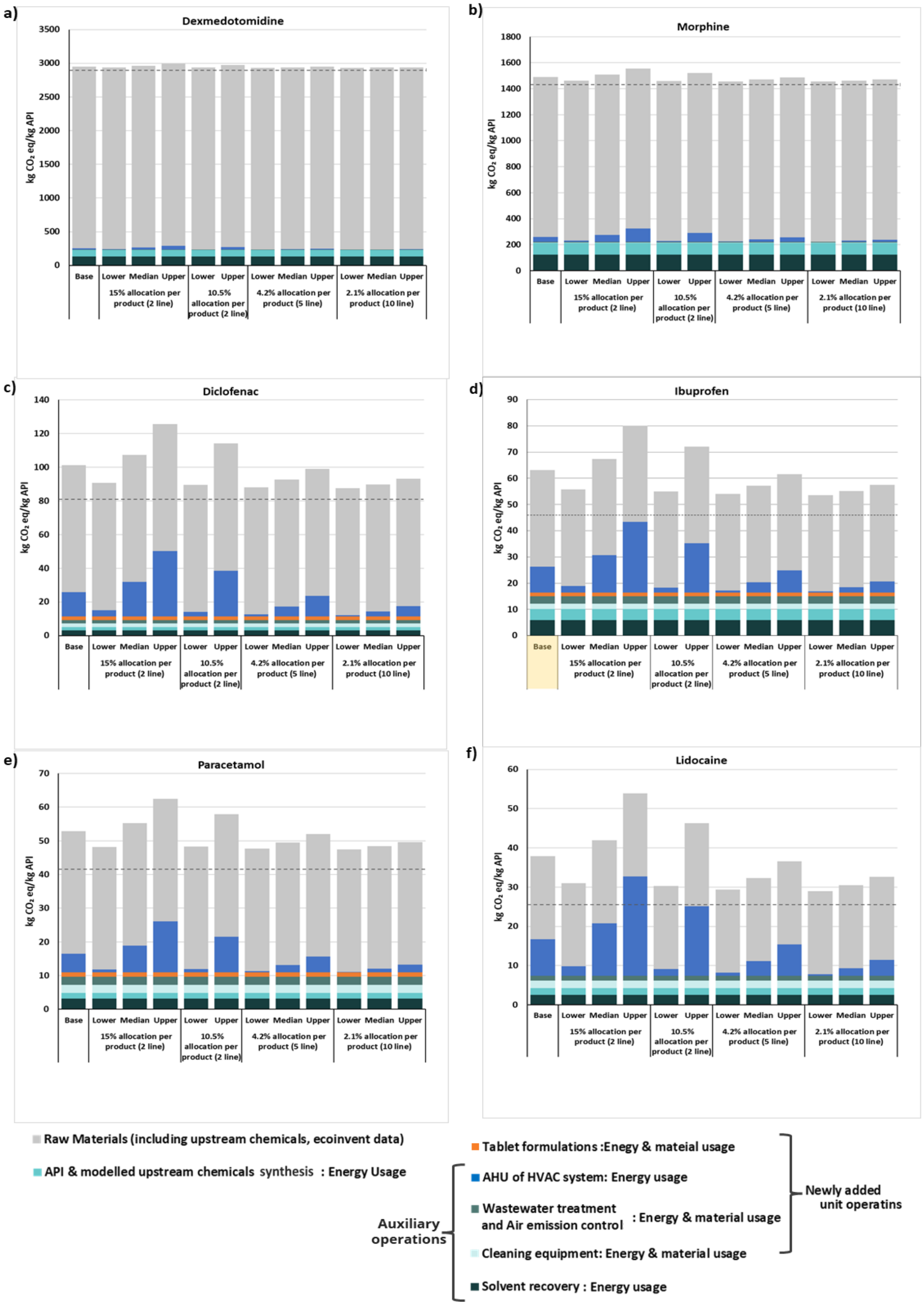

Figure S8. Carbon footprint per kilogram of Ibuprofen and Diclofenac under different HVAC allocation scenarios. Results are based on the 10th, mean, and 90th percentiles of the 68-facility dataset, with HVAC energy shares distributed across two, five, or ten production lines. The dotted line shows the carbon footprint derived with the existing method. (a) Dexmedetomidine. (b)Morphine. (c)Diclofenac. (d)Ibuprofen. (e)Paracetamol. (f)Lidocaine.

SENSITIVITY TO REFERENCE BENCHMARK VALUES

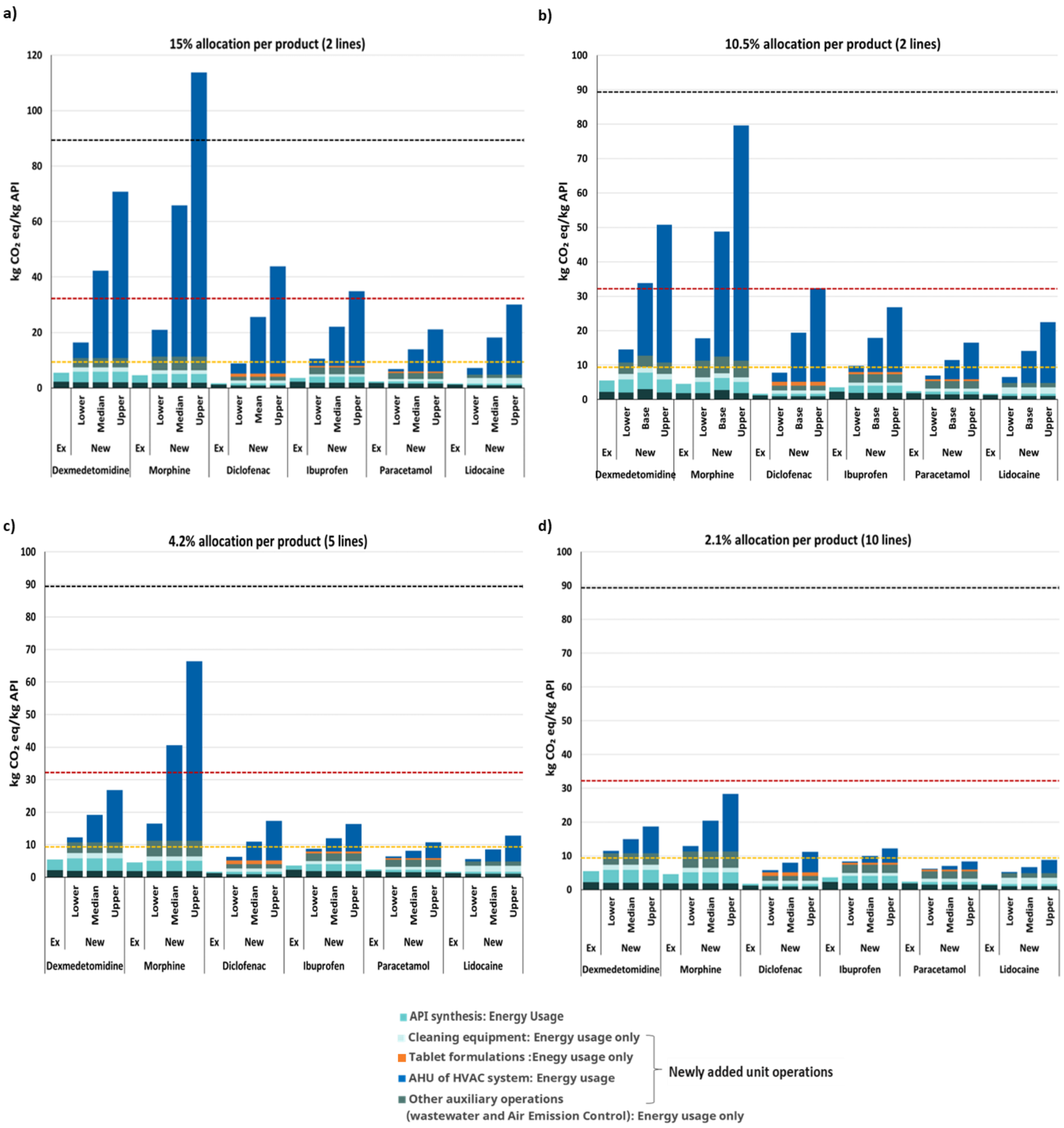

Figure S9. Comparison of carbon footprint per kilogram of six APIs based on foreground energy estimates against from the new and existing methods against percentile-based industrial references. The red dotted line indicates the upper percentile (90th), the red dot line the mean, and the orange line the lower percentile (10th). Per-product HVAC allocations of (a) 15% (2 lines), (b) 10.5% (2 lines), (c) 4.2% (5 lines), (d) 2.1% (10 lines).

SENSITIVITY TO SOLVENT RECOVERY ASSUMPTIONS

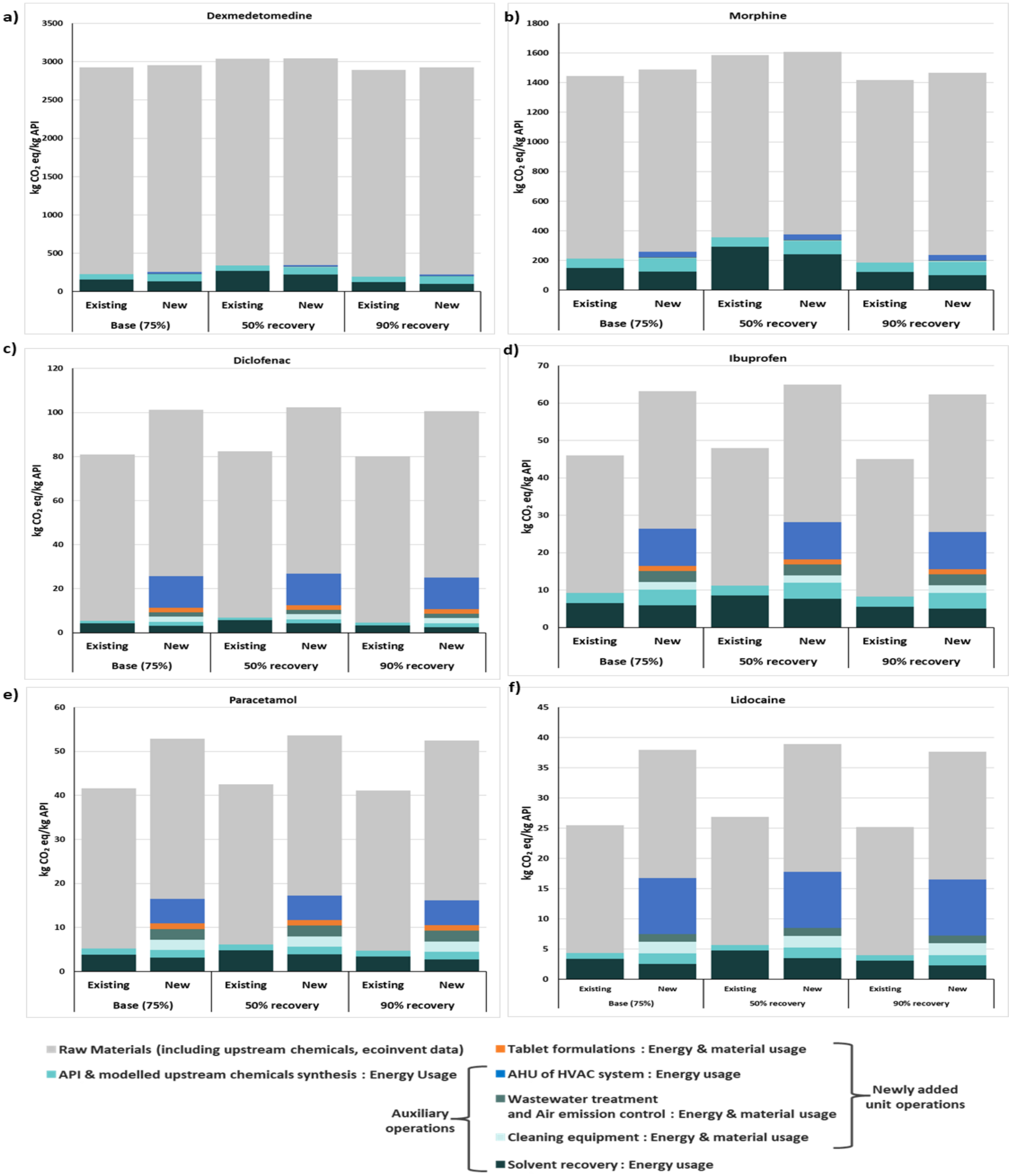

Figure S10. Carbon footprint per kilogram of six APIs under different different solvent recovery percentages under new and existing method. (a) Dexmedetomidine. (b)Morphine. (c)Diclofenac. (d)Ibuprofen. (e)Paracetamol. (f)Lidocaine.

Variations in solvent recovery rates had only a minor effect on the overall carbon footprint compared with the baseline assumption of 75% recovery, with changes remaining below 7%. The main environmental benefit of higher recovery is that less solvent waste is sent to incineration, while the additional energy required for recovery is also included in the assessment. A larger benefit could be expected if the recovered solvents were reused directly in pharmaceutical production, thereby reducing the need for virgin solvents. However, in line with industrial practice reported in the literature,[54,55] recovered solvents are not reused in synthesis due to stringent purity requirements but are instead sold to other industries with lower purity needs.

SECTION 8: CASE STUDY

8.1 Identification of API Synthesis route

This section, summarizes for each API (i) the number of synthesis routes identified in the literature and patents, and (ii) the rationale for the selected route based on the four criteria (industrial relevance, recency/regulatory alignment, data availability, and safety/environmental feasibility). Experts were consulted to identify and validate key selection factors for all these criteria; these factors are listed under each criterion. Regulatory alignment was assessed against standard industrial practice and typical emission-control measures. No local regulations were examined in detail.

1. Ibuprofen

We screened 9 distinct batch routes for ibuprofen, grouped by reaction/mechanism class. After applying the four pre-specified criteria and a brief chemistry expert consultation, we retained three representative routes: BHC,[2] aryl-translocation (ketal/Zn-ibuprofenate), and the Tamura et al.[112] hypervalent iodine rearrangement. Finally, we selected BHC as the representative route for inventory modelling (U.S. Patent 4981995).[2] Routes were excluded mainly for lack of industrial practice and the use of hazardous reagents/solvents.

I. Industrial relevance
- Commercialized in 1992, BHC is a Proven, long-running industrial process. It replaced the older Boots six-step route and remains a benchmark referenced in modern summaries and reviews. [113]
- Recognized with the U.S. Environmental Protection Agency (EPA) Presidential Green Chemistry Challenge Award (1997) for its manufacturing impact.
- Alternatives: the Tamura route is primarily bench-scale, proof-of-concept with no reported commercial adoption; the aryl-1,2 translocation rearrangement route presented in Ma et al.[114] is industrial in China but not globally dominant.

II. Recency & regulatory alignment
- Selected BHC route uses closed-loop HF and catalytic carbonylation; with closed transfers and solvent recovery, it is consistent with standard industry practice (closed HF loop, closed transfers, solvent recovery).
- Long-standing industrial adoption (since 1992) is consistent with good industrial practice; no special controls are required.

III. Data availability of selected route
- Case studies and technical overviews describe the three steps in detail, enabling robust LCI modelling.

- There is sufficient patent and lab data to model the upstream chemicals absent from ecoinvent database, relative to the other synthesis routes.

IV. Safety & environmental feasibility

- Selected route High atom utilization and minimal waste. EPA has stated that in the BHC process, virtually all starting materials are converted to product or recovered/recycled. HF is hazardous but contained in a closed loop and continuously recycled, representing a clear improvement over $AlCl_3$-based acylations.

- Alternative routes such as the Tamura process require stoichiometric $PhI(OAc)_2$ (costly, iodine-containing waste), while the Chinese aryl-translocation route shortens reaction time but still relies on $AlCl_3$ and halogenated solvents, increasing waste-handling needs.

Table S16. Summary of five batch routes screened for ibuprofen and the rationale for selecting US4981995. It compares each route against the four pre-specified criteria and provides a brief reason for exclusion for non-selected routes.

Patent / protocol (year)	Industrial relevance	Recency & regulatory alignment	Data availability	Safety & environmental feasibility	Reason for exclusion
BHC — US4981995 (1991)[2] — Selected	Commercialised since 1992; widely cited as the benchmark and 1997 EPA PGCC award winner. (EPA)	Long-running commercial process operated under standard industry practice.	Sufficient data for LCI estimation.	HF-catalysed steps and Pd/CO carbonylation run in closed recycle; fluoride brine/acidic condensates for treatment, and HF/solvents are recovered.	—
Aryl-1,2 translocation (ketal / Zn-ibuprofenate) — Ma et al., 2018[114] (industrial CN practice)	Established domestically in China; paper reports plant-style bottlenecks and implemented fixes.	Consistent with standard industry practice (batch equipment, closed handling, solvent recovery)	Provides stepwise process and quantified upgrades, enabling LCI with few assumptions.	HCl off-gas and VOCs mitigated via wet scrubbing; aqueous workups give chloride/neutralisation salts.	Strong industrial relevance in China, but no global dominance/ higher VOC management burden from different stages.
Tamura et al. (1985)[112] hypervalent-iodine rearrangement (PIDA/orthoester)	Bench-scale academic route; no evidence of commercial adoption.	Permit-compatible under standard industry practice where applied, but not widely industrialised.	Limited operating data for LCI (no stepwise mass/energy balances).	Organic solvents for recovery; overall higher oxidant/salt waste than the selected route.	Proof-of-concept only; carries higher reagent/waste burden than BHC.

2. Diclofenac

We screened five batch routes, grouped by reaction/mechanism class. Applying the four pre-specified criteria, we selected patent CN113402408 (2021)[115] as the representative route for inventory modelling. This route is recent, industrially oriented with acceptable Environment, Health, and Safety (EHS) profile. Others were excluded due to legacy practice, hazardous media, or insufficient operating data for LCI.

I. Industrial relevance

- Patent CN113402408[115] is designed for plant implementation; it provide a complete sequence of step sequence with operating conditions and overall yield/purity.
- Early Ullmann variants (US20200055811) and Alicyclic-aromatization routes (CN1242984C) are longer, include steps that are less common in current GMPs, and are not supported by pilot-plant style examples.; EP0380712 is industrially oriented but dated; some modern variants are less well established at industrial scale (US10662145-US20200055811).

II. Recency & regulatory alignment

- Selected route avoids mixed-acid nitration and $SOCl_2$ and uses standard solvents/Lewis acid; with wet scrubbing and closed transfers, it is consistent with standard industrial practice (closed transfers, wet scrubbing, solvent recovery).
- Alternatives (EP0380712; nitration/$SOCl_2$; cyclohexanone/isatin) are more control-intensive and require tighter permitting and operational monitoring relative to the selected route.

III. Data availability

- The selected patent provides quantitative reagent ratios, temperature/time ranges, step yields, overall yield (80%) and final purity (99.8%) → directly usable for LCI, least assumptions-based gap filling.
- There is sufficient patent and lab data to model the upstream chemicals that are not covered in the ecoinvent database relative to the other synthesis routes.

IV. Safety & environmental feasibility

- Hazards ($AlCl_3$, chloroacetyl chloride, HCl) are standard and manageable with closed transfers, scrubbing, and neutralisation; no exotic or highly regulated reagents. The route does not require mixed-acid nitration, $SOCl_2$, or chlorine gas, which typically create additional permitting and abatement burdens.

Table S17. Summary of five batch routes screened for diclofenac and the rationale for selecting CN113402408A (2021). It compares each route against the four pre-specified criteria and provides a brief reason for exclusion for non-selected routes.

Patent / protocol (year)	Industrial relevance	Recency & regulatory alignment	Data availability	Safety & environmental feasibility	Reason for exclusion

CN113402408A (2021)[115] — Selected	Described as suitable for industrial production, with conventional batch operations	Avoids nitration, $SOCl_2$, and Cl_2; consistent with standard industry practice (closed transfers, HCl scrubbing, solvent recovery).	Gives full reagent ratios, work-up steps, step yields. Sufficient data for LCI estimation.	HCL off gases, Al-salts/aqueous phases are routinely managed and solvents are recovered.	—
EP0380712B1[116] (1990)	Designed for manufacture; large-scale examples with isolated yields.	Fits standard industry practice with HCl scrubbing and solvent recovery; no nitration/$SOCl_2$/Cl_2.	Sufficient data for LCI estimation	Hazards (acid chloride, $AlCl_3$, chlorinated solvents, strong base) are controllable but reflect older solvent choices.	Dated relative to CN113402408A.
US10662145[117] / US20200055811 (2020)[118]	Scalable on paper; longer sequence; specialty reagents.	Includes mixed-acid nitration and $SOCl_2$ will increase regulatory load and controls.	Sufficient data for LCI estimation	Chemical hazards (mixed-acid nitration, $SOCl_2$) requires tighter off-gases controls compared to selected route.	Higher regulatory/EHS burden (nitration, $SOCl_2$) and not clearly demonstrated at commercial scale.
CN1242984C[119] / CN1580039A (2005–2006)[120]	Conceptual; uncommon in current GMP practice; no pilot-style examples.	Chlorination/aromatisation sequence with likely Cl_2 handling; implementable but heavier compliance.	Limited per-step operating parameters and no detailed mass balance.	HCl off-gas and chlorinated waste streams that require gas containment/scrubbers	Limited reproducible operating detail and relies on harsher chlorination/aromatisation; not preferred for LCI.
CN113429308B (2023)[121]	Framed as industrially simplified; multi-mol lab examples with HPLC data.	Align with standard industry practice under closed handling; no $SOCl_2$/Cl_2.	Sufficient data for LCI estimation	NO_x/acid management and HCl off-gas capture during acid-chloride steps. Both are controllable.	Recent and data-rich, but either still uses nitration or is not clearly more advantageous than selected route on EHS or completeness.

3. Paracetamol

We screened six batch routes, grouped by reaction/mechanism class. Applying four pre-specified criteria, we selected the phenol nitration → p-nitrophenol (PNP) → p-aminophenol (PAP)→ paracetamol route for inventory modelling[122-125]. This route is industrially established, well-documented for LCI, and manageable under current EHS controls. We anchor each stage to representative patents (nitration, p/o separation, Fe/HCl reduction, acetylation), and note that recent continuous implementations use the same three-step chemistry.

- The sequence in this route serves as a stepwise, well-documented batch benchmark enabling transparent unit-operation mapping.
- Each transformation corresponds to standard unit operations (acid nitration, hydrogenation, acetylation) and uses commodity reagents available worldwide.

- Alternative routes such as nitrobenzene and PNCB hydrolysis are also practiced but are either more technically demanding or less transparently documented at plant level.

I. Recency and regulatory alignment
- Mixed-acid nitration (HNO_3/H_2SO_4), Fe/HCl or H_2 reduction, and acetic anhydride/acetic acid handling are regulated but widely permitted under current industrial and occupational standards.
- It avoids nitrobenzene acid-phase hydrogenation (and the associated aniline co-product control) and avoids chlorinated feedstocks required for PNCB routes; it does not require high-temperature caustic fusion.

II. Data availability
- Extensive open-literature data exist for each steps with the patents: reagent ratios, temperatures, isolation methods, and product yields.
- Both Fe/HCl and H_2-catalysed reductions are described quantitatively, enabling LCI construction without tentative assumptions.

III. Safety and environmental feasibility
- Manageable known hazards: nitration (NO_x control, acid recovery), reduction (H_2 handling or Fe-sludge neutralisation/filtration for Fe/HCl), and acetylation (closed Ac_2O handling, exotherm control).
- Choosing the catalytic H_2 variant minimises $Fe(OH)_x$ waste compared with Fe/HCl.
- By contrast, PNCB routes involve chlorinated intermediates/effluent, and nitrobenzene routes involve strongly acidic hydrogenation with aniline co-product—both typically demand more complex abatement/control than the selected sequence.

Table S18. Summary of the five batch routes screened for diclofenac and the rationale for selected route. It compares each route against the four pre-specified criteria and provides a brief reason for exclusion for non-selected routes.

Patent / protocol (year)	Industrial relevance	Recency & regulatory alignment	Data availability	Safety & environmental feasibility	Reason for exclusion
Phenol → PNP → PAP → Paracetamol — Selected *US3668261A nitration;*[122] *US3954892A work-up;*[123] *DE2930754C2 Fe/HCl reduction;*[124]	Widely practiced batch sequence.	Mixed-acid nitration, Fe/HCl (or H_2) reduction, and acetylation are regulated but routinely permitted with controls.	Stepwise operating detail is broadly available: enabling LCI with minimal assumptions.	Known hazards management: NO_x/acid scrubbing (nitration), iron-sludge neutralisation/filtration for Fe/HCl and controlled Ac_2O addition.	—

US3917695A acetylation;[125]					
Nitrobenzene → Paracetamol — CN104628592B/A (2015)[126]	Promising integrated concept; not yet documented as a global production standard.	Avoids nitration; acceptable under standard industry practice with closed H_2 and Ac_2O handling.	Sufficient data for LCI estimation	Concentrates hazards (H_2 + Ac_2O + AcOH) in one operation; introduces Zn-salt effluent treatment with with AcOH/Ac_2O recovery.	Avoids nitration but limited evidence of broad adoption.
PNCB route — US4264525 (1981);[127] hydrolysis anchor: US3283011 (1966)[128]	Full stepwise chain described.	Practicable but relies on chlorinated feedstocks; compliance load higher than selected route.	Sufficient data for LCI estimation.	Chloride-rich streams and chlorinated intermediates increase effluent-treatment burden.	Heavier chloride/acid effluent footprint without a clear data. No clear EHS or data advantage.
PNP hydrogenation + acetylation — US4670589 (1987)[129]	Industrially relevant integration of hydrogenation and acetylation to simplify equipment trains.	Permittable under standard industry practice.	Sufficient data for LCI estimation	Concentrates hazards (H_2 + Ac_2O + acid) in one vessel, increasing operational criticality. Acetate liquor for recovery.	Attractive integration but less representative of widely used; no clear EHS advantage.
Beckmann (4-HAP oxime → APAP) — US5155273 (1992)[130]	Representative Beckmann variant with improvements.	Implementable with standard industry practice for corrosive media.	Limited per-step operating parameters.	Strong-acid/oxime handling and salt-laden effluents treatment; typically require corrosion-resistant equipment.5	Less transparently documented and introduces strong-acid/oxime handling without a clear advantage.
PPA (phenol → 4-HAP → oxime → Beckmann → APAP) — CN101298425B[131]	Primarily patent-level disclosure rather than widely reported plant practice.	Strong-acid (PPA) concept within standard industry practice for corrosives.	Sufficient data for LCI estimation	Concentrated strong-acid t and oxime handling; phosphate-rich aqueous waste treatment.	Introduces strong-acid/oxime handling without a clear EHS/data benefit over the selected route.

4. Lidocaine

We screened four batch routes, grouped by reaction/mechanism class. Applying four prespecified criteria, we selected Reiley (1999)[132] as the representative route for inventory modelling. We select the this two-step batch route because it is short, plant-portable, EHS-aligned, and well documented. Routes with longer precursors or alternative couplings were excluded because they increase operability or EHS complexity, or add steps without clear quality or efficiency gains.

I. Industrial relevance
   - The selected route is a widely adopted industry reference. It uses commodity solvents (acetone, n-hexane) and provides high-yield isolations through crystallization with solvent recovery.
   - Alternative routes involve similar chemistry but higher equipment specificity (CN102070483B) or add unnecessary steps without selectivity improvement (CN110938012A).

II. Recency / regulatory alignment
   - Modern implementations avoid added inorganic base and capture/reuse HCl; with closed transfers, wet scrubbing, and solvent recovery, the route is consistent with good industrial practice and typical emission-control measures.
   - Alternatives introduce less-favoured operations (e.g., DCM use, catalytic H_2 reduction) that are more control-intensive.

III. Data availability
   - Reiley (1999) provide explicit stoichiometry, solvent specifications, and unit-operation details, enabling robust LCI parameterization.

IV. Safety / environmental feasibility
   - Chloroacetyl chloride (corrosive, HCl evolution) and diethylamine (volatile, flammable) are handled in closed equipment with controlled addition, heat removal, and vent scrubbing followed by neutralization.
   - No-added-base variants reduce salt formation at quench; solvents are recovered by standard distillation with residuals managed in conventional waste streams.
   - Routes relying on DCM or H_2 reduction increase exposure potential and abatement demand relative to the selected route.

Table S19. Summary of five batch routes screened for diclofenac and the rationale for selecting Reiley (1999). It compares each route against the four pre-specified criteria and provides a brief reason for exclusion for non-selected routes.

Patent / protocol (year)	Industrial relevance	Recency & regulatory alignment	Data availability	Safety & environmental feasibility	Reason for exclusion

Reiley (1999)[132] two-step batch (2,6-dimethylaniline → chloroacetamide → lidocaine) — Selected	Two-step sequence used as an industry/teaching benchmark; directly able to mapped into common unit operations.	Consistent with standard industry practice	Sufficient data f/or LCI estimation7	Low salt profile; solvent residues recovered.	—
Acetone/carbonate, single-vessel (one-pot) option — CN102070483B (2013)[133]	Two-step or single-vessel batch in acetone with carbonate base; industrially oriented.	Conforms to standard industry practice (acetone; batch/one-pot acceptable with validation).	Charges, ratios, temps/times; detailed single-vessel example (overall ~82% yield).	Carbonate scavenges HCl to NaCl/KCl, creating a slurry that needs removal, vent scrubbing, brine treatment and solvent recovery.	adds inorganic salt burden and single-vessel slurry/heat-removal complexity without a clear EHS/quality advantage vs the simpler two-step route.
"No added base" batch variant — WO2021159754A1[134] / CN111253273A (2021/2020)[135]	Batch chloroacetylation without extra alkali, then amination; can be telescoped; plant-portable.	Aligns with standard industry practice (closed HCl capture; recyclable solvents).	Solvent set and qualitative ranges; usable for LCI with modest assumptions.	HCl to wet scrubber; no salt cake; solvent for recovery.	Not selected: attractive (lower salt) but no clear evidence of superior robustness at scale vs the well-documented two-step.
Three-stage with upstream hydrogenation (NB → xylidine) + DCM/hexane — CN110938012A (2020)[136]	Adds Pd/C H_2 reduction upstream; longer train.	Aligns with standard industry practice	Stepwise conditions for reduction, acylation, and amination.	Spent-catalyst filtration; DCM/hexane vapours and HCl off gas to scrubber.	Not selected: extra unit operation and less-favoured solvents, without selectivity/quality gains over the two-step route.

5. Dexmedetomidine

We screened three batch routes, grouped by reaction/mechanism class. Applying four prespecified criteria, we selected asymmetric catalytic route (WO2013069025)[137] as the representative route for inventory modelling. We select this route because it delivers shorter step-count, high step yields, aligns with current EHS and efficiency goals, and provides clean, citable inputs for LCI. The tartaric-acid resolution of racemate (EP4055009) and older racemic syntheses (US4910214) were excluded because they lower overall yield, increase solvent and energy use, and add operations without improving API quality.

Criterion-by-criterion justification

I. Industrial relevance

- The asymmetric route report a short overall sequence with high isolated yields in the key hydrogenation step, supporting industrial throughput. The reagents (aryl bromide, imidazole, Grignard reagents, alcohols) and unit operations (dehydration, hydrogenation, crystallization) are standard in API facilities, and no chromatographic purification is required.
- In contrast, the classical tartaric-acid resolution is throughput-limiting (multiple recrystallizations, low overall yield from the racemate), and older racemic syntheses add steps without a selectivity advantage.

II. Recency / regulatory alignment

- The asymmetric catalytic route directly furnishes the target enantiomer and employs closed, mild-condition hydrogenation; with standard closed transfers and solvent recovery, it is consistent with good industrial practice and typical emission-control measures.
- Resolution-based and older racemic routes are more control-intensive due to additional isolation/recycle operations.

III. Data availability (for LCI)

- The patent provides clearly defined steps (coupling → dehydration → asymmetric hydrogenation) with reported yields, catalyst loadings, and isolation data, enabling straightforward LCI parameterization.

IV. Safety / environmental feasibility

- Hydrogenation (flammability/pressure) is run in closed systems at moderate conditions; metal residues are controlled at ppm-level, and Grignard operations are performed under inert atmosphere with controlled quench.
- The route avoids bulk acid chlorides and heavy-metal oxidants; solvents are recovered by distillation, and routine aqueous/organic waste streams are manageable.
- Other routes generate mother-liquor and recycle loops that raise handling and operational risk compared with the selected route.

Table S19. Summary of the five batch routes screened for diclofenac and the rationale for selecting WO2013069025 (2013). It compares each route against the four pre-specified criteria and provides a brief reason for exclusion for non-selected routes.

Patent / protocol (year)	Industrial relevance	Recency & regulatory alignment	Data availability	Safety & environmental feasibility	Reason for exclusion

Tartaric-acid resolution of racemate — WO2013069025 (2013)[137] — Selected	Practical, plant-portable resolution of medetomidine with staged crystallisations; no chromatography; well known in industry.	Permit-compatible under standard industry practice (closed handling, distillation, solvent recovery).	Full, stepwise procedure — directly usable for LCI.	$NaHCO_3$ free-basing (CO_2, sodium tartrate), and DCM extraction are run in closed systems with vent scrubbing, distillation/solvent recovery, and salt-laden aqueous to wastewater treatment.	—
Asymmetric catalytic route — EP4055009 (2021/2023 B1)[138]	Shorter sequence to the target enantiomer; no resolution loop.	Fits standard industry practice (closed H_2 hydrogenation, solvent recovery).	Clear step order, catalysts, and yields/ee; adequate for LCI.	Grignard and H_2 hydrogenation managed by cooled metered quench; Mg-salt aqueous and catalyst filtrates treated; solvents for recovery.	Less representative than the resolution route; requires chiral H_2 hydrogenation and Grignard handling that many sites don't run;; no clear EHS or compliance advantage
Older racemic synthesis with resolution — US4910214 (1990)[139]	Foundational racemate→resolution disclosure; longer effective step count.	Acceptable under standard industry practice but legacy compared with modern resolution/catalytic options.	Descriptive options for chiral acids and separations; less prescriptive for LCI.	Alcoholic operations and diastereomeric-salt workups controlled by distillation, neutralisation, filtration; mother liquors and inorganic salts require treatment; solvents for recovery.	Lower material efficiency and heavier solvent/salt handling versus the selected route.

6. Morphine

We screened two fully synthetic batch routes, grouped by reaction/mechanism class (patents and peer-reviewed sources). Applying four prespecified criteria, we selected the fully synthetic route from US8293927[140] for inventory modelling. This route uses standard organic operations, offers a stable and modellable supply independent of agriculture. Opium-extraction routes were excluded due to their variable, and agro-intensive nature, while biosynthetic variants were excluded as early-stage and still facing regulatory and yield uncertainties.

I. Industrial relevance

- The selected patent sequence uses established organic transformations typical of batch API facilities and compatible with good manufacturing practices. These represent practical, in some cases explicitly scalable chemistries, confirming industrial applicability of fully synthetic morphine manufacture.

- Independence from agricultural supply: unlike poppy-based production (GB713689)[141], the synthetic route decouples output from licensed cultivation and crop variability, enabling location-flexible manufacturing under normal API regulatory controls.

II. Safety & regulatory alignment
- Synthetic manufacture avoids narcotic raw materials (opium, poppy straw, concentrate of poppy straw), which are restricted under different legislation.
- The selected fully synthetic route is compatible with international drug-control objectives, supporting secure and scalable supply without dependence on licensed cultivation or limited grower nations.

III. Data availability

- The selected patent discloses step sequences, intermediates, and operating parameters sufficient to parameterize mass and energy balances for LCI.
- The opium-based route is difficult to model due to uncertain field yields, regional variability, and lack of bioprocess data, which limits comparability with other APIs.

IV. Safety / environmental feasibility
- Eliminates land, water, and agrochemical burdens associated with opium cultivation. Poppy farming is linked to irrigation demand, pesticide use, and local soil and water contamination; a fully synthetic route avoids these impacts and allows centralized solvent recovery and controlled waste management.

8.2 PROCESS FLOW OF API SYNTHESIS

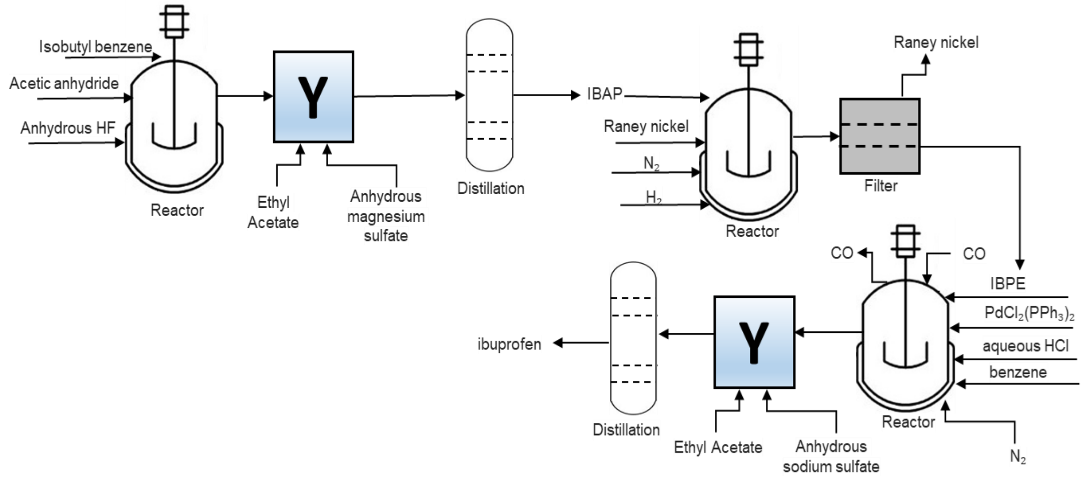

Figure S11. Illustration of ibuprofen synthesis.

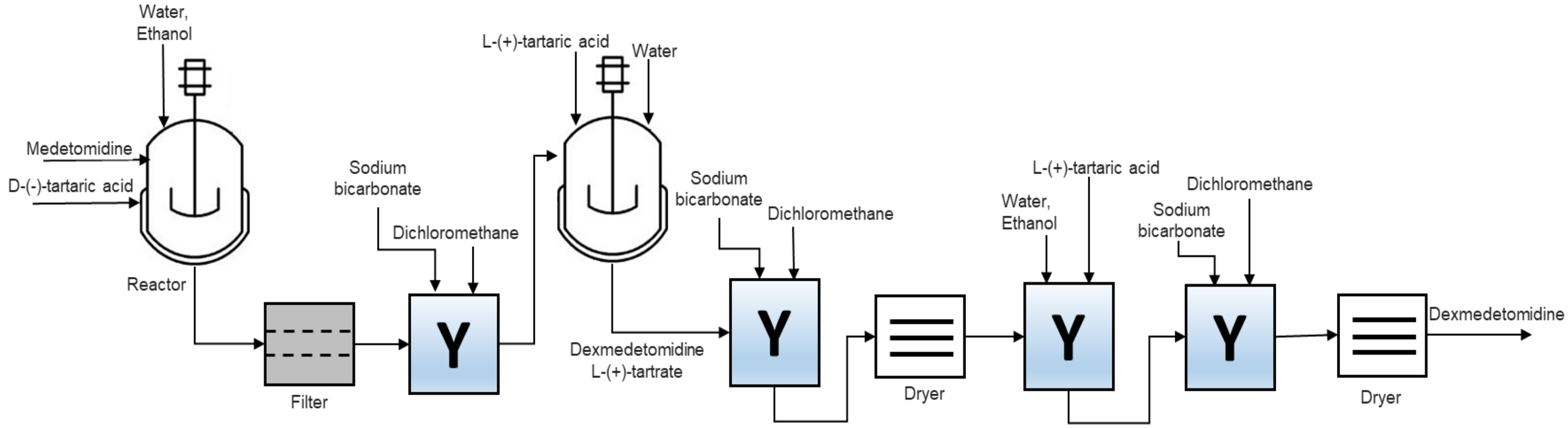

Figure S12. Illustration of dexmedetomidine synthesis.

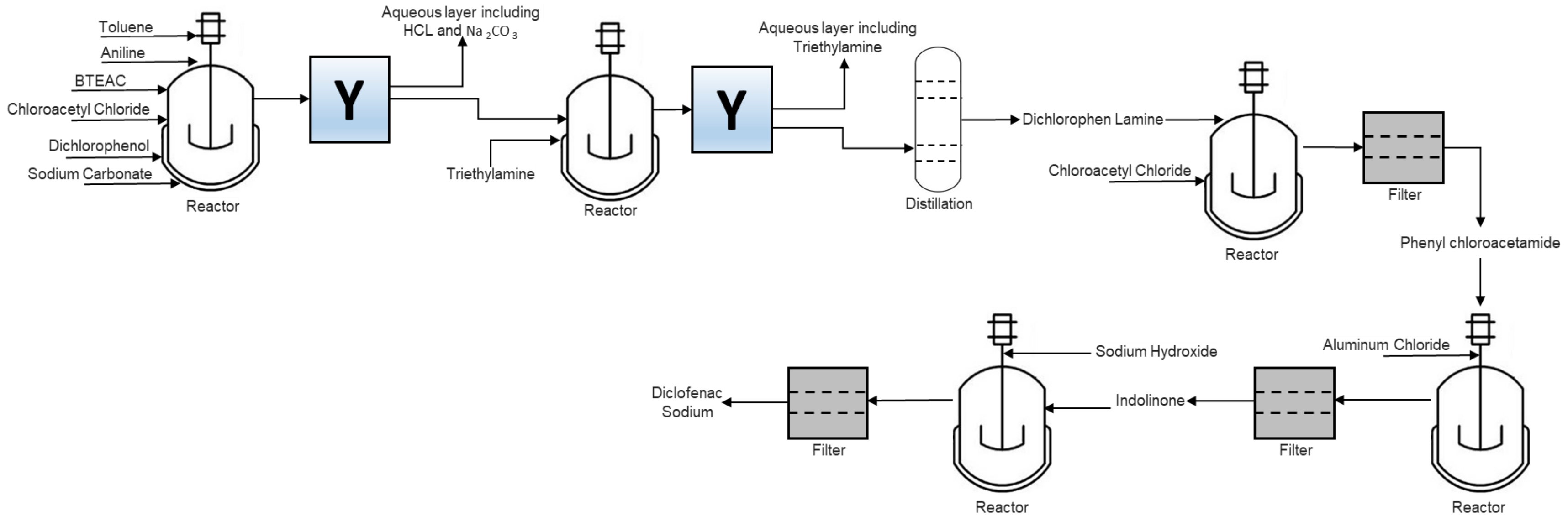

Figure S13. Illustration of diclofenac synthesis.

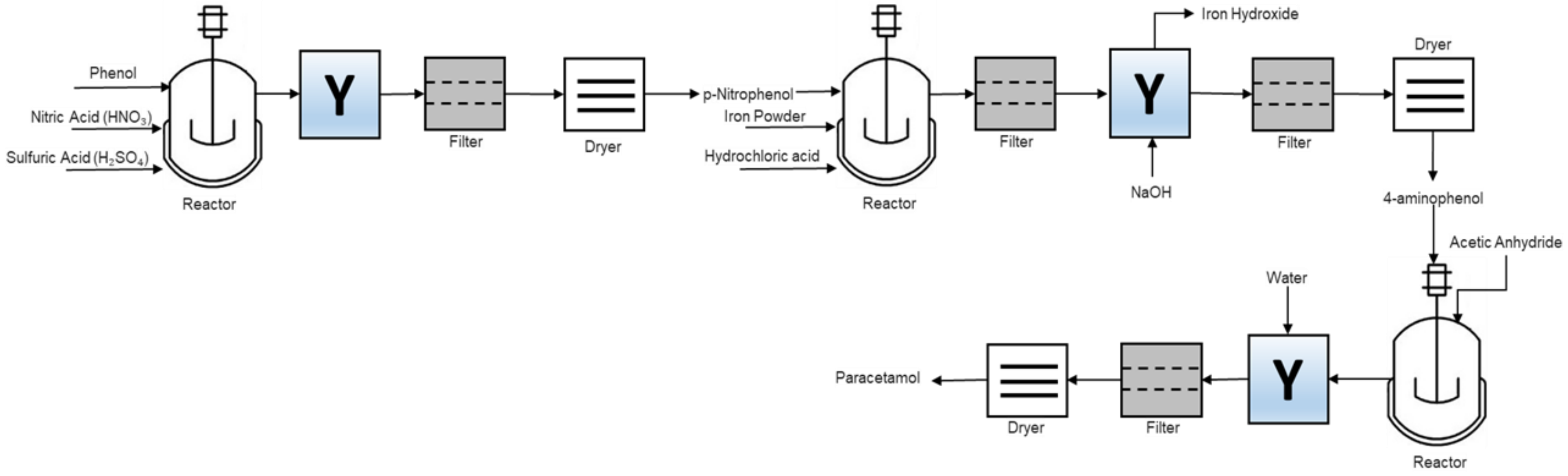

Figure S14. Illustration of paracetamol synthesis.

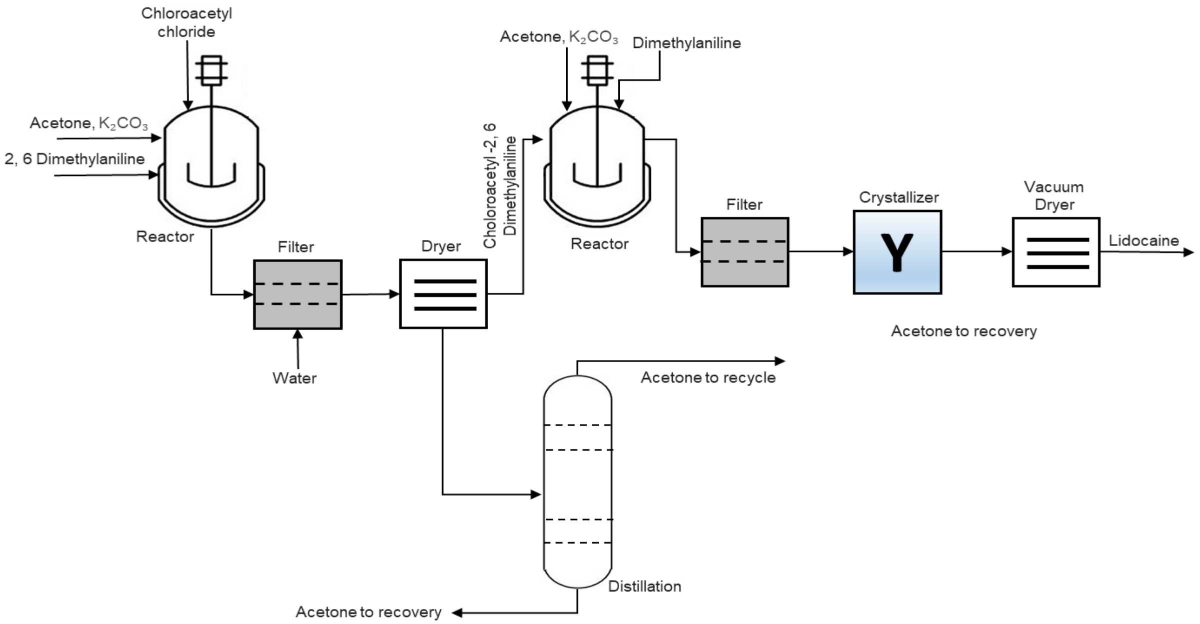

Figure S15. Illustration of lidocaine synthesis.

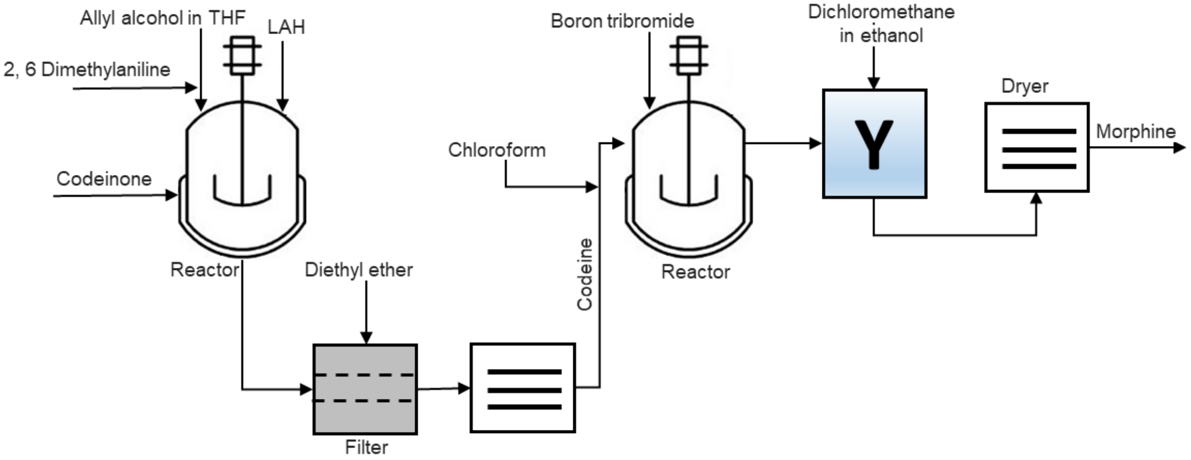

Figure S16. Illustration of morphine synthesis.

8.3 UPSTREAM CHEMICAL MODELLING

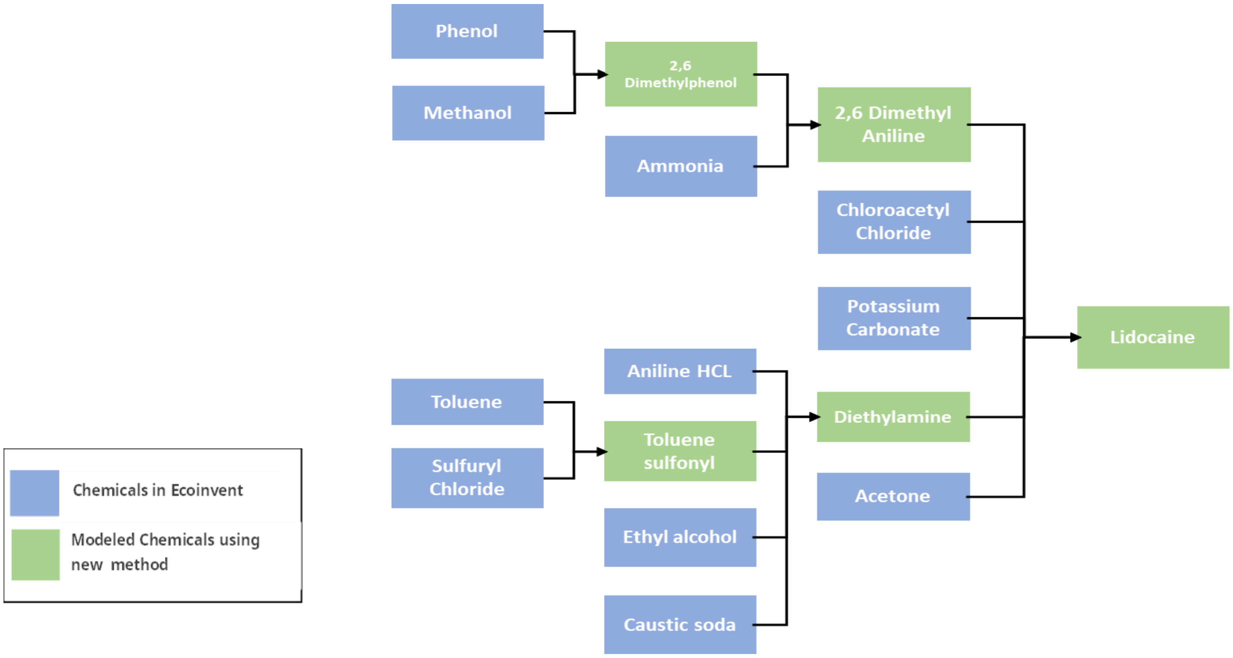

Figure S17. Upstream chemical route for LCI modelling of lidocaine synthesis.

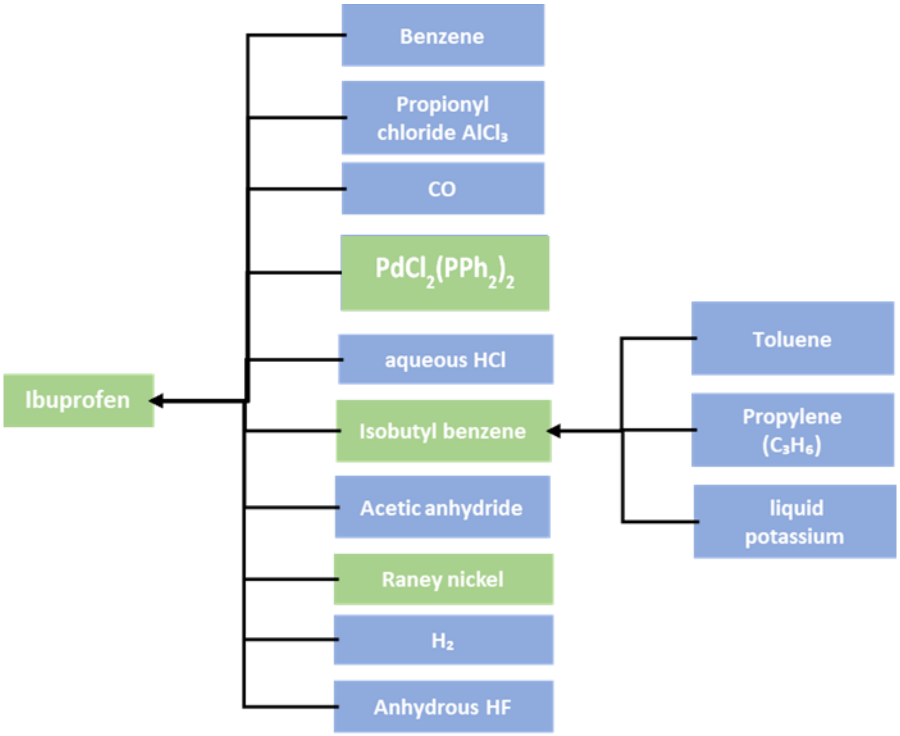

Figure S18. Upstream chemical route for LCI modelling of ibuprofen synthesis.

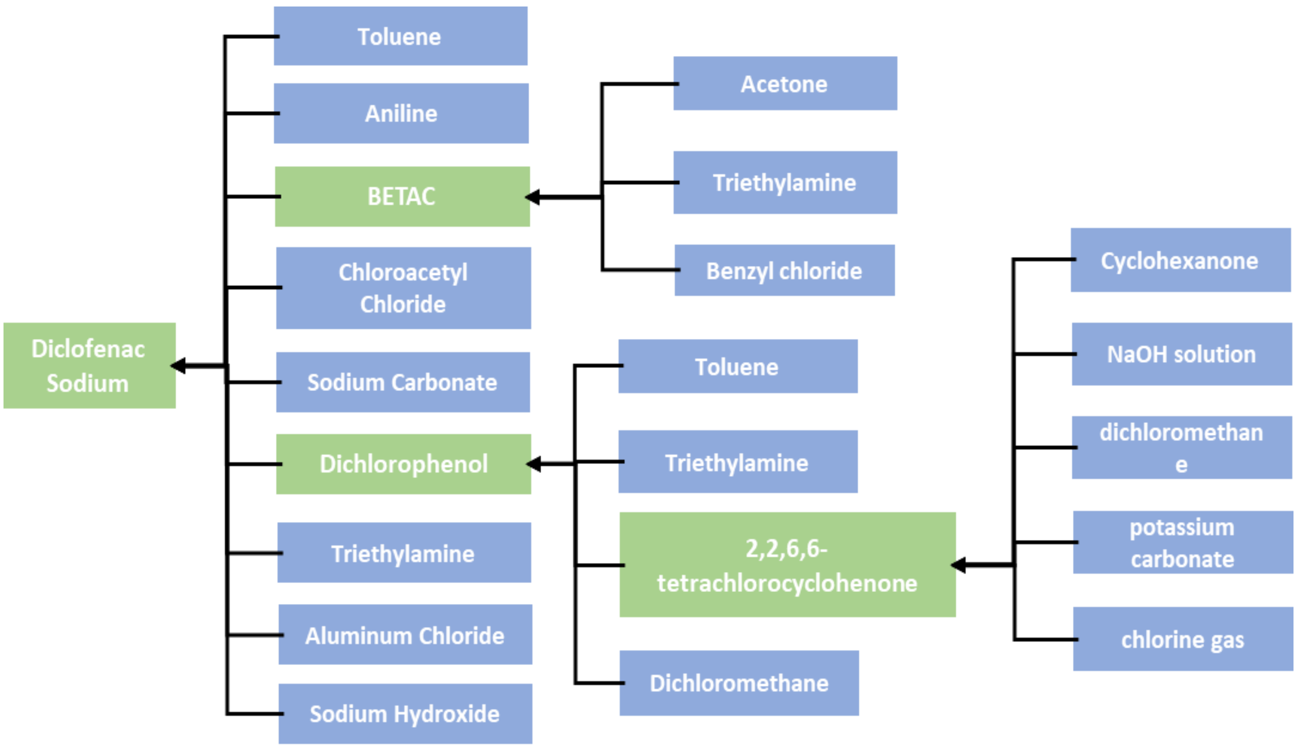

Figure S19. Upstream chemical route for LCI modelling of diclofenac synthesis.

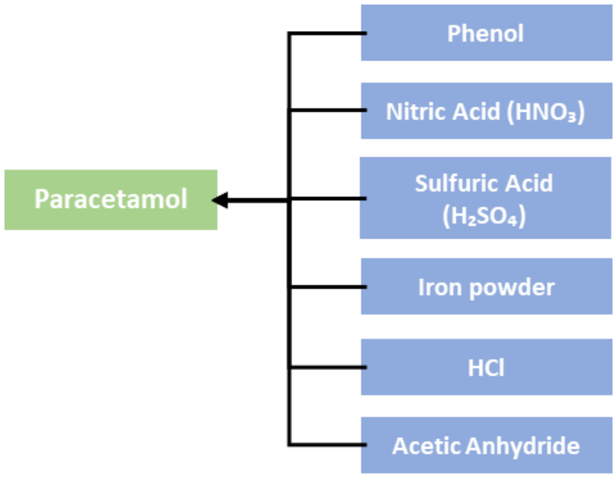

Figure S20. Upstream chemical route for LCI modelling of paracetamol synthesis.

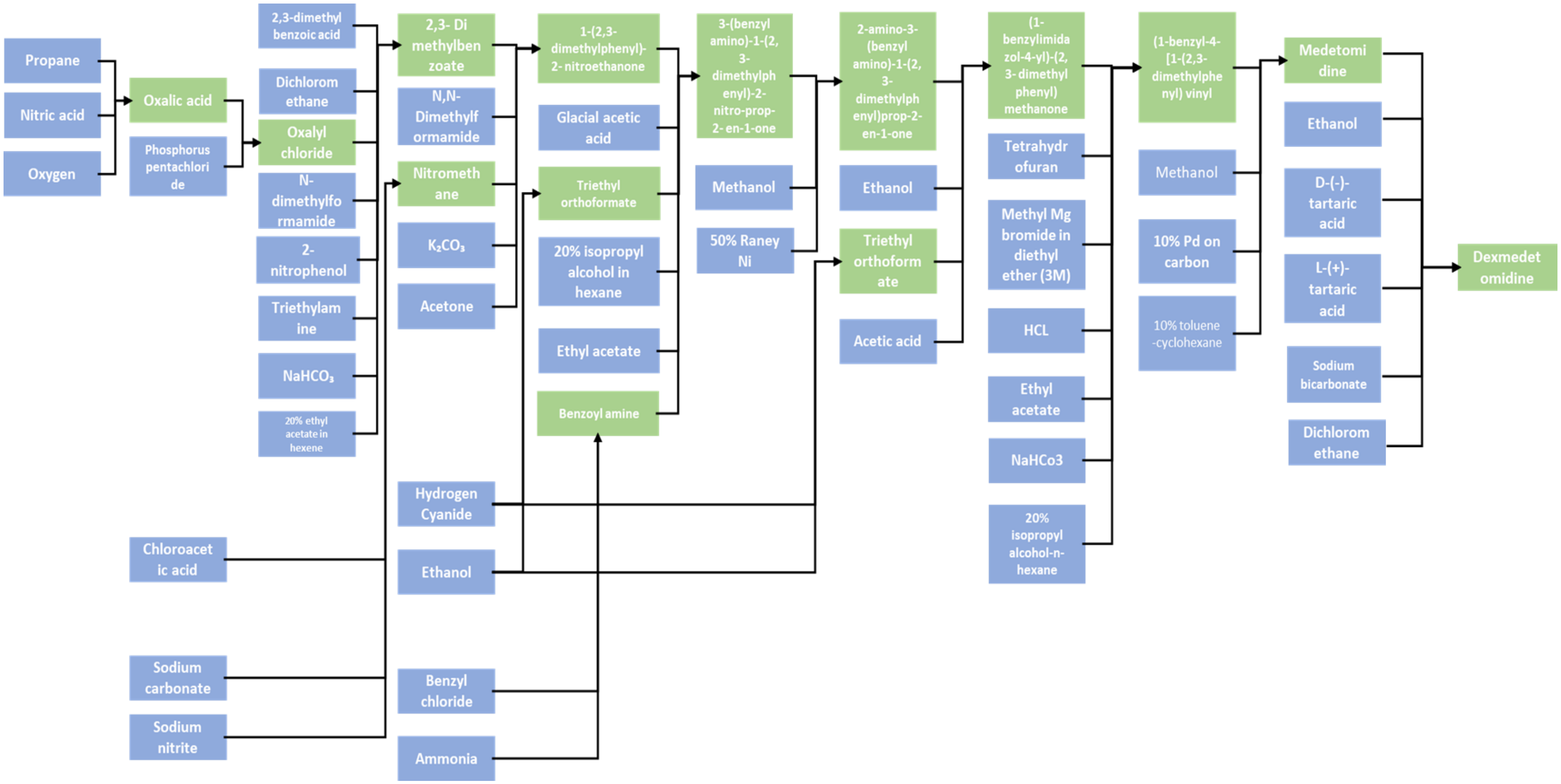

Figure S21. Upstream chemical route for LCI modelling of dexmedetomidine synthesis.

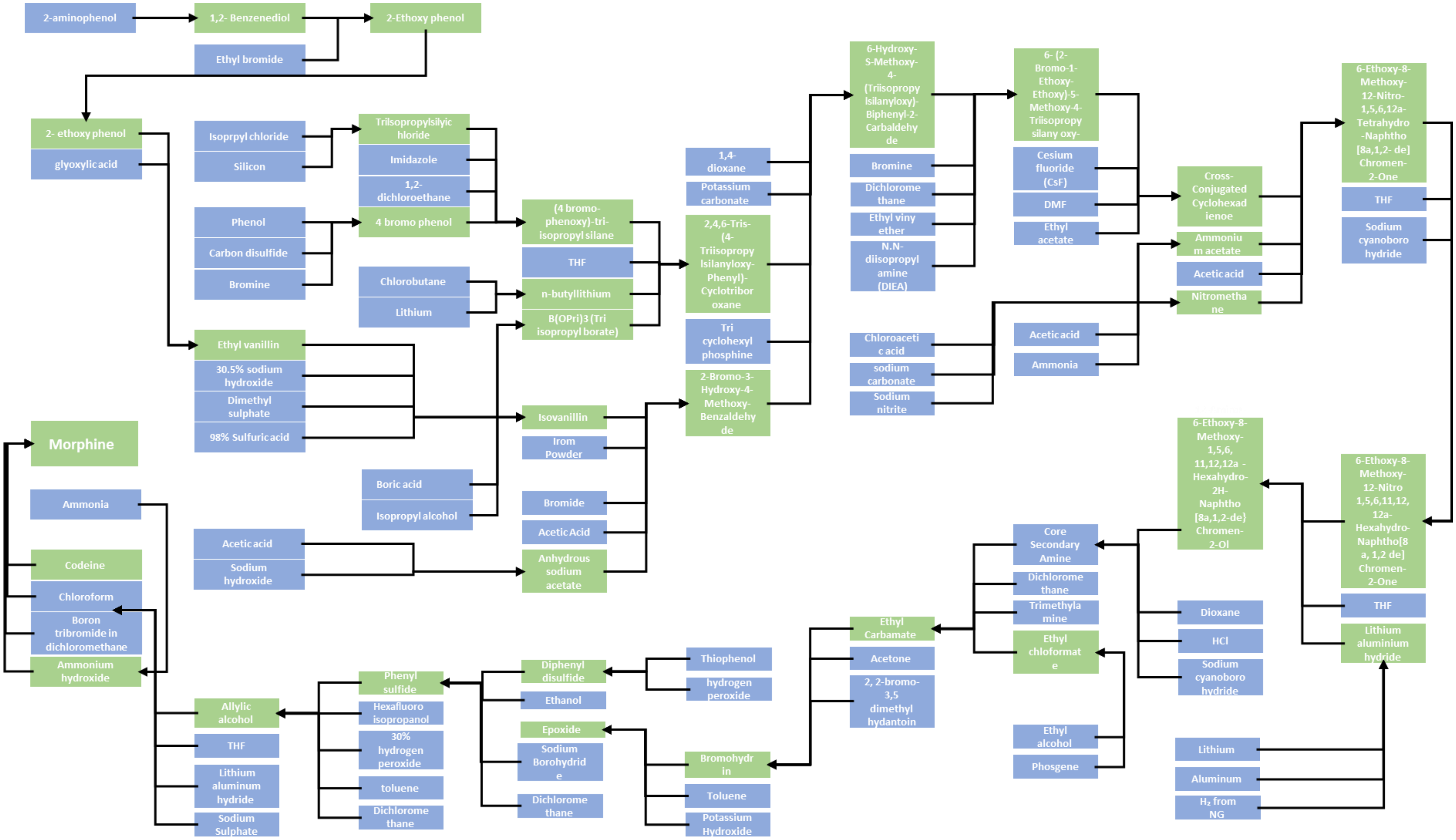

Figure S22. Upstream chemical route for LCI modelling of morphine synthesis.

REFERENCES

1. Niazi, S. F., Handbook of pharmaceutical manufacturing formulations, 2nd ed, Vol. 1:compressed solid products.; CRC Press, 2016.
2. Elango, V.; Murphy, M. A.; Smith, B. L.; Davenport, K. G.; Mott, G. N.; Zey, E. G.; Moss, G. L. Method for Producing Ibuprofen. U.S. Patent 4,981,995, Jan 1, 1991.
3. Jiménez-González, C.; Overcash, M., Energy sub-modules applied in life-cycle inventory of processes. Clean Products and Processes 2000, 2 (1), 57-66.
4. Geisler, G.; Hofstetter, T. B.; Hungerbühler, K., Production of fine and speciality chemicals: procedure for the estimation of LCIs. The International Journal of Life Cycle Assessment 2004, 9 (2), 101-113.
5. Parvatker; Tunceroglu, H.; Sherman, J. D.; Coish, P.; Anastas, P.; Zimmerman, J. B.; Eckelman, M. J., Cradle-to-Gate Greenhouse Gas Emissions for Twenty Anesthetic Active Pharmaceutical Ingredients Based on Process Scale-Up and Process Design Calculations. *ACS Sustainable Chemistry & Engineering* 2019, *7* (7), 6580-6591. DOI:10.1021/acssuschemeng.8b05473
6. Bruni, G.; Martini, C.; Martini, F.; Salvio, M., On the Energy Performance and Energy Saving Potential of the Pharmaceutical Industry: A Study Based on the Italian Energy Audits. *Processes* 2023, *11*, 1114. DOI: 10.3390/pr11041114
7. IAC. *IAC Assessments.* 2024. https://iac.university/searchAssessments?NAICS=3254 (accessed Jun 10, 2024)
8. Bieler, P. S.; Fischer, U.; Hungerbühler, K., Modeling the Energy Consumption of Chemical Batch Plants: Bottom-Up Approach. Industrial & Engineering Chemistry Research 2004, 43 (24), 7785-7795.
9. Piccinno, F.; Hischier, R.; Seeger, S.; Som, C., From laboratory to industrial scale: a scale-up framework for chemical processes in life cycle assessment studies. Journal of Cleaner Production 2016, 135, 1085-1097.
10. De Dietrich Process Systems. *Glass-Lined Reactors.* https://www.dedietrich.com/en/equipment/process/reaction-agitation/glass-lined-reactors (accessed Jul 13, 2024).
11. Büchiglas AG. *Production Scale Reactor Systems: Glass Reactors & Process Equipment.* https://www.buchiglas.com/en/glass-reactors-process-equipment/production-scale-reactor-systems.html (accessed Sept 11, 2024).
12. Rérat, C.; Papadokonstantakis, S.; Hungerbühler, K., Estimation and Analysis of Energy Utilities Consumption in Batch Chemical Industry through Thermal Losses Modeling. Industrial & Engineering Chemistry Research 2012, 51 (31), 10416-10432.
13. Szïjjarto, A.; Papadokonstantakis, S.; Fischer, U.; Hungerbühler, K., Bottom-up Modeling of the Steam Consumption in Multipurpose Chemical Batch Plants Focusing on Identification of the Optimization Potential. Industrial & Engineering Chemistry Research 2008, 47 (19), 7323-7334.

14. Towler, G. P., & Sinnott, R. K., Chemical engineering design: Principles, practice and economics of plant and process design. second edition (2nd ed.) ed.; Butterworth-Heinemann: 2013.

15. Stoker, A. API Chemical Synthesis Trends in Reactor Heat Transfer Design. Org. Process Res. Dev. 2017, 21 (3), 385–399.

16. Wills, A. C.; McKenna, T. Next Generation Batch Reactor Productivity and Efficiency: An Introduction to the PI QFlux™ Batch Reactor System – Form, Function, and Results. *Heat Exchanger World* 2022, October, 47–50. https://www.ddpsinc.com/hubfs/PI%20QFlux%20White%20Paper%20-%20DDPS.pdf

17. McKeown, R. R.; Wertman, J. T.; Dell'Orco, P. C., Crystallization Design and Scale-Up. In Chemical Engineering in the Pharmaceutical Industry, 2010; pp 213-247.

18. Parvatker, A. G.; Tunceroglu, H.; Sherman, J. D.; Coish, P.; Anastas, P.; Zimmerman, J. B.; Eckelman, M. J., Cradle-to-Gate Greenhouse Gas Emissions for Twenty Anesthetic Active Pharmaceutical Ingredients Based on Process Scale-Up and Process Design Calculations. ACS Sustainable Chemistry & Engineering 2019, 7 (7), 6580-6591.

19. Heinkel Process Technology. *Filter Dryer Pharma.* https://www.heinkel.com/product/filter-dryer-pharma/ (accessed July 9, 2024).

20. Separatech. *Agitated Nutsche Filter Dryers (ANFD).* https://www.separatech.com/agitated-nutsche-filter-dryers-anfd/ (accessed Aug 9, 2024).

21. Mujumdar, A. S., Ed. Handbook of Industrial Drying, 3rd ed.; CRC Press, Taylor & Francis Group: Boca Raton, FL, 2006.

22. Tsotsas, E.; Mujumdar, A. S., Eds. Modern Drying Technology, Volume 4: Energy Savings; Wiley-VCH: Weinheim, Germany, 2012.

23. Chen, P.; Ansari, M. J.; Bokov, D.; Suksatan, W.; Rahman, M. L.; Sarjadi, M. S., A review on key aspects of wet granulation process for continuous pharmaceutical manufacturing of solid dosage oral formulations. Arabian Journal of Chemistry 2022, 15 (2), 103598.

24. Faure, A.; York, P.; Rowe, R. C., Process control and scale-up of pharmaceutical wet granulation processes: a review. European Journal of Pharmaceutics and Biopharmaceutics 2001, 52 (3), 269-277.

25. Sampat, C.; Kotamarthy, L.; Bhalode, P.; Chen, Y.; Dan, A.; Parvani, S.; Dholakia, Z.; Singh, R.; Glasser, B. J.; Ierapetritou, M.; Ramachandran, R., Enabling energy-efficient manufacturing of pharmaceutical solid oral dosage forms via integrated techno-economic analysis and advanced process modeling. Journal of Advanced Manufacturing and Processing 2022, 4 (4), e10136.

26. GEA Pharma Systems. *PMA™ High Shear Granulator Series;* GEA Process Engineering: Søborg, Denmark. https://www.gea.com (accessed Aug 10, 2024).

27. Iyer, R. M.; Hegde, S.; DiNunzio, J.; Singhal, D.; Malick, W., The impact of roller compaction and tablet compression on physicomechanical properties of pharmaceutical excipients. Pharmaceutical Development and Technology 2014, 19 (5), 583-592.

28. Sajjia, M.; Shirazian, S.; Kelly, C. B.; Albadarin, A. B.; Walker, G., ANN Analysis of a Roller Compaction Process in the Pharmaceutical Industry. Chemical Engineering & Technology 2017, 40 (3), 487-492.

29. UNZHUO Machinery. *GK Series Roller Compactor*. https://www.jzgranulator.com/gk-series-roller-compactor.html.

30. Yinda Machinery. *Dry Granulator.* https://www.yindamachinery.com/dry-granulator/.

31. Cadmach. *Roller Compactor for Dry Granulation (200-50).* https://cadmach.com/product/roller-compactor-for-dry-granulation-200-50/#:~:text=Max%20Output%20,5.

32. Upmach. *GK Series Roller Compactor.* https://upmach.com/solid-machine/granulator/lg-series-roller-compactor#:~:text=Model%20LG,1600%201600%201600%201800%201800.

33. DIOSNA (via Pharm-Alliance). *CAP Fluid Bed Batch Processor.* https://www.pharm-alliance.com/wp-content/uploads/2024/02/Secheur-a-lit-dair-fluidise.pdf

34. SaintyCo. *FG Fluid-Bed Dryer.* SaintyCo. https://www.saintyco.com/fg-fluid-bed-dryer/

35. NU Pharma Machine. *Fluid Bed Dryer.* https://www.nupharmamachine.com/fluid-bed-dryer/

36. Hindiyeh, M.; Altalafha, T.; Al-Naerat, M.; Saidan, H.; Al-Salaymeh, A.; Sbeinati, L.; Saidan, M.N. Process Modification of Pharmaceutical Tablet Manufacturing Operations: An Eco-Efficiency Approach. Processes 2018, 6, 15. https://doi.org/10.3390/pr6020015

37. Sharma, R. K.; Raju, G.; Sarkar, P.; Singh, H.; Singla, E., Comparing the environmental impacts of paracetamol dosage forms using life cycle assessment. *Environment, Development and Sustainability* 2022, *24* (10), 12446-12466.

38. De Leersnyder, F.; Vanhoorne, V.; Bekaert, H.; Vercruysse, J.; Ghijs, M.; Bostijn, N.; Verstraeten, M.; Cappuyns, P.; Van Assche, I.; Vander Heyden, Y.; Ziemons, E.; Remon, J. P.; Nopens, I.; Vervaet, C.; De Beer, T., Breakage and drying behaviour of granules in a continuous fluid bed dryer: Influence of process parameters and wet granule transfer. European Journal of Pharmaceutical Sciences 2018, 115, 223-232.

39. Rizzi Jr, A. C.; Costa Jr, E. F.; Freire, J. T.; Passos, M. L., Modeling the Drying of Grass Seeds (Brachiaria brizantha) in Fluidized Beds. Drying Technology 2007, 25 (1), 123-134.

40. Poós, T.; Szabó, V., Volumetric Heat Transfer Coefficient in Fluidized-Bed Dryers. Chemical Engineering & Technology 2018, 41 (3), 628-636.

41. Tsotsas, E.; M., K.; and Saage, G., Modeling of Contact Dryers. Drying Technology 2007, 25 (7-8), 1377-1391.

42. Kotamarthy, L.; Metta, N.; Ramachandran, R. Understanding the Effect of Granulation and Milling Process Parameters on the Quality Attributes of Milled Granules Processes [Online], 2020.

43. Hanningfield Process Systems. *Conical Mills (Under-Driven), Version 3.* Hanningfield Process Systems Ltd. https://www.hanningfield.com/wp-content/uploads/2018/06/Hanningfield_Conical_Mills_Under-Driven_V3.pdf

44. Hywell Machinery. *V Powder Mixer.* https://www.hywellco.com/V-Powder-Mixer-pd41108892.html

45. Constable, D. J. C.; Jimenez-Gonzalez, C.; Henderson, R. K., Perspective on Solvent Use in the Pharmaceutical Industry. Organic Process Research & Development 2007, 11 (1), 133-137.

46. Clark, J. H.; Tavener, S. J., Alternative Solvents: Shades of Green. Organic Process Research & Development 2007, 11 (1), 149-155.

47. Jiménez-González, C.; Poechlauer, P.; Broxterman, Q. B.; Yang, B.-S.; am Ende, D.; Baird, J.; Bertsch, C.; Hannah, R. E.; Dell'Orco, P.; Noorman, H.; Yee, S.; Reintjens, R.; Wells, A.; Massonneau, V.; Manley, J., Key Green Engineering Research Areas for Sustainable Manufacturing: A Perspective from Pharmaceutical and Fine Chemicals Manufacturers. Organic Process Research & Development 2011, 15 (4), 900-911.

48. Amelio, A.; Genduso, G.; Vreysen, S.; Luis, P.; Van der Bruggen, B., Guidelines based on life cycle assessment for solvent selection during the process design and evaluation of treatment alternatives. Green Chemistry 2014, 16 (6), 3045-3063.

49. Luis, P.; Amelio, A.; Vreysen, S.; Calabro, V.; Van der Bruggen, B., Life cycle assessment of alternatives for waste-solvent valorization: batch and continuous distillation vs incineration. The International Journal of Life Cycle Assessment 2013, 18 (5), 1048-1061.

50. Meyer, R.; Figueroa Paredes, D. A.; Fuentes, M.; Amelio, A.; Morero, B.; Luis, P.; Van der Bruggen, B.; Espinosa, J., Conceptual model-based optimization and environmental evaluation of waste solvent technologies: Distillation/incineration versus distillation/pervaporation. Separation and Purification Technology 2016, 158, 238-249

51. Rundquist, E. M.; Pink, C. J.; Livingston, A. G., Organic solvent nanofiltration: a potential alternative to distillation for solvent recovery from crystallisation mother liquors. Green Chemistry 2012, 14 (8), 2197-2205.

52. Ramzan, N.; Degenkolbe, S.; Witt, W., Evaluating and improving environmental performance of HC's recovery system: A case study of distillation unit. Chemical Engineering Journal 2008, 140 (1), 201-213.

53. Savelski, M. J.; Slater, C. S.; Tozzi, P. V.; Wisniewski, C. M., On the simulation, economic analysis, and life cycle assessment of batch-mode organic solvent recovery alternatives for the pharmaceutical industry. Clean Technologies and Environmental Policy 2017, 19 (10), 2467-2477.

54. Tarango Brito, E. C.; Barrera Díaz, C. E.; Ávila Córdoba, L. I.; Frontana Uribe, B. A.; Solís Casados, D. A., Recovery and Reuse of Acetone from Pharmaceutical Industry Waste by Solar Distillation. *Processes* 2025, *13* (2), 361.

55. Aboagye, E. A.; Chea, J. D.; Yenkie, K. M., Systems level roadmap for solvent recovery and reuse in industries. *iScience* 2021, *24* (10), 103114.

56. Capello, C.; Hellweg, S.; Badertscher, B.; Hungerbühler, K., Life-Cycle Inventory of Waste Solvent Distillation: Statistical Analysis of Empirical Data. Environmental Science & Technology 2005, 39 (15), 5885-5892.

57. Rodriguez-Donis, I.; Gerbaud, V.; Arias-Barreto, A.; Joulia, X., Heterogeneous Batch Distillation Processes for Waste Solvent Recovery in Pharmaceutical Industry. In Computer Aided Chemical Engineering, de Brito Alves, R. M.; do Nascimento, C. A. O.; Biscaia, E. C., Eds. Elsevier: 2009; Vol. 27, pp 1119-1124.

58. Sun, S.; Yang, A.; Kong, Z. Y.; Huang, H.; Lv, L.; Zhou, Q.; Gu, B., The conceptual design and process intensification of the separation of ternary azeotropic mixture with heterogeneous characteristic. Chemical Engineering Research and Design 2024, 204, 303-315.

59. Cheng, H.; Zhong, J.; Dai, Y.; Jiao, Y.; Zhu, Z.; Cui, P.; Qi, J.; Wang, Y., Design and multiple performance evaluation of green energy saving process for ethyl acetate/ethanol/water azeotrope separation by extractive distillation based on mixed solvent. Journal of Cleaner Production 2023, 421, 138565.

60. Liu, J.; Yin, Y.; Dai, S.; Liu, B.; Wang, Q., Mechanistic Analysis and Process Simulation of Ethyl Acetate-Ethanol Separation by Complex Solvent Extractive Distillation. ACS Omega 2024, 9 (24), 26596-26606.

61. Salmanipour, S.; Ali, A.; Majid, A. S.; and Sokhansanj, A., Separation of a two binary-azeotrope acetonitrile-cyclohexane-toluene ternary mixture via continuous triple column extractive distillation with heat integration: design, simulation, and multi-objective genetic-algorithm (MOGA) optimization. Separation Science and Technology 2023, 58 (14), 2539-2555.

62. Ma, Y.; Hu, Z.; Sun, X.; Shen, Z.; Li, X.; Gao, J.; Zhang, L.; Wang, Y., Energy-saving design of azeotropic distillation for the separation of methanol–methyl methacrylate azeotrope from industrial effluent. Separation and Purification Technology 2025, 353, 128382.

63. Wang, S.; Jia, S.; Gao, Z.; Wang, J.; Gong, J., Highly efficient crystallization for sustainable azeotrope separation of formic acid-Water. Separation and Purification Technology 2024, 342, 126821.

64. Waltermann, T.; Grueters, T.; Skiborowski, M., Optimization of extractive distillation – integrated solvent selection and energy integration. In Computer Aided Chemical Engineering, Eden, M. R.; Ierapetritou, M. G.; Towler, G. P., Eds. Elsevier: 2018; Vol. 44, pp 187-192.

65. Liang, S.; Bu, G., Comparison of different heat integration pressure swing distillation processes for separating isobutanol and p-xylene azeotrope. Scientific Reports 2024, 14 (1), 28472.

66. Wu, T.; Wang, C.; Liu, J.; Zhuang, Y.; Du, J., Design and 4E analysis of heat pump-assisted extractive distillation processes with preconcentration for recovering ethyl-acetate and ethanol from wastewater. Chemical Engineering Research and Design 2024, 201, 510-522.

67. Pan, C.; Guo, J.; Feng, B.; Qiu, X.; Zhu, Q.; Song, H.; Gai, H.; Xiao, M.; Huang, T., Energy-saving extractive distillation process for the separation of close-boiling 2, 6-xylenol and p-cresol mixture. Journal of the Taiwan Institute of Chemical Engineers 2024, 159, 105505.

68. Li, C.; Jiao, Y.; Li, H.; Wang, Y.; Wang, W.; Zou, X.; Zhu, Z.; Li, X.; Wang, Y.; Cui, P., Process design and multi-objective optimization for separation of different feed composition of acetonitrile / ethanol / water with extractive distillation by varying pressure / pervaporation. Separation and Purification Technology 2023, 327, 124921.

69. Novita, F. J.; Lee, H.-Y.; Lee, M., Energy-efficient and ecologically friendly hybrid extractive distillation using a pervaporation system for azeotropic feed compositions in alcohol dehydration process. Journal of the Taiwan Institute of Chemical Engineers 2018, 91, 251-265.

70. Stamp, J.; Majozi, T., Optimum heat storage design for heat integrated multipurpose batch plants. Energy 2011, 36 (8), 5119-5131

71. Ashton, G. (1993). Design of Energy Efficient Batch Processes. In P. A. Pilavachi (Ed.), Energy Efficiency in Process Technology (pp. 1050-1062). Dordrecht: Springer Netherlands.

72. European Cluster Collaboration Platform. Energy Savings Based on Pinch Analysis at a Fine Chemicals or Pharmaceutical Company. https://www.clustercollaboration.eu/content/energy-savings-based-pinch-analysis-pharmaceutical-company

73. Müller, G.; Sugiyama, H.; Stocker, S.; Schmidt, R., Reducing Energy Consumption in Pharmaceutical Production Processes: Framework and Case Study. Journal of Pharmaceutical Innovation 2014, 9 (3), 212-226.

74. Galitsky, C. Energy Efficiency Improvement and Cost Saving Opportunities for the Pharmaceutical Industry.; Lawrence Berkeley National Laboratory: 2008.

75. Hernandez, C.; Rodrigues, C.; Marques, P.; Freire, F., Life cycle assessment of a large volume parenteral for hospital use. Resources, Conservation and Recycling 2023, 198, 107120.

76. IAC, IAC Assessments. U.S Department of Energy: 2024.

77. Becker, J.; Manske, C.; Randl, S., Green chemistry and sustainability metrics in the pharmaceutical manufacturing sector. Current Opinion in Green and Sustainable Chemistry 2022, 33, 100562.

78. Sargent, E. V.; Flueckiger, A.; Barle, E. L.; Luo, W.; Molnar, L. R.; Sandhu, R.; Weideman, P. A., The regulatory framework for preventing cross-contamination of pharmaceutical products: History and considerations for the future. Regulatory Toxicology and Pharmacology 2016, 79, S3-S10.

79. Rogers, L.; Jensen, K. F., Continuous manufacturing-the Green Chemistry promise? Green Chemistry 2019, 21 (13), 3481-3498.

80. Verghese, G.; Lopolito, P. Cleaning Engineering and Equipment Design. In Cleaning and Cleaning Validation, Vol. 1; Pluta, P., Ed.; Davis Healthcare International Publishing: River Grove, IL, 2009; pp 123–152.

81. Palabiyik, I.; Yilmaz, M. T.; Fryer, P. J.; Robbins, P. T.; Toker, O. S., Minimising the environmental footprint of industrial-scaled cleaning processes by optimisation of a novel clean-in-place system protocol. Journal of Cleaner Production 2015, 108, 1009-1018.

82. Aramouni, N. A. K.; Steiner-Browne, M.; Mouras, R., Application of process analytical technology (PAT) in real-time monitoring of pharmaceutical cleaning process: Unveiling the cleaning mechanisms governing the cleaning-in-place (CIP). Process Safety and Environmental Protection 2023, 177, 212-222.

83. Piepiórka-Stepuk, J.; Diakun, J.; Sterczyńska, M.; Kalak, T.; Jakubowski, M., Mathematical modeling and analysis of the interaction of parameters in the clean-in-place procedure during the pre-rinsing stage. Journal of Cleaner Production 2021, 297, 126484.

84. Li, Y.; Zhang, S.; Zhang, W.; Xiong, W.; Ye, Q.; Hou, X.; Wang, C.; Wang, P., Life cycle assessment of advanced wastewater treatment processes: Involving 126 pharmaceuticals and personal care products in life cycle inventory. Journal of Environmental Management 2019, 238, 442-450.

85. Tomatis, M.; Moreira, M. T.; Xu, H.; Deng, W.; He, J.; Parvez, A. M., Removal of VOCs from waste gases using various thermal oxidizers: A comparative study based on life cycle assessment and cost analysis in China. Journal of Cleaner Production 2019, 233, 808-818.

86. Jiménez-González, C.; Overcash, M. R.; Curzons, A., Waste treatment modules – a partial life cycle inventory. Journal of Chemical Technology & Biotechnology 2001, 76 (7), 707-716.

87. Hassim, M. H.; Hurme, M.; Amyotte, P. R.; Khan, F. I., Fugitive emissions in chemical processes: The assessment and prevention based on inherent and add-on approaches. Journal of Loss Prevention in the Process Industries 2012, 25 (5), 820-829.

88. Smith, R. L.; Ruiz-Mercado, G. J.; Meyer, D. E.; Gonzalez, M. A.; Abraham, J. P.; Barrett, W. M.; Randall, P. M., Coupling Computer-Aided Process Simulation and Estimations of Emissions and Land Use for Rapid Life Cycle Inventory Modeling. ACS Sustainable Chemistry & Engineering 2017, 5 (5), 3786-3794.

89. Zhou, L.; Ma, C.; Horlyck, J.; Liu, R.; Yun, J. Development of Pharmaceutical VOCs Elimination by Catalytic Processes in China Catalysts [Online], 2020.

90. Cheng, N.; Jing, D.; Zhang, C.; Chen, Z.; Li, W.; Li, S.; Wang, Q., Process-based VOCs source profiles and contributions to ozone formation and carcinogenic risk in a typical chemical synthesis pharmaceutical industry in China. Science of The Total Environment 2021, 752, 141899.

91. Lin, Q.; Gao, Z.; Zhu, W.; Chen, J.; An, T., Underestimated contribution of fugitive emission to VOCs in pharmaceutical industry based on pollution characteristics, odorous activity and health risk assessment. Journal of Environmental Sciences 2023, 126, 722-733.

92. Gao, L.; Keener, T. C.; Zhuang, L.; Siddiqui, K. F., A technical and economic comparison of biofiltration and wet chemical oxidation (scrubbing) for odor control at wastewater treatment plants. Environmental Engineering and Policy 2001, 2 (4), 203-212.

93. Ankush; Mandal, M. K.; Sharma, M.; Khushboo; Pandey, S.; Dubey, K. K., Membrane Technologies for the Treatment of Pharmaceutical Industry Wastewater. In Water and Wastewater Treatment Technologies, Bui, X.-T.; Chiemchaisri, C.; Fujioka, T.; Varjani, S., Eds. Springer Singapore: Singapore, 2019; pp 103-116.

94. Larsson, D. G. J.; de Pedro, C.; Paxeus, N., Effluent from drug manufactures contains extremely high levels of pharmaceuticals. Journal of Hazardous Materials 2007, 148 (3), 751-755.

95. Mousel, D.; Bastian, D.; Firk, J.; Palmowski, L.; Pinnekamp, J., Removal of pharmaceuticals from wastewater of health care facilities. Science of The Total Environment 2021, 751, 141310.

96. Pistocchi, A.; Andersen, H. R.; Bertanza, G.; Brander, A.; Choubert, J. M.; Cimbritz, M.; Drewes, J. E.; Koehler, C.; Krampe, J.; Launay, M.; Nielsen, P. H.; Obermaier, N.; Stanev, S.; Thornberg, D., Treatment of micropollutants in wastewater: Balancing effectiveness, costs and implications. *Science of The Total Environment* 2022, *850*, 157593

97. Katsoyiannis, I. A.; Canonica, S.; von Gunten, U., Efficiency and energy requirements for the transformation of organic micropollutants by ozone, O3/H2O2 and UV/H2O2. *Water Res* 2011, *45* (13), 3811-22.

98. Hansen, K. M. S.; Andersen, H. R., Energy Effectiveness of Direct UV and UV/H2O2 Treatment of Estrogenic Chemicals in Biologically Treated Sewage. *International Journal of Photoenergy* 2012, *2012* (1), 270320.

99. Cibati, A.; Gonzalez-Olmos, R.; Rodriguez-Mozaz, S.; Buttiglieri, G., Unravelling the performance of UV/H2O2 on the removal of pharmaceuticals in real industrial, hospital, grey and urban wastewaters. *Chemosphere* 2022, *290*, 133315.

100. Feng, L.; van Hullebusch, E. D.; Rodrigo, M. A.; Esposito, G.; Oturan, M. A., Removal of residual anti-inflammatory and analgesic pharmaceuticals from aqueous systems by electrochemical advanced oxidation processes. A review. Chemical Engineering Journal 2013, 228, 944-964

101. Coria, G.; Nava, J. L.; Carreño, G., Electrooxidation of Diclofenac in Synthetic Pharmaceutical Wastewater Using an Electrochemical Reactor Equipped with a Boron Doped Diamond Electrode. ECS Meeting Abstracts 2014, MA2014-02 (18), 937

102. Ensano, B. M. B.; Borea, L.; Naddeo, V.; Belgiorno, V.; de Luna, M. D. G.; Balakrishnan, M.; Ballesteros, F. C., Applicability of the electrocoagulation process in treating real municipal wastewater containing pharmaceutical active compounds. Journal of Hazardous Materials 2019, 361, 367-373.

103. Kabdaşlı, I.; Arslan-Alaton, I.; Ölmez-Hancı, T.; Tünay, O., Electrocoagulation applications for industrial wastewaters: a critical review. Environmental Technology Reviews 2012, 1 (1), 2-45.

104. Chu, L.; Wang, J.; He, S.; Chen, C.; Wojnárovits, L.; Takács, E., Treatment of pharmaceutical wastewater by ionizing radiation: Removal of antibiotics, antimicrobial resistance genes and antimicrobial activity. Journal of Hazardous Materials 2021, 415, 125724.

105. Papavasileiou, V., Koulouris, A., Siletti, C., & Petrides, D. (2006, November 1). The role of process simulation and scheduling tools in the development and manufacture of pharmaceutical products. Pharmaceutical Technology, 2006 Supplement (Issue 7)

106. Glatt Ingenieurtechnik GmbH. From Core to Shell: How Modular Planning Brings Flexibility to Pharmaceutical Production; Glatt Ingenieurtechnik GmbH: Weimar, Germany, 2024.

107. Galitsky, C.; Chang, S.; Worrell, E.; Masanet, E. Energy Efficiency Improvement and Cost Saving Opportunities for the Pharmaceutical Industry: An ENERGY STAR® Guide for Energy and Plant Managers; Lawrence Berkeley National Laboratory: Berkeley, CA, 2008; LBNL-57260-Revision.

108. Bruton, K. 2014. The use of fault detection and diagnostics to reduce air handling unit energy consumption. PhD Thesis, University College Cork.

109. Rihawi, B., The Impact of Multiple Production Lines on the Effectiveness of Quality Management Systems and Internal Controls in Pharmaceutical Manufacturing. Emirati Journal of Business, Economics, & Social Studies 2025, 4 (1), 60-64.

110. CloudRail. Pharma Case Study: CloudRail. CloudRail. https://cloudrail.com/wp-content/uploads/2021/05/cloudrail¬-case¬-study¬-pharma.pdf

111. Cremer, P.; Lösch, M.; Schrader, U. Maximizing Efficiency in Pharma Operations. McKinsey & Company, April 1, 2009. https://www.mckinsey.com/~/media/McKinsey/Business%20Functions/Operations/Our%20

Insights/Maximizing%20efficiency%20in%20pharma%20operations/Maximizing%20efficiency%20in%20pharma%20operations.pdf

112. Tamura, Y., Yakura, T., Shirouchi, Y., & Haruta, J., Oxidative 1, 2-Aryl Migration of Alkyl Aryl Ketones by Using Diacetoxyphenyliodine : Syntheses of Arylacetate, 2-Arylpropanoate, and 2-Arylsuccinate. CHEMICAL & PHARMACEUTICAL BULLETIN 1985, 33(3), 1097-1103. DOI:10.1248/cpb.33.1097

113. Murphy, M. A., Early Industrial Roots of Green Chemistry and the history of the BHC Ibuprofen process invention and its Quality connection. Foundations of Chemistry 2018, 20(2), 121-165. doi:10.1007/s10698-017-9300-9

114. Ma, Y., Zhang, X., Zhu, Z., Wang, Y., Gao, J., & Cui, P., Process intensification and waste minimization for ibuprofen synthesis process. Journal of Cleaner Production 2018, 194, 396-405. DOI:10.1016/j.jclepro.2018.05.131

115. Synthesis method of diclofenac sodium. CN113402408A, 2021.

116. Gräfe, I.; Schickaneder, H.; Ahrens, K.-H. Process for the preparation of 2,6-dichlorodiphenylaminoacetic-acid derivatives. EP0380712B1, 1990.

117. Hsieh, F.; Tsai, H.-L.; Liao, W.; Xu, M.; Yang, C. Method of synthesizing diclofenac sodium. US10662145B2, 2020.

118. Hsieh, F.; Tsai, H.-L.; Liao, W.; Xu, M.; Yang, C. Method of synthesizing diclofenac sodium. US20200055811A1, 2020.

119. Diclofenac sodium synthesis. CN1242984C, 2005.

120. Method for synthesizing diclofenac sodium. CN1580039A, 2005.

121. Synthesis process of diclofenac sodium. CN113429308B, 2023.

122. Harvey, E. A.; Russ, J. F. Nitration process for phenolic compounds. US3668261A, 1972.

123. Perrin, A. Process for the purification of para-nitrophenol. US3954892A, 1976.

124. Alles, H.-U.; Dürholz, F.; Lindner, O.; Skipka, G. Verfahren zur Herstellung von p-Aminophenol (Process for the preparation of p-aminophenol). DE2930754C2, 1982.

125. Schulman, H.; Baron, F. A.; Weinberg, A. E. Preparation of N-acetyl-p-aminophenol. US3917695A, 1975.

126. Method for directly synthesizing p-acetamidophenol from nitrobenzene in acetic acid solution in one step. CN104628592B, 2017.

127. Method for directly synthesizing p-acetamidophenol from nitrobenzene in acetic acid solution in one step. CN104628592A, 2015.

128. Stepwise reduction of p-nitrophenol. US4264525A, 1981.

129. Cox, W. L. Preparation of nitrophenols. US3283011A, 1966.

130. Fritch, J. R.; Fruchey, O. S. Production of acetaminophen. US5155273A, 1992.

131. Preparation process of p-acetaminophenol. CN101298425B, 2012.

132. Reilly, T. J., The Preparation of Lidocaine. Journal of Chemical Education 1999, 76 (11), 1557.

133. Method for Preparing Lidocaine. CN102070483B, 2013.

134. Method for Preparing Lidocaine Intermediate α-Chloroacetyl-2,6-Dimethylaniline and Lidocaine without Adding Additional Alkali. WO2021159754A1, 2021.

135. Method for Preparing Lidocaine Intermediate α-Chloroacetyl-2,6-Dimethylaniline and Lidocaine without Adding Additional Alkali. CN111253273A, 2020.

136. Synthesis Method of Lidocaine Intermediate Chloroacetyl-2,6-Dimethylaniline. CN110938012A, 2020.

137. Process for the Preparation of Dexmedetomidine. WO2013069025, 2013.

138. Diker, K.; Gorins, G.; Ozanne-Beaudenon, A.; Goldstein, C. Method for Preparing Dexmedetomidine. EP4055009B1, 2023.

139. Savola, J.-M.; Sirviö, J.; Saano, V. Optical Isomer of an Imidazole Derivative Medetomidine as an Alpha-2-Receptor Agonist. US4910214, 1990.

140. Magnus, P. D.; Fauber, B. P.; Sane, N. Efficient Synthesis of Morphine and Codeine. US8293927, 2012.

141. A Process for the Extraction of Alkaloids from Poppy Straw and/or Capsules. GB713689A, 1954
